# Exploring Links Between Psychosis and Frontotemporal Dementia Using Multimodal Machine Learning: Dementia Praecox Revisited

**DOI:** 10.1001/jamapsychiatry.2022.2075

**Published:** 2022-08-03

**Authors:** Nikolaos Koutsouleris, Christos Pantelis, Dennis Velakoulis, Philip McGuire, Dominic B. Dwyer, Maria-Fernanda Urquijo-Castro, Riya Paul, Sen Dong, David Popovic, Oemer Oeztuerk, Joseph Kambeitz, Raimo K. R. Salokangas, Jarmo Hietala, Alessandro Bertolino, Paolo Brambilla, Rachel Upthegrove, Stephen J. Wood, Rebekka Lencer, Stefan Borgwardt, Carlo Maj, Markus Nöthen, Franziska Degenhardt, Maryna Polyakova, Karsten Mueller, Arno Villringer, Adrian Danek, Klaus Fassbender, Klaus Fliessbach, Holger Jahn, Johannes Kornhuber, Bernhard Landwehrmeyer, Sarah Anderl-Straub, Johannes Prudlo, Matthis Synofzik, Jens Wiltfang, Lina Riedl, Janine Diehl-Schmid, Markus Otto, Eva Meisenzahl, Peter Falkai, Matthias L. Schroeter

**Affiliations:** 1 Department of Psychiatry and Psychotherapy, Ludwig Maximilian University, Munich, Germany; 2 Institute of Psychiatry, Psychology and Neuroscience, King’s College London, London, United Kingdom; 3 Max-Planck Institute of Psychiatry, Munich, Germany; 4 Melbourne Neuropsychiatry Centre, University of Melbourne, Melbourne, Australia; 5 Department of Psychiatry and Psychotherapy, University of Cologne, Cologne, Germany; 6 Department of Psychiatry, University of Turku, Turku, Finland; 7 Department of Basic Medical Science, Neuroscience and Sense Organs, University of Bari Aldo Moro, Bari, Italy; 8 Department of Neurosciences and Mental Health, Fondazione IRCCS Ca’ Granda Ospedale Maggiore Policlinico, Milan, Italy; 9 Department of Pathophysiology and Transplantation, University of Milan, Milan, Italy; 10 Institute of Mental Health, University of Birmingham, Birmingham, United Kingdom; 11 Early Intervention Service, Birmingham Women’s and Children’s NHS Foundation Trust, Birmingham, United Kingdom; 12 School of Psychology, University of Birmingham, Birmingham, United Kingdom; 13 Centre for Youth Mental Health, University of Melbourne, Melbourne, Australia; 14 Orygen, Melbourne, Australia; 15 Department of Psychiatry and Psychotherapy, University of Lübeck, Lübeck, Germany; 16 Institute for Translational Psychiatry, University Muenster, Muenster, Germany; 17 Department of Psychiatry, University Psychiatric Clinics (UPK), University of Basel, Basel, Switzerland; 18 Institute for Genomic Statistics and Bioinformatics, University Hospital Bonn, University of Bonn, Bonn, Germany; 19 Institute of Human Genetics, School of Medicine, University Hospital Bonn, University of Bonn, Bonn, Germany; 20 Department of Child and Adolescent Psychiatry, Psychosomatics and Psychotherapy, University Hospital Essen, University of Duisburg-Essen, Duisburg, Germany; 21 Department of Neurology, Max Planck Institute for Human Cognitive and Brain Sciences, Leipzig, Germany; 22 Clinic for Cognitive Neurology, University Hospital Leipzig, Leipzig, Germany; 23 Department of Neurology, Ludwig Maximilian University Munich, Munich, Germany; 24 Department of Neurology, Saarland University Hospital, Homburg, Germany; 25 Department of Psychiatry and Psychotherapy, University Hospital Bonn, Bonn, Germany; 26 German Center for Neurodegenerative Diseases (DZNE), Göttingen, Germany; 27 Department of Psychiatry and Psychotherapy, University Hospital Hamburg, Hamburg, Germany; 28 Department of Psychiatry and Psychotherapy, Friedrich Alexander University Erlangen-Nuremberg, Erlangen, Germany; 29 Department of Neurology, University of Ulm, Ulm, Germany; 30 Department of Neurology, University Medicine Rostock, Rostock, Germany; 31 Department of Neurodegenerative Diseases, Center of Neurology, Hertie Institute for Clinical Brain Research, Tübingen, Germany; 32 Department of Psychiatry and Psychotherapy, Medical University Göttingen, Göttingen, Germany; 33 Department of Psychiatry and Psychotherapy, Technical University of Munich, Munich, Germany; 34 Department of Psychiatry and Psychotherapy, Medical Faculty, Heinrich-Heine University, Düsseldorf, Germany

## Abstract

**Importance:**

The behavioral and cognitive symptoms of severe psychotic disorders overlap with those seen in dementia. However, shared brain alterations remain disputed, and their relevance for patients in at-risk disease stages has not been explored so far.

**Objective:**

To use machine learning to compare the expression of structural magnetic resonance imaging (MRI) patterns of behavioral-variant frontotemporal dementia (bvFTD), Alzheimer disease (AD), and schizophrenia; estimate predictability in patients with bvFTD and schizophrenia based on sociodemographic, clinical, and biological data; and examine prognostic value, genetic underpinnings, and progression in patients with clinical high-risk (CHR) states for psychosis or recent-onset depression (ROD).

**Design, Setting, and Participants:**

This study included 1870 individuals from 5 cohorts, including (1) patients with bvFTD (n = 108), established AD (n = 44), mild cognitive impairment or early-stage AD (n = 96), schizophrenia (n = 157), or major depression (n = 102) to derive and compare diagnostic patterns and (2) patients with CHR (n = 160) or ROD (n = 161) to test patterns’ prognostic relevance and progression. Healthy individuals (n = 1042) were used for age-related and cohort-related data calibration. Data were collected from January 1996 to July 2019 and analyzed between April 2020 and April 2022.

**Main Outcomes and Measures:**

Case assignments based on diagnostic patterns; sociodemographic, clinical, and biological data; 2-year functional outcomes and genetic separability of patients with CHR and ROD with high vs low pattern expression; and pattern progression from baseline to follow-up MRI scans in patients with nonrecovery vs preserved recovery.

**Results:**

Of 1870 included patients, 902 (48.2%) were female, and the mean (SD) age was 38.0 (19.3) years. The bvFTD pattern comprising prefrontal, insular, and limbic volume reductions was more expressed in patients with schizophrenia (65 of 157 [41.2%]) and major depression (22 of 102 [21.6%]) than the temporo-limbic AD patterns (28 of 157 [17.8%] and 3 of 102 [2.9%], respectively). bvFTD expression was predicted by high body mass index, psychomotor slowing, affective disinhibition, and paranoid ideation (*R*^2^ = 0.11). The schizophrenia pattern was expressed in 92 of 108 patients (85.5%) with bvFTD and was linked to the *C9orf72* variant, oligoclonal banding in the cerebrospinal fluid, cognitive impairment, and younger age (*R*^2^ = 0.29). bvFTD and schizophrenia pattern expressions forecasted 2-year psychosocial impairments in patients with CHR and were predicted by polygenic risk scores for frontotemporal dementia, AD, and schizophrenia. Findings were not associated with AD or accelerated brain aging. Finally, 1-year bvFTD/schizophrenia pattern progression distinguished patients with nonrecovery from those with preserved recovery.

**Conclusions and Relevance:**

Neurobiological links may exist between bvFTD and psychosis focusing on prefrontal and salience system alterations. Further transdiagnostic investigations are needed to identify shared pathophysiological processes underlying the neuroanatomical interface between the 2 disease spectra.

## Key Points

**Question:**

Do psychosis and dementia share brain-behavioral alterations?

**Findings:**

In this diagnostic/prognostic study including 1870 patients, patients with schizophrenia expressed the neuroanatomical pattern of behavioral-variant frontotemporal dementia more strongly (41%) than that of Alzheimer disease (17%), and at lower levels, this difference was also encountered in those with major depression (22% vs 3%). Already in clinical high-risk states for psychosis the high expression of the behavioral-variant frontotemporal dementia pattern was linked to severe phenotypes, unfavorable courses, and elevated polygenic risks for schizophrenia and dementia, with further pattern progression being present in those patients who did not recover over time.

**Meaning:**

Dementia praecox should be revisited as a shared pathophysiological dimension of severe psychosis and frontotemporal disease spectra.

## Teaser

This diagnostic/prognostic study uses machine learning to quantify and compare the expression of structural magnetic resonance imaging patterns of behavioral-variant frontotemporal dementia, Alzheimer disease, and schizophrenia.

## Introduction

*Schizophrenia* remains in use as umbrella term for a heterogeneous group of disorders.^1^ Up to 25% of patients exhibit a course of profound cognitive functional decline, and this observation inspired Emil Kraepelin’s concept of dementia praecox more than a century ago.^2,3^ Based on his pathological examinations, Kraepelin conceptualized dementia praecox as a frontotemporal disorder, but its pathological basis remained elusive.^4^ Recent studies reported a 5-fold increased risk of dementia in schizophrenia^5,6^ although postmortem data did not identify Alzheimer disease (AD) or frontotemporal dementia (FTD) pathology in schizophrenia.^7^

Still, frontotemporolimbic alterations and cognitive deficits characterize schizophrenic psychoses^8^ and evolve early in the disease,^3,9,10^ supporting the possibility of both neurodevelopmental and neuroprogressive brain processes.^11,12,13,14^ In addition, temporal, prefrontal, and insular abnormalities predict poor illness courses in psychotic^15,16,17,18^ and also depressive disorders.^19^ Several research lines point to clinical, neuroimaging, pathological, and genetic similarities between schizophrenia and FTD, particularly behavioral-variant FTD (bvFTD).^20,21,22,23,24,25^ The idea of shared neurobiology between psychiatric disorders and bvFTD initially gained traction because of the clinical similarity and diagnostic conundrum caused by the early onset of bvFTD, its prominent negative symptoms, and disinhibited and psychotic manifestations.^26,27,28^ The finding that Chromosome 9 open reading frame 72 (*C9orf72*) variants—the most frequent genetic cause of bvFTD^29^—are associated with psychotic and affective disorders in carriers and relatives^30^ further spurred interest in the association between bvFTD and psychiatric conditions.^28^ *C9orf72* has been linked with an earlier, neuroinflammation-associated onset,^31^ slower progression of bvFTD,^32^ and prominent psychotic phenotypes.^33^ Also, genetic studies of bvFTD have implicated immune system alterations,^34,35^ echoing findings in schizophrenia.^36,37^ These observations hint at complex overlaps between both conditions, not determined by single genes, localized brain alterations, or isolated symptoms.

Machine learning may facilitate a system-level analysis of these overlaps by encapsulating the neurobiological signatures of bvFTD and schizophrenia into mathematical models.^38^ Disease similarities and differences can be mapped at the clinical and neurobiological scales via crossover model application. Diagnostic specificity can be further interrogated by applying models across psychotic, affective, and neurodegenerative conditions and associating them to Brain Age Gap Estimation (BrainAGE), a marker of accelerated aging in neurological and psychiatric disorders.^39,40^ Furthermore, the genetic underpinnings of models’ predictions can be tested to identify shared and unique features of genetic risk projecting onto brain phenotypes.^41^ Finally, by applying models to longitudinal data, the heterogeneity of illness courses can be investigated along neurodegenerative and psychotic disease dimensions.

Using this approach, we tested (1) whether patients with schizophrenia expressed neuroanatomical patterns of bvFTD more prominently than AD patterns, more strongly than patients with major depression (MD), and more pronouncedly in the presence of negative and disinhibitory symptoms; and (2) whether schizophrenia patterns expressed by patients with bvFTD were predicted by psychopathological features, *C9orf72* status, and neuroinflammatory markers.^31,35,42^ We explored whether disease pattern expression was associated with 2-year functioning in young patients with clinical high-risk (CHR) states for psychosis or recent-onset depression (ROD) and predicted by polygenic risk for FTD, AD, and schizophrenia. Conversely, we evaluated whether functional nonrecovery was linked to 1-year pattern progression in these patients. We also tested whether a structural magnetic resonance imaging (MRI)–based prognostic model of nonrecovery validated diagnostic patterns by predicting neurodegenerative and psychiatric disease expression in the case-control samples. Finally, to probe diagnostic specificity longitudinally, we explored whether neurodegenerative, schizophrenic, or nonrecovery patterns predicted cognitive decline in patients with mild cognitive impairment (MCI) or early-stage AD.

## Methods

The eMethods section in Supplement 1 details our methods, and eFigure 1 in Supplement 1 schematically describes the overall analysis process. Each patient, participant, caregiver, or legal representative provided written informed consent in the projects providing data for the study. Local research ethics committees approved projects according to the principles of the Declaration of Helsinki. The study followed the Transparent Reporting of a Multivariable Prediction Model for Individual Prognosis or Diagnosis (TRIPOD) reporting guideline.

**Figure 1.**
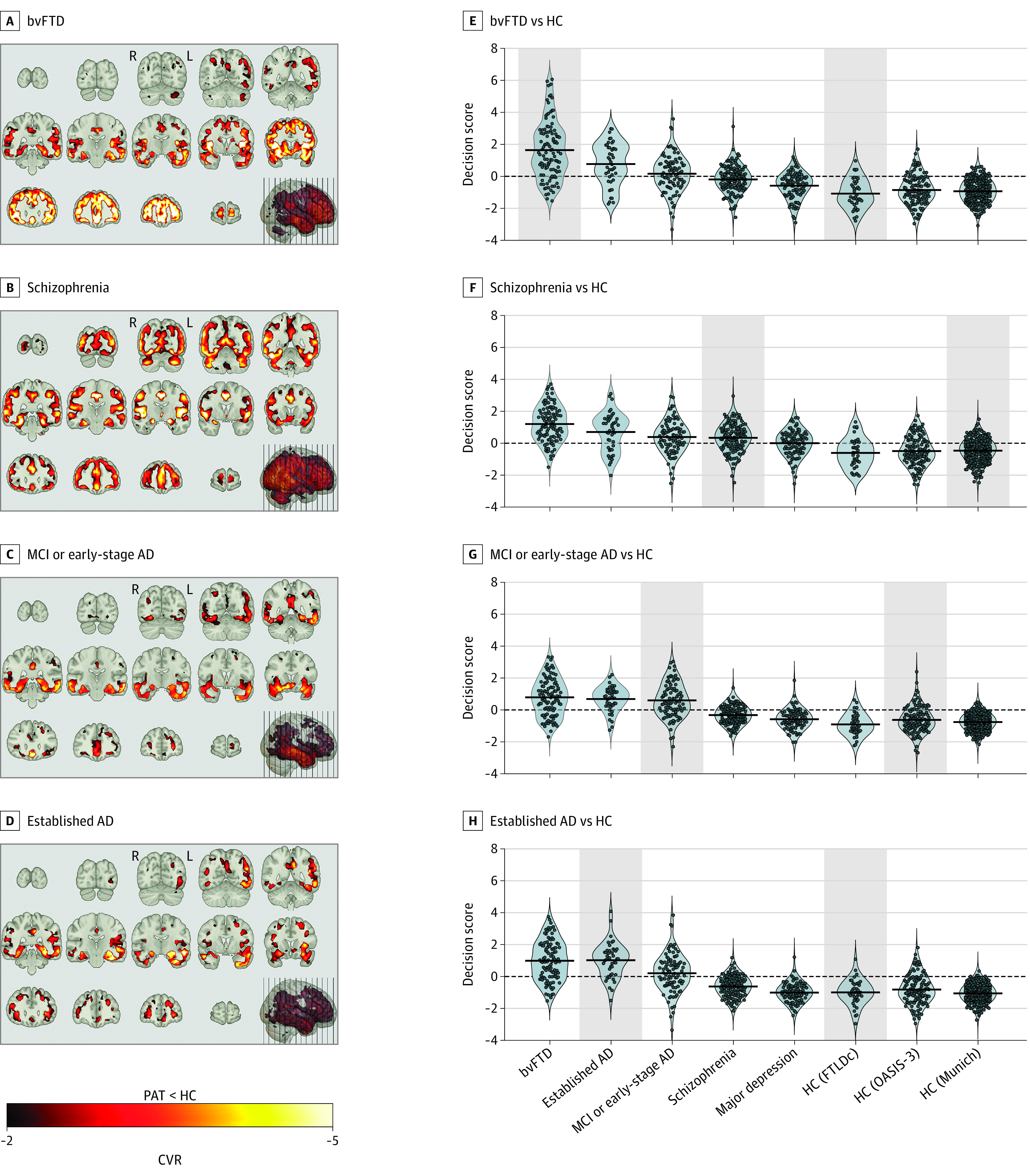
Classifier Signatures, Pattern Score Distributions, and Predicted Case Label Probabilities of Patient (PAT) and Healthy Control (HC) Samples A-D, Cross-validation ratio (CVR) (eMethods in Supplement 1) maps overlaid on the MNI single-individual neuroanatomical template indicate patterns of reliable volume reductions (from top to bottom) in the 4 patient samples vs HC. E-H, Violin plots of pattern score distributions produced by the cross-validated within-group application of classification models (gray background) or the crossover application of models to the other diagnostic groups. Decision score refers to the mean output across all support-vector machine classifiers in the given classification analysis produced for the patients/controls in the given sample. Additionally, the Table describes each study group’s probability of being assigned to the respective patient class by the respective classifier. AD indicates Alzheimer disease; bvFTD, behavioral-variant frontotemporal dementia; MCI, mild cognitive impairment; FTLDc, the German Frontotemporal Lobar Degeneration Consortium.

In summary, we used the machine learning software NeuroMiner version 1.05 to generate 4 diagnostic classifiers differentiating between healthy controls (HCs) from patients with bvFTD (n = 108), established AD (n = 44), MCI or early-stage AD (n = 96), and schizophrenia (n = 157) based on age-standardized and cohort-adjusted gray matter volume maps to facilitate transdiagnostic comparisons across the life span (eTable 1 and eFigure 2 in Supplement 1). Gray matter volume maps were extracted from T1-weighted structural MRI scans (eTable 2 in Supplement 1). Trained diagnostic classifiers (eTable 4 in Supplement 1) were applied to all individuals who had not been involved in model derivation. Post hoc analyses were conducted to compare classifiers (McNemar and Quade tests), evaluate possible determinants of classifier predictions (ie, χ^2^ tests, linear and cubic regression, *t* test, and analysis of variance), and explore their prognostic associations (linear mixed effects models). These analyses were performed in MATLAB version 2020a (MathWorks) and SPSS version 26 (IBM). Statistical significance was determined at α = .05 and 2-sided P values were corrected for multiple comparisons using the false discovery rate and Dunn-Sidak method for pairwise post-hoc tests as required by the respective analysis. Classifiers’ diagnostic signatures, decision score distributions, and patient class predictions were compared in Figure 1, the Table, and eFigures 3 to 4 in Supplement 1 and related to univariate measures in eFigures 5 to 6 in Supplement 1. Then, classifiers’ predictions were probed for confounds, including cohort provenance (eFigure 7 in Supplement 1); age, sex, image quality ratings, and total gray matter volume (eFigure 8 and eTable 5 in Supplement 1); patient subgroup effects (eFigures 9 and 10 in Supplement 1); and spatial nonspecificity, given the varying global brain atrophy present across disease cohorts (eFigure 11 in Supplement 1). Classifiers’ predictions were assessed for nonequality in each patient group (eTable 6 in Supplement 1). In addition, we produced BrainAGE predictions for participants using NeuroMiner to evaluate the specificity of findings against the transdiagnostic effects of accelerated aging (eFigures 12 and 13 in Supplement 1).^43^ This analysis phase was completed by univariate analyses performed with Statistical Parametric Mapping (SPM12, Wellcome Department of Imaging Neuroscience, University College London) that contrasted topographies of diagnostic signatures while controlling for confounds (eFigures 14 and 15 in Supplement 1).

**Table.** Classifier-Specific Patient Label Assignments and Assignment Probabilities Across the Case-Control Samples of the Study

Case-label assignment	No. (%)							
	bvFTD (n = 108)	Established AD (n = 44)	MCI or early-stage AD (n = 96)	Schizophrenia (n = 157)	Major depression (n = 102)	HC (FTLDc) (n = 40)	HC (OASIS-3) (n = 138)	HC (Munich) (n = 335)
bvFTD vs HC classifier	87 (80.6)^a^	32 (72.7)	54 (56.3)	65 (41.4)	22 (21.6)	4 (10.0)^a^	21 (15.2)	30 (9.0)
Schizophrenia vs HC classifier	92 (85.5)	31 (70.5)	63 (65.6)	105 (66.9)^a^	55 (53.9)	11 (27.5)	42 (30.4)	85 (25.4)^a^
MCI or early-stage AD vs HC classifier	80 (74.5)	37 (84.1)	67 (69.8)^a^	47 (29.9)	12 (11.8)	4 (10.0)	29 (21.0)^a^	14 (4.2)
Established AD vs HC classifier	80 (74.5)	35 (79.5)^a^	62 (64.6)	28 (17.8)	3 (2.9)	3 (7.5)^a^	13 (9.4)	6 (1.8)

Abbreviations: AD, Alzheimer disease; bvFTD, behavioral-variant frontotemporal dementia; FTLDc, the German Frontotemporal Lobar Degeneration Consortium; HC, healthy control; MCI, mild cognitive impairment.

^a^ Derivation samples of the respective diagnostic classifier.

**Figure 2.**
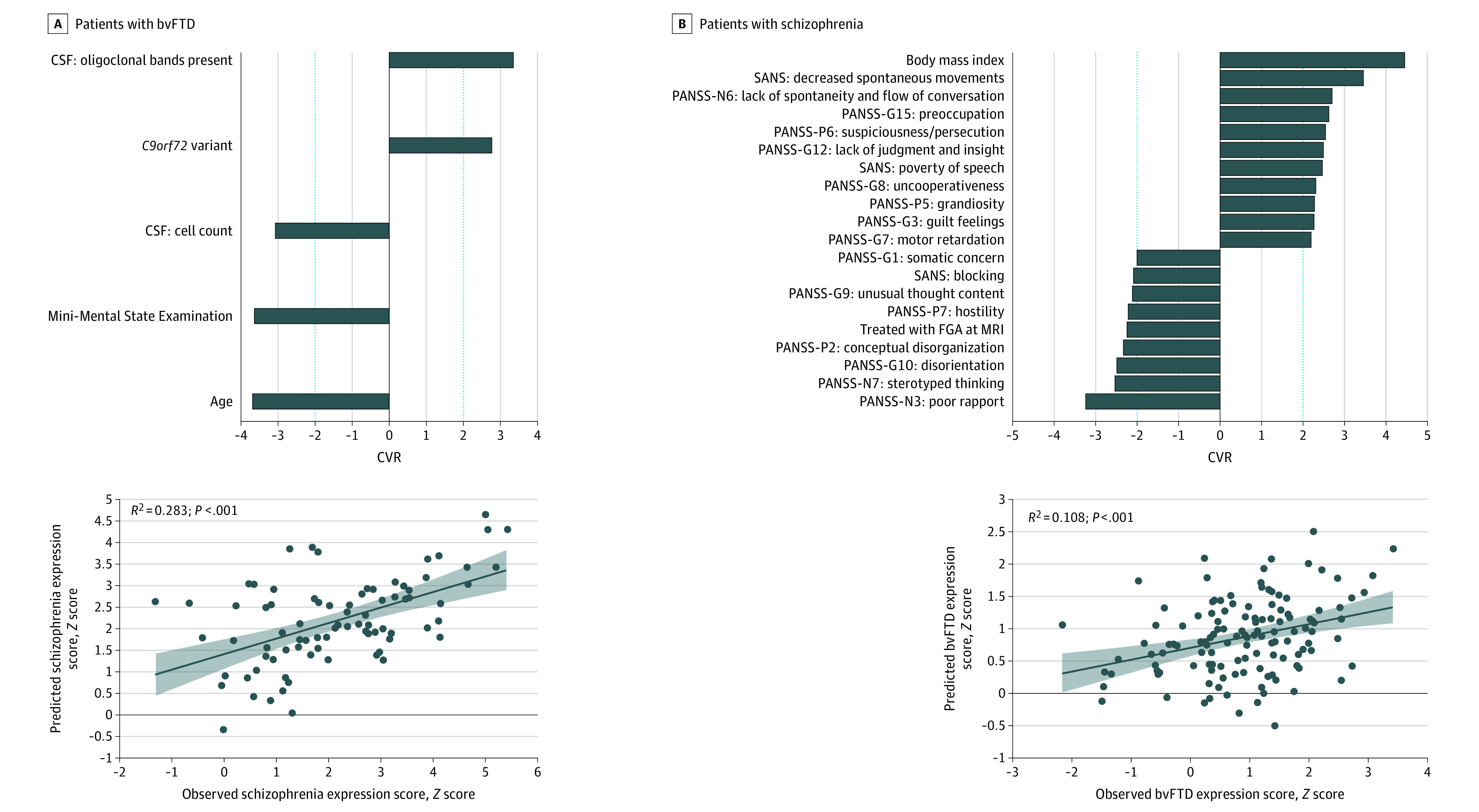
Support-Vector Regression Models Predicting the Neuroanatomical Expression of the Schizophrenia Signature in Patients With Behavioral-Variant Frontotemporal Dementia (bvFTD) and the bvFTD Signature in Patients With Schizophrenia Bar plots show the ranked reliability (cross-validation ratio [CVR]) (eMethods in Supplement 1) of features informing the support-vector regression models’ predictions at |CVR| ≥ 2. Positive and negative CVR values indicate positive and negative predictive associations between features and observed scores. Scatterplots with linear fits, 95% CIs, and coefficients of determination (*R*^2^) describe the accuracy of the respective models in predicting neuroanatomical expression scores. CSF indicates cerebrospinal fluid; FGA, first-generation antipsychotics; MRI, magnetic resonance imaging; PANSS, Positive and Negative Symptom Scale; SANS, Scale for Assessment of Negative Symptoms.

Next, we standardized decision scores, termed diagnostic expression scores in the following, and tested them for group-level differences (eFigure 16 in Supplement 1), including BrainAGE as covariate (eFigures 17 in Supplement 1). We also generated a differential diagnostic classifier in NeuroMiner to separate bvFTD from patients with established AD and explored how schizophrenia, MD, or MCI/early-stage AD groups were positioned in this neuroanatomical space (eFigure 18 in Supplement 1). Then, NeuroMiner was used to assess the predictability of diagnostic expression scores in bvFTD and schizophrenia samples by applying pattern regression to available sociodemographic, clinical, and biological variables (Figure 2; eTable 3 and eFigures 19 to 21 in Supplement 1).

After completing this analysis phase, we evaluated the prognostic value of diagnostic classifiers and BrainAGE by applying them to young patients with CHR (n = 160) or ROD (n = 161) drawn from the Personalised Prognostic Tools for Early Psychosis Management (PRONIA) study.^9,44^ Linear mixed models were used to analyze 2-year functional trajectories in high vs low pattern expression groups (eFigure 22 and eTable 7 in Supplement 1), while accounting for BrainAGE effects (eTable 8 in Supplement 1). Genetic separability of pattern expression groups was explored using NeuroMiner based on polygenic risk scores (PRS) for FTD, AD, and schizophrenia (eFigure 23 in Supplement 1).

**Figure 3.**
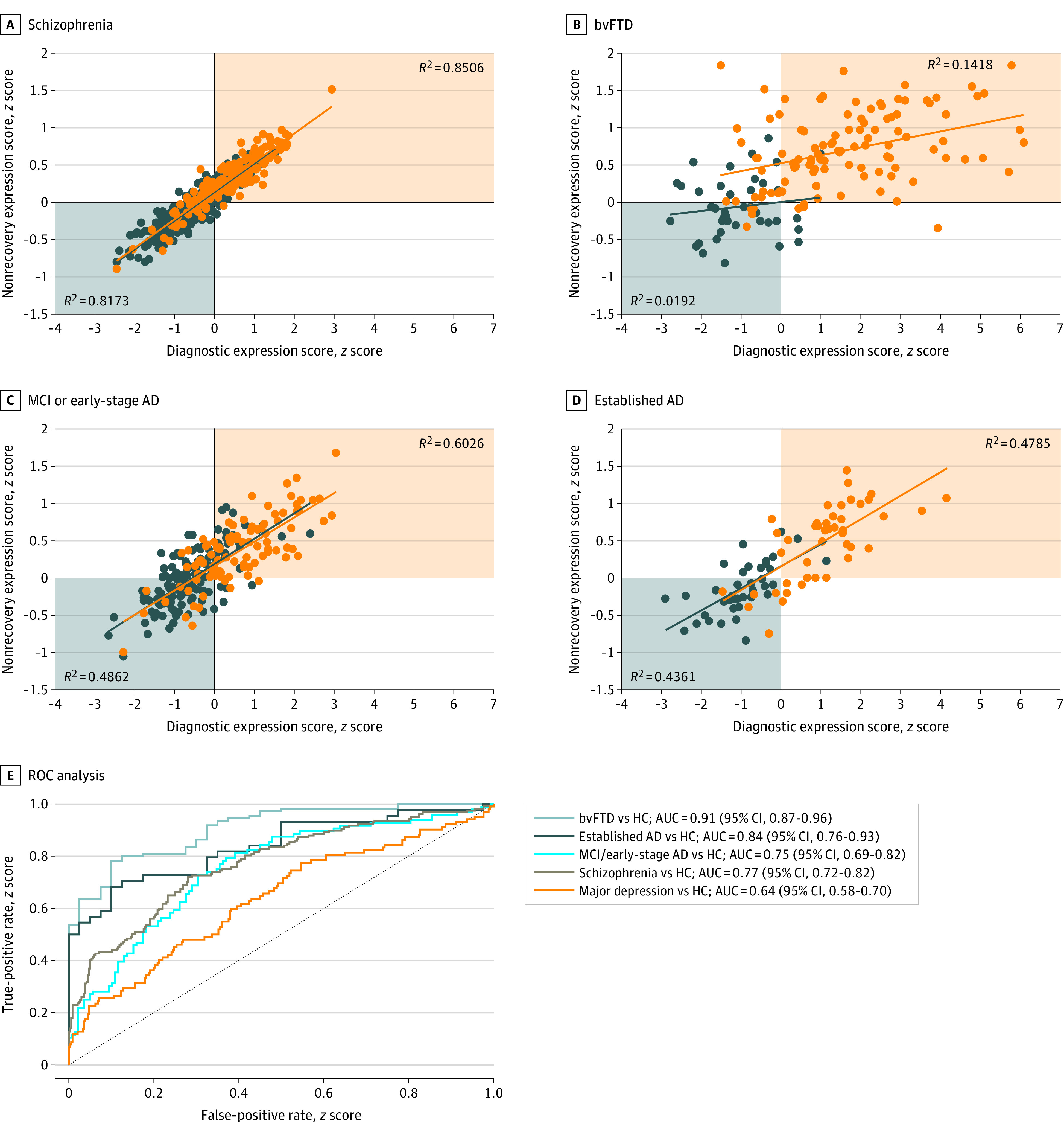
Associations of the Nonrecovery Prediction Model and the 4 Diagnostic Classifiers Orange indicates the patient group; blue, the healthy control group. Results were obtained after applying the nonrecovery classifier to patients with behavioral-variant frontotemporal dementia (bvFTD), established Alzheimer disease (AD), mild cognitive impairment (MCI) or early-stage AD, and schizophrenia and the respective healthy control (HC) samples. Scatterplots (A-D) describe the associations between the nonrecovery classifier and the respective diagnostic classifier’s diagnostic expression score in the given derivation cohort. E, The receiver operating characteristic curve analysis displays the separability of patients and HCs in the given diagnostic sample based on the prognostic score produced by the PRONIA nonrecovery classifier for the given sample. See eTable 10 in Supplement 1 for a tabular representation of the nonrecovery classifier prognostic performance and eFigures 24 to 26 in Supplement 1 for the visualization, topographical comparison, and spatial specificity test of the prognostic signature. AUC indicates area under the receiver operating characteristic curve.

**Figure 4.**
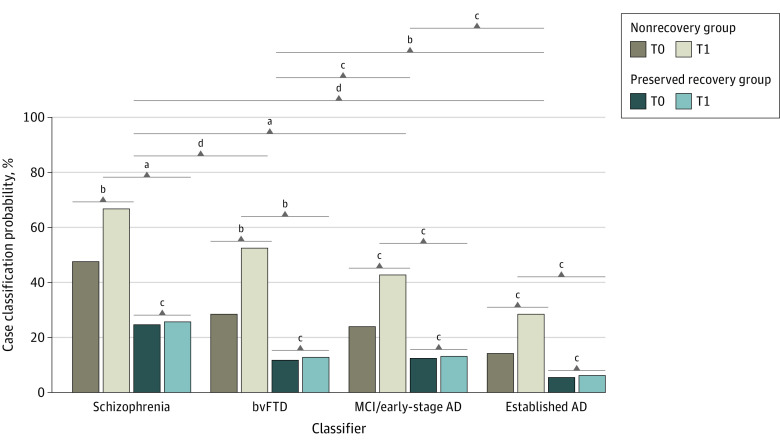
PRONIA Longitudinal Magnetic Resonance Imaging Analysis Describing the Development of Case Classification Likelihoods Between the Baseline and Follow-up Magnetic Resonance Imaging Data of the PRONIA Nonrecovery and Recovery Samples Likelihood changes over time were compared by means of generalized estimating equations including Brain Age Gap Estimation as a covariate. Results of estimated marginal means analyses conducted for the functional trajectory, time point, and classifier factors were visualized. See eTable 11 in Supplement 1 for a tabular representation of results. AD indicates Alzheimer disease; bvFTD, behavioral-variant frontotemporal dementia; MCI, mild cognitive impairment; T0, baseline visit; T1, 1-year follow-up visit. ^a^*P* < .01. ^b^*P* < .05. ^c^Not significant. ^d^*P* < .001.

Next, we investigated the presence of a life span, transdiagnostic signature of poor disease outcome by training a prognostic classifier in NeuroMiner to separate nonrecovery from preserved recovery courses in patients with CHR or ROD (eFigures 24 to 26 in Supplement 1). We applied this classifier to the case-control samples to test associations with the previously produced diagnostic expression scores (Figure 3A), while controlling for BrainAGE effects (eTable 9 in Supplement 1). Additionally, the nonrecovery classifier underwent sensitivity analyses at more lenient nonrecovery definitions (eTable 10 in Supplement 1) and was diagnostically validated in case-control samples using receiver operating characteristic curve analyses (Figure 3B). Third, we evaluated whether the diagnostic expression scores generated for PRONIA patients could be used as features for an alternative nonrecovery predictor (eFigure 27 in Supplement 1). Fourth, we analyzed stratification effects of all diagnostic or prognostic classifiers on long-term cognitive decline in patients with MCI or early-stage AD (eFigure 28 and eTable 11 in Supplement 1). Finally, in patients with CHR or ROD with longitudinal MRI data (n = 216), 1-year diagnostic pattern progression was compared in patients with nonrecovery vs preserved recovery (Figure 4; eTable 12 in Supplement 1).

## Results

### Group-Level Descriptive Analysis Results

Of 1870 included patients, 902 (48.2%) were female, and the mean (SD) age was 38.0 (19.3) years. Severe cognitive impairment (Clinical Dementia Rating [CDR] score greater than 3) was observed in patients with bvFTD (mean [SD] CDR, 5.6 [3.5]) and established AD (mean [SD] CDR, 5.5 [3.0]), different from patients with MCI or early-stage AD (mean [SD] CDR, 0.7 [2.7]) (eTable 1 in Supplement 1). Psychiatric symptoms differentiated bvFTD from established AD, ie, affective flattening and irritability/impulsivity. Accordingly, compared with patients with AD, patients with bvFTD more frequently received antipsychotics (antipsychotics (31 of 108 [28.7%] vs. 5 of 44 [11.4%]) and antidepressants (51 of 108 [47.2%] vs. 15 of 44 [34.1%]). The MCI/early-stage AD cohort was older (mean [SD] age, 73.3 [7.6] years; *F*_2,245_ = 40.6; *P* < .001) than patients with established AD (mean [SD] age, 66.5 [8.7] years) who had been age matched to the bvFTD cohort. Most patients with MCI or early-stage AD (65 of 96 [67.7%]) fulfilled MCI criteria (CDR, 0.5) and thus presented early disease conditions.

Patients with schizophrenia were younger than the dementia or MD samples (mean [SD] age, 30.8 [10.0] years; *F*_4,502_ = 373.3; *P* < .001). Both psychiatric samples had an illness duration of 4.5 years or more and showed moderate to severe symptoms. Most patients with schizophrenia were prescribed antipsychotics (133 of 157 [88.1%]), while only 18 of 102 patients with MD (17.6%) received these treatments (χ^2^_1_ = 125.5; *P* < .001).

CHR samples (mean [SD] age, 23.8 [5.4] years) and ROD samples (mean [SD] age, 25.8 [6.1] years) were younger than patient cohorts (*F*_6,821_ = 647.5; *P* < .001). Compared with patients with schizophrenia, they showed a mild disease severity (mean [SD] Positive and Negative Symptoms Scale: CHR, 46.1 [15.4]; ROD, 41.9 [10.8]). Both samples had moderate depression (mean [SD] Beck-Depression Inventory II^45^: CHR, 23.8 [11.0]; ROD, 24.5 [12.3]) and hence less affected than patients with MD. A total of 34 of 160 patients with CHR (21.5%) and 28 of 161 with ROD (17.5%) received antipsychotics.

### Diagnostic Classifiers and Signature Comparisons

The highest neuroanatomical case-control separability was found in patients with bvFTD (balanced accuracy [BAC], 85.6%; eTable 4 in Supplement 1) and established AD (BAC, 86.0%), followed by MCI/early-stage AD (BAC, 74.4%) and schizophrenia (BAC, 70.8%). The 4 classifiers’ signatures covered similar extents of insular, middle/superior temporal, and medial temporal lobe structures, while the bvFTD and schizophrenia patterns specifically involved large parts of the cingulate and prefrontal areas as well as the Heschl gyri. The schizophrenia signature was most extended, encompassing cerebellar hemispheres, vermis, and occipital cortices, while the bvFTD signature involved caudate nuclei and putamen (Figure 1A-D; eFigure 3A in Supplement 1). Cross-validation ratio mapping (eMethods in Supplement 1) indicated that prefrontal, orbitofrontal, and insular cortices were most altered in the bvFTD pattern, while the schizophrenia signature showed peak alterations in the medial temporal lobe, occipital and inferior temporal cortices, the cerebellum, and the anterior thalamic nuclei compared with the other signatures (eFigure 4A in Supplement 1). The schizophrenia pattern was most dissimilar to the other signatures, while the 2 AD patterns strongly overlapped (eFigures 3B and 4B in Supplement 1). Classifiers were topographically specific and not biased by confounders (eResults and eFigures 8 to 15 in Supplement 1).

### Crossover Classifier Application Results

The bvFTD model case-labeled 32 of 44 patients with established AD (72.7%), 54 of 96 with MCI or early-stage AD (56.3%), 65 of 157 with schizophrenia (41.4%), and 22 of 102 with MD (21.6%) (Table). Conversely, the schizophrenia classifier case-labeled 92 of 108 patients with bvFTD (85.5%), 31 of 44 with established AD (70.5%), 63 of 96 with MCI or early-stage AD (65.6%), and 55 of 102 with MD (53.9%). Patients with schizophrenia and patients with MD were labeled 2.3-fold and 7.3-fold, respectively, more often with bvFTD than with established AD (28 of 157 [17.8%] and 3 of 102 [2.9%]) (Table); these patients were labeled 1.4-fold and 1.8-fold more often with bvFTD than with MCI or early-stage AD (47 of 157 [29.9%] and 12 of 102 [11.8%]) (Table; Figure 1). Reflecting a gradient of increasing neuroanatomical heterogeneity, classifiers similarly case-labeled patients with established AD and bvFTD, while they disagreed in patients with schizophrenia or MD (eTable 6 in Supplement 1).

The repeated-measures analysis of variance showed within-patient effects of classifier type (*F*_2.47_ = 28.4; *P* < .001), between-patient effects of diagnostic group (*F*_4_ = 62.9; *P* < .001), and interaction effects (*F*_9.9_ = 40.0; *P* < .001) on diagnostic expression scores. In the post hoc comparisons (eFigure 16B in Supplement 1), patients with schizophrenia had higher bvFTD scores than established AD scores (mean difference [standard error]: 0.431 [0.072]; *P* < .001) or MCI/early-stage AD scores (0.265 [0.084]; *P* = .01), but their bvFTD and schizophrenia scores did not differ (−0.098 [0.069]; *P* = .64). These effects also characterized patients with MD (eFigure 16B in Supplement 1). Correcting for BrainAGE reduced scores in bvFTD and established AD compared with the other samples but did not change main or interaction effects (eFigure 17 in Supplement 1). Finally, the neuroanatomical alignment of schizophrenia and MD was confirmed by the differential-diagnostic classifier, which labeled 113 of 157 (72%) and 80 of 102 (78.5%), respectively, with bvFTD, while 65 of 96 patients with MCI/early-stage AD (67.7%) were labeled with established AD (eFigure 18 in Supplement 1).

### Diagnostic Expression Score Prediction Using Nonimaging Data

Schizophrenia score predictability (*R*^2^) in bvFTD measured 0.283 (Figure 2A) and was higher than those patients’ respective AD scores (eFigures 19 and 21 in Supplement 1). *C9orf72* carrier status (n = 11), oligoclonal banding in the cerebrospinal fluid (CSF), lower cognitive performance, younger age, and relatively reduced total CSF cell counts predicted higher schizophrenia scores (Figure 2B). Higher BrainAGE was not predicted by CSF markers or *C9orf72* but instead by lower cognitive performance, female sex, lower impulsivity, no relationship/partner, and younger age (*R*^2^ = 0.309; eFigure 19A in Supplement 1).

In patients with schizophrenia, bvFTD scores could be estimated (*R*^2^ = 0.108), while their AD scores could not (Figure 2A; eFigures 20 and 21 in Supplement 1). Higher scores were determined by higher body mass index, reduced insight, psychomotor retardation, affective disinhibition, and paranoid ideation (Figure 2B). Reduced bvFTD scores were predicted by treatment with first-generation antipsychotics, disorganized behavior, hostility, and poor rapport (Figure 2B). BrainAGE was predicted by higher body mass index and a less specific psychopathological pattern compared with the bvFTD prediction model (*R*^2^ = 0.074; eFigure 20A in Supplement 1).

### Longitudinal Effects and Genetic Separability of Diagnostic Patterns

Associations between global functioning trajectories and high vs low bvFTD or schizophrenia scores but not AD pattern expression were found in the PRONIA sample (bvFTD: *F*_1,772.9_ = 15.5; *P* < .001; schizophrenia: *F*_1,774.6_ = 13.6; *P* < .001) (eTable 7 and eFigure 22 in Supplement 1). bvFTD and schizophrenia pattern expression was associated with PRONIA study group (bvFTD: *F*_1,778.1_ = 8.9; *P* = .003; schizophrenia: *F*_1,779.1_ = 9.7; *P* = .002), with patients with CHR driving this effect. These patterns particularly stratified Functional Remission of General Schizophrenia (FROGS)^46^ Daily Life and Relationships trajectories (eTable 7 in Supplement 1), while study group effects were prominent in the Activities and Quality of Adaptation domain. Main effects of BrainAGE on global functioning were detected across all 4 classifiers, but no interactions emerged (eTable 8 in Supplement 1).

The genetic classification analysis of high vs low pattern expression groups demonstrated PRS-based separability only in the bvFTD-defined and schizophrenia-defined strata of patients with CHR (bvFTD: BAC, 65.2%; *P* = .008; schizophrenia: BAC, 67.9%, *P* = .008) (eFigure 23 in Supplement 1). Discriminative functions involved transdiagnostic PRS patterns with increased FTD, AD, and schizophrenia PRS predicting high pattern expression at genome-wide thresholds (eFigure 23C in Supplement 1).

### Prognostic-Diagnostic Pattern Validation in Recovery-Based Patient Strata

Stratification of the PRONIA sample for functional nonrecovery identified a transdiagnostic subgroup of 23 patients (10 with CHR and 13 with ROD) (eFigure 24A in Supplement 1) who did not improve over 2 years. The prognostic structural MRI classifier predicted recovery-related outcomes (BAC, 64.1%; *P* = .005) (eFigure 24B and C in Supplement 1) and showed topographic specificity (eFigure 26 in Supplement 1). An alternative prognostic model operating on age, sex, diagnostic expression scores, and BrainAGE performed with a BAC of 68.8% (eFigure 27 in Supplement 1), with bvFTD expression and BrainAGE being the most predictive features. The prognostic and diagnostic classifiers (1) overlapped in the prefrontal, cingulate, hippocampal, parahippocampal, insular, and cerebellar cortices (eFigures 24D and 25 in Supplement 1); (2) produced correlated diagnostic expression scores in schizophrenia (*R^2^* = 0.85), MCI and early-stage AD (*R^2^* = 0.60), established AD (*R^2^* = 0.48), and bvFTD (*R^2^* = 0.15) (Figure 3A; eTable 9 in Supplement 1); and (3) induced similar areas under the curve as the case-control classifiers in the respective samples (Figure 3B; eTables 4 and 10 in Supplement 1). A sensitivity analysis showed that a more lenient nonrecovery cutoff weakened prognostic-diagnostic associations (eTable 10 in Supplement 1). Finally, prognostic and diagnostic classifiers invariantly predicted 9-year cognitive decline in patients with MCI or early-stage AD (eResults, eTable 12, and eFigure 28 in Supplement 1).

### Progression of Diagnostic Patterns Over Time

We observed a 1-year progression of diagnostic patterns in patients with nonrecovery CHR and ROD. Significant effects on patients’ diagnostic assignments included recovery type (Wald χ^2^_1_ = 9.0; *P* = .003), classifier type (Wald χ^2^_3_ = 41.1; *P* < .001), and time point (Wald χ^2^_1_ = 8.8; *P* = .003) (eTable 11 in Supplement 1) and were driven by bvFTD or schizophrenia pattern progression (bvFTD. 23.8%; *P* = .02; schizophrenia, 20%; *P* = .03) compared with preserved-recovery individuals (bvFTD, 1.0%; *P* = .40; schizophrenia, 1.0%; *P* = .54) (Figure 4; eTable 11 in Supplement 1). BrainAGE showed a main effect on diagnostic assignment (Wald χ^2^_1_ = 28.1; *P* = .003), but no interactions effects.

## Discussion

The comparative machine learning analysis of the neuroanatomical patterns of bvFTD, AD, and schizophrenia revealed a frontotemporal interface between bvFTD and schizophrenia. BrainAGE correction did not alter the specificity of this interface but reduced overall group differences, in line with findings of BrainAGE increasing from affective, over psychotic to neurodegenerative disorders.^40^ Differential diagnostic classification confirmed the alignment of psychiatric cohorts with bvFTD rather than AD and an interaction between BrainAGE and bvFTD pattern expression in patients with MD, extending our previous findings.^38^ More generally, we observed a gradient of increasing neuroanatomical heterogeneity ranging from bvFTD to schizophrenia and MD due to a stronger differentiation of disease signatures in patients with schizophrenia and MD. Thus, these signatures could provide meaningful intermediate phenotypes of neurofunctional systems differentially affected by bvFTD and AD and facilitate the decomposition of neurobiological heterogeneity in psychiatric disorders.^47^

Brain patterns of schizophrenia and bvFTD were linked to sociodemographic, clinical, and biological variables available in each cohort and could be predicted with higher precision than AD patterns. This observation corroborated our hypothesis of a neuroanatomical interface between the 2 disease groups, which may be associated with shared clinical and biological dimensions; in schizophrenia, bvFTD expression was predicted by body mass index, psychomotor slowing, reduced insight, affective disinhibition, and paranoid ideation. These findings agree with previous reports of prominent prefrontal, limbic, and paralimbic volume reductions in patients with Kraepelinian manifestations, ie, courses characterized by psychosocial disability and treatment-refractory negative symptoms.^3,10,48,49^ Conversely, patients with bvFTD with high schizophrenia scores were more likely to be younger, carry the *C9orf72* variant, show oligoclonal banding without increased CSF cell count, and have more cognitive deficits. Of note, patients with *C9orf72*-mediated bvFTD typically present with earlier and more pronounced neurocognitive impairment compared with noncarriers.^33,50^

These findings may speculatively point to neuroinflammatory alterations shared by psychotic disorders and bvFTD. Recent studies revealed that the *C9orf72* variant activates proinflammatory pathways along the gut-brain axis,^31^ and thus contributes to microglia-mediated inflammation in bvFTD.^51^ Data from a recently discovered bvFTD risk-gene network further suggests that pathways involving microglia-initiated immune responses are overexpressed in prefrontal and salience systems, with a predilection for layer V neurons.^35^ Elevated neuroinflammatory markers have also been reported in schizophrenia,^52,53,54^ pointing to a dysregulation of neural development mediated by low-grade neuroinflammation.^37^ Hence, neuroinflammatory pathways may be associated with cognitive and functional impairments, potentially driving progressive brain changes in early stages of schizophrenia.^55,56^

Following these hypotheses and because of potential chronicity effects in our case-control cohorts,^57^ we applied the diagnostic classifiers to the young PRONIA patients and explored their longitudinal clinical associations. Independent of BrainAGE, we found that bvFTD and schizophrenia pattern expression at baseline, but not AD expression, were associated with reduced 2-year multidomain functioning in patients with CHR. These observations extended our initial cross-sectional findings to the time domain and to psychosis risk syndromes.

Neuroanatomical findings were supported by the genetic separability of high vs low bvFTD and schizophrenia pattern expression individuals in the CHR sample. At stricter genome-wide significance thresholds, increased PRS for FTD, AD, or schizophrenia were predictive of high pattern expression, potentially pointing to pathophysiological overlaps between neurodegenerative and psychotic disorders. These findings require independent replication and further mechanistic exploration using gene expression analysis.

Based on these findings, we explored the neuroanatomical continuum between diagnostic and prognostic disease patterns via the clinical stratification of PRONIA patients into individuals with vs without 2-year functional recovery. We found that a prognostic signature of nonrecovery was topographically aligned with diagnostic classifiers, and accordingly, it explained diagnostic expression score variation in patients with established disorders. These effects remained significant after controlling for BrainAGE, suggesting that accelerated aging^43^ only partly accounted for these correlations. These findings were supported by the fair to excellent accuracy with which the prognostic model separated cases from controls. These results were sensitive to the strictness of the nonrecovery cutoff, indicating that only PRONIA patients with the worst functional outcomes were neuroanatomically aligned with bvFTD, AD, or schizophrenia.

Furthermore, in our longitudinal MRI analysis, we found heightened bvFTD and schizophrenia expression in patients with nonrecovery, which further increased over 1 year and was independent of BrainAGE-related variation. In contrast to these specific effects in young patients, we found that all diagnostic, prognostic, and BrainAGE patterns invariably predicted faster 9-year cognitive decline in patients with MCI or early-stage AD. This global effect may point to a life span brain signature of risk for cognitive decline, potentially constituting the neuroanatomical surrogate of increased dementia risk reported across different psychiatric disorders.^5,6,25,58,59,60,61^

Our results support the reciprocity of brain signatures of early functional nonrecovery and neuropsychiatric illnesses. This reciprocity may point to a common final pathway of prefrontal and salience system disruption onto which diverse and potentially unrelated disease mechanisms converge. This final pathway may originate in the von Economo neurons (VEN), which occupy cortical layer V of the human salience system, including the anterior insula, cingulate cortex, and subcortical structures.^62^ VEN are believed to be involved in interpersonal functioning, empathy, and cognitive control.^63,64^ In bvFTD, they are affected early,^65^ with links between salience network atrophy and loss of empathy.^66^

Salience network abnormalities are well-replicated in psychiatric research^67,68^ and may progress early in psychosis.^17,18^ These alterations may moderate cognitive and functional deficits by impairing the integration of internal and task-oriented mental activity.^69^ Despite neuroimaging-based findings, histological studies of the salience network in psychiatric disorders are still rare. Two post mortem studies reported reduced density of and increased lysosomal aggregations in VEN in schizophrenia but not bipolar disorder.^70,71^ VEN abnormalities may characterize bvFTD and schizophrenia-spectrum disorders but not bipolar disorders^71^ or behavioral-variant AD.^72^ Taken together, these and our findings may point to VEN alterations across psychosis and frontotemporal dementia, which warrant careful histopathological and transcriptomic exploration in post-mortem samples.

### Limitations

Our study has several limitations. First, the follow-up interval of the PRONIA sample was shorter (2 years) compared with the OASIS-3 cohort (9 years), which may have reduced our ability to detect prognostic associations produced by AD patterns in the former sample. Second, the robustness of the nonrecovery signature might be limited because of training sample size, although its validation in the case-control cohorts speak for the opposite. Third, we cannot exclude that the potential alignment of bvFTD and psychosis-related brain patterns might have been partly confounded by overlapping treatment effects in both disease cohorts.^73^ However, we found that antipsychotic treatment predicted lower bvFTD pattern expression in schizophrenia and did not associate with schizophrenia expression in bvFTD. Finally, bvFTD encompasses different disease pathologies, producing heterogeneous cognitive-behavioral phenotypes.^34^ Because of training sample size limitations, we could not quantify how this heterogeneity influenced our results.

## Conclusions

In summary,^74^ we identified specific overlaps between macroscopic brain signatures of schizophrenia and bvFTD. These overlaps mediated aspects of the clinical-behavioral heterogeneity of both conditions, ie, a negative avolitional syndrome in the former, and a potentially neuroinflammation-associated phenotype involving neurocognitive dysfunction in the latter. We found that the presence of bvFTD and schizophrenia patterns in patients with psychosis risk syndromes predicted impaired functional recovery. Finally, our study revealed that young patients with poor functional outcomes overexpressed these brain patterns over time, in line with Kraepelin’s concept of dementia praecox as a progressive frontotemporal disorder. Further studies into molecular disease pathways are needed to clarify how different pathophysiological processes project on overlapping neural alterations in bvFTD and schizophrenia-spectrum disorders.

## Notes

Supplement 1.
eMethods.

eResults.
**eTable 1.** Description of Sociodemographic and Clinical Features of Patient Cohorts and Healthy Control (HC) Samples **eTable 2.** MR Scanner Systems and Structural MRI Sequence Parameters Used to Examine Study Participants in the Different Cohorts of the Study **eTable 3.** Variables Available in the Munich and FTLDc Studies for the Prediction of Neuroanatomical Expression Scores in the ν-SVR Analysis **eTable 4.** Classification Performance of Disease Classifiers as Measured Using Repeated Nested Cross-validation **eTable 5.** Effects of Covariate Correction on Classifier Performance in Healthy Controls **eTable 6.** Pairwise McNemar Tests Probing Classifiers for Nonequality of Patients’ Diagnostic Assignments **eTable 7.** Results of Mixed-Linear Models Investigating Group-Level Associations Between Neuroanatomical Pattern Expression and Functioning Trajectories in Patients With Clinical High-Risk (CHR) States for Psychosis or Recent-Onset Depression (ROD) **eTable 8.** Results of BrainAGE-Adjusted Mixed-Linear Models Investigating Group-Level Associations Between High vs Low Neuroanatomical Pattern Expression and Global Functioning Trajectories in Patients With CHR States or ROD **eTable 9.** Evaluation of Moderating BrainAGE Effects **eTable 10.** Sensitivity Analysis Comparing a More Lenient Definition of Nonrecovery in the PRONIA Sample With the Original Label Definition **eTable 11.** Longitudinal Analysis of Neuroanatomical Predictions in PRONIA Patients With Nonrecovery vs Recovery Trajectories Performed Using Generalized Estimating Equations **eTable 12.** Mixed-Effects Linear Model Analysis of Clinical Dementia Score Trajectories in Patients With MCI/Early-Stage AD and Healthy Controls (HC) Covering a 9-Years Follow-up Period **eFigure 1.** Schematic Representation of the Analysis Flow Implemented in the Study **eFigure 2.** Effects of the Dynamic Voxel Standardization on the Relationship Between Age and Total Gray Matter Volume Estimates **eFigure 3.** Mapping of Diagnostic Signatures to the AAL3 Atlas Based on Spatial Extent **eFigure 4.** Mapping of Diagnostic Signatures to the AAL3 Atlas Based on Peak CVR Values **eFigure 5.** Univariate Group-Level Differences Between Patients and Healthy Controls in the AAL3 Atlas **eFigure 6.** Associations Between Multivariate and Univariate Measures of Neuroanatomical Differences Between Patients and Healthy Controls **eFigure 7.** Validation of the Cohort Adjustment Strategy **eFigure 8.** Covariate Effects on Classifiers’ Decision Scores in the Different Healthy Control Samples of the Study **eFigure 9.** Post Hoc Evaluation of Interactions Between bvFTD Decision Scores and Diagnostic Criteria of bvFTD Subgrouping Patients into Possible, Probable, and Definitive Frontotemporal Lobar Degeneration (FTLD) **eFigure 10.** Post Hoc Evaluation of Established AD Decision Scores in Relation to the Known or Unknown CSF Biomarker Status in Patients With Established AD **eFigure 11.** Probing the bvFTD and Schizophrenia Classifiers’ Spatial Specificity via Atrophy Simulation **eFigure 12.** Visualization and Quantitative Analysis of the BrainAGE Model Developed in the Pooled HC Cohort and Applied to the Clinical Samples of the Study **eFigure 13.** Mapping of the BrainAGE Signature to the AAL3 Atlas Based on Spatial Extent **eFigure 14.** Univariate Voxel-Based Spatial Specificity Results Showing Covariate-Corrected Negative Associations Between Diagnostic Expression Scores and Dynamically Standardized GMV Maps of Patients With bvFTD and Healthy Controls **eFigure 15.** Univariate Voxel-Based Spatial Specificity Results Showing Covariate-Corrected Negative Associations Between Diagnostic Expression Scores and Dynamically Standardized GMV Maps of a Pooled Sample of Patients With Schizophrenia, Major Depression, and Healthy Controls **eFigure 16.** Results of the Repeated-Measures ANOVAs **eFigure 17.** Repeated-Measures Analysis of Variance With BrainAGE as a Covariate **eFigure 18.** Differential Diagnostic Classifier Trained to Separate Between bvFTD and Established AD and Applied to Patients With Schizophrenia, Major Depression, and MCI/Early-Stage AD **eFigure 19.** Analysis of SVR Models Predicting bvFTD Patients Individual BrainAGE **eFigure 20.** Analysis of SVR Models Predicting Schizophrenia Patients Individual BrainAGE **eFigure 21.** Quade Tests Comparing the Performance of the ν-SVR Models in Predicting Diagnostic Expression Scores and BrainAGE in bvFTD Patients **eFigure 22.** Global Functioning Trajectories of the PRONIA Cohort Demonstrating Interaction Effects Between bvFTD **eFigure 23.** Machine-Learning–Based Analysis of Multivariate Polygenic Risk Signatures Informing a Possible Genetic Discrimination of High vs Low bvFTD, Schizophrenia (SCZ), Established AD, MCI/Early-Stage AD, and BrainAGE Pattern Groups in CHR or ROD Patients **eFigure 24.** The Prognostic Nonrecovery Classifier Trained on PRONIA CHR and ROD Patients **eFigure 25.** Mapping of the Prognostic Nonrecovery Classifier’s Signature to the AAL3 Atlas Based on Spatial Extent **eFigure 26.** Probing the Spatial Specificity of the Prognostic Nonrecovery Classifier by Means of Atrophy Simulation **eFigure 27.** Prognostic Classification of CHR and ROD Patients With Functional Nonrecovery vs Preserved Recovery Using the Diagnostic Scores and BrainAGE Estimates Previously Generated by the Independent Application of Respective Models to the PRONIA Baseline Data **eFigure 28.** Trajectory Analysis Covering a 9-Years Follow-up Period of Clinical Dementia Rating Scores in Patients With MCI/Early-Stage AD (Left) or Healthy Controls (Right)
eReferences.

Supplement 2. International FTD-Genetics Consortium (IFGC), the German FTLD Consortium, and the PRONIA Consortium
