# Supplementary material for: Exploring Links Between Psychosis and Frontotemporal Dementia Using Multimodal Machine Learning: Dementia Praecox Revisited
Source: JAMA Psychiatry. 2022 Aug 3;79(9):907–19. doi: 10.1001/jamapsychiatry.2022.2075 (PMC9350851; doi:10.1001/jamapsychiatry.2022.2075)
Supplement: Supplement 1. — eMethods. eResults. eTable 1. Description of Sociodemographic and Clinical Features of Patient Cohorts and Healthy Control (HC) Samples eTable 2. MR Scanner Systems and Structural MRI Sequence Parameters Used to Examine Study Participants in the Different Cohorts of the Study eTable 3. Variables Available in the Munich and FTLDc Studies for the Prediction of Neuroanatomical Expression Scores in the ν-SVR Analysis eTable 4. Classification Performance of Disease Classifiers as Measured Using Repeated Nested Cross-validation eTable 5. Effects of Covariate Correction on Classifier Performance in Healthy Controls eTable 6. Pairwise McNemar Tests Probing Classifiers for Nonequality of Patients’ Diagnostic Assignments eTable 7. Results of Mixed-Linear Models Investigating Group-Level Associations Between Neuroanatomical Pattern Expression and Functioning Trajectories in Patients With Clinical High-Risk (CHR) States for Psychosis or Recent-Onset Depression (ROD) eTable 8. Results of BrainAGE-Adjusted Mixed-Linear Models Investigating Group-Level Associations Between High vs Low Neuroanatomical Pattern Expression and Global Functioning Trajectories in Patients With CHR States or ROD eTable 9. Evaluation of Moderating BrainAGE Effects eTable 10. Sensitivity Analysis Comparing a More Lenient Definition of Nonrecovery in the PRONIA Sample With the Original Label Definition eTable 11. Longitudinal Analysis of Neuroanatomical Predictions in PRONIA Patients With Nonrecovery vs Recovery Trajectories Performed Using Generalized Estimating Equations eTable 12. Mixed-Effects Linear Model Analysis of Clinical Dementia Score Trajectories in Patients With MCI/Early-Stage AD and Healthy Controls (HC) Covering a 9-Years Follow-up Period eFigure 1. Schematic Representation of the Analysis Flow Implemented in the Study eFigure 2. Effects of the Dynamic Voxel Standardization on the Relationship Between Age and Total Gray Matter Volume Estimates eFigure 3. Mapping of Diagnostic Signatures to t [file jamapsychiatry-e222075-s001.pdf]

Supplemental Online Content

Koutsouleris N, Pantelis C, Velakoulis D, et al; International FTD-Genetics Consortium (IFGC), the German Frontotemporal Lobar Degeneration (FTLD) Consortium, and the PRONIA Consortium. Exploring links between psychosis and frontotemporal dementia using multimodal machine learning: dementia praecox revisited. *JAMA Psychiatry*. Published online August 3, 2022. doi:10.1001/jamapsychiatry.2022.2075

eMethods.
eResults.
eTable 1. Description of Sociodemographic and Clinical Features of Patient Cohorts and Healthy Control (HC) Samples
eTable 2. MR Scanner Systems and Structural MRI Sequence Parameters Used to Examine Study Participants in the Different Cohorts of the Study
eTable 3. Variables Available in the Munich and FTLDc Studies for the Prediction of Neuroanatomical Expression Scores in the v-SVR Analysis
eTable 4. Classification Performance of Disease Classifiers as Measured Using Repeated Nested Cross-validation
eTable 5. Effects of Covariate Correction on Classifier Performance in Healthy Controls
eTable 6. Pairwise McNemar Tests Probing Classifiers for Nonequality of Patients' Diagnostic Assignments
eTable 7. Results of Mixed-Linear Models Investigating Group-Level Associations Between Neuroanatomical Pattern Expression and Functioning Trajectories in Patients With Clinical High-Risk (CHR) States for Psychosis or Recent-Onset Depression (ROD)
eTable 8. Results of BrainAGE-Adjusted Mixed-Linear Models Investigating Group-Level Associations Between High vs Low Neuroanatomical Pattern Expression and Global Functioning Trajectories in Patients With CHR States or ROD
eTable 9. Evaluation of Moderating BrainAGE Effects on The Association Between Diagnostic Expression Scores and Nonrecovery Expression Scores
eTable 10. Sensitivity Analysis Comparing a More Lenient Definition of Nonrecovery in the PRONIA Sample With the Original Label Definition
eTable 11. Longitudinal Analysis of Neuroanatomical Predictions in PRONIA Patients With Nonrecovery vs Recovery Trajectories Performed Using Generalized Estimating Equations
eTable 12. Mixed-Effects Linear Model Analysis of Clinical Dementia Score Trajectories in Patients With MCI/Early-Stage AD and Healthy Controls (HC) Covering a 9-Years Follow-up Period
eFigure 1. Schematic Representation of the Analysis Flow Implemented in the Study
eFigure 2. Effects of the Dynamic Voxel Standardization on the Relationship Between Age and Total Gray Matter Volume Estimates
eFigure 3. Mapping of Diagnostic Signatures to the AAL3 Atlas Based on Spatial Extent

© 2022 Koutsouleris N et al. *JAMA Psychiatry*.

eFigure 4. Mapping of Diagnostic Signatures to the AAL3 Atlas Based on Peak CVR Values
eFigure 5. Univariate Group-Level Differences Between Patients and Healthy Controls in the AAL3 Atlas
eFigure 6. Associations Between Multivariate and Univariate Measures of Neuroanatomical Differences Between Patients and Healthy Controls
eFigure 7. Validation of the Cohort Adjustment Strategy
eFigure 8. Covariate Effects on Classifiers' Decision Scores in the Different Healthy Control Samples of the Study
eFigure 9. Post Hoc Evaluation of Interactions Between bvFTD Decision Scores and Diagnostic Criteria of bvFTD Subgrouping Patients into Possible, Probable, and Definitive Frontotemporal Lobar Degeneration (FTLD)
eFigure 10. Post Hoc Evaluation of Established AD Decision Scores in Relation to the Known or Unknown CSF Biomarker Status in Patients With Established AD
eFigure 11. Probing the bvFTD and Schizophrenia Classifiers' Spatial Specificity via Atrophy Simulation
eFigure 12. Visualization and Quantitative Analysis of the BrainAGE Model Developed in the Pooled HC Cohort and Applied to the Clinical Samples of the Study
eFigure 13. Mapping of the BrainAGE Signature to the AAL3 Atlas Based on Spatial Extent
eFigure 14. Univariate Voxel-Based Spatial Specificity Results Showing Covariate-Corrected Negative Associations Between Diagnostic Expression Scores and Dynamically Standardized GMV Maps of Patients With bvFTD and Healthy Controls
eFigure 15. Univariate Voxel-Based Spatial Specificity Results Showing Covariate-Corrected Negative Associations Between Diagnostic Expression Scores and Dynamically Standardized GMV Maps of a Pooled Sample of Patients With Schizophrenia, Major Depression, and Healthy Controls
eFigure 16. Results of the Repeated-Measures Analysis of Variance Comparing Diagnostic Expression Scores between Patient Groups.
eFigure 17. Repeated-Measures Analysis of Variance Comparing Patient Groups' Diagnostic Expression Scores With BrainAGE Included as a Covariate
eFigure 18. Differential Diagnostic Classifier Trained to Separate Between bvFTD and Established AD and Applied to Patients With Schizophrenia, Major Depression, and MCI/Early-Stage AD
eFigure 19. Analysis of SVR Models Predicting bvFTD Patients' BrainAGE and Diagnostic Expression Scores
eFigure 20. Analysis of SVR Models Predicting Schizophrenia Patients' BrainAGE and Diagnostic Expression Scores
eFigure 21. Quade Tests Comparing the Performance of the v-SVR Models in Predicting Diagnostic Expression Scores and BrainAGE in bvFTD Patients
eFigure 22. Global Functioning Trajectories of the PRONIA Cohort Demonstrating Interaction Effects with bvFTD and Schizophrenia Pattern Expression
eFigure 23. Machine-Learning–Based Analysis of Multivariate Polygenic Risk Signatures Informing a Possible Genetic Discrimination of High vs Low bvFTD, Schizophrenia (SCZ), Established AD, MCI/Early-Stage AD, and BrainAGE Pattern Groups in CHR or ROD Patients

© 2022 Koutsouleris N et al. *JAMA Psychiatry*.

eFigure 24. The Prognostic Nonrecovery Classifier Trained on PRONIA CHR and ROD Patients
eFigure 25. Mapping of the Prognostic Nonrecovery Classifier's Signature to the AAL3 Atlas Based on Spatial Extent
eFigure 26. Probing the Spatial Specificity of the Prognostic Nonrecovery Classifier by Means of Atrophy Simulation
eFigure 27. Prognostic Classification of CHR and ROD Patients With Functional Nonrecovery vs Preserved Recovery Using the Diagnostic Scores and BrainAGE Estimates Previously Generated by the Independent Application of Respective Models to the PRONIA Baseline Data
eFigure 28. Trajectory Analysis Covering a 9-Years Follow-up Period of Clinical Dementia Rating Scores in Patients With MCI/Early-Stage AD (Left) or Healthy Controls (Right)
eReferences.

This supplementary material has been provided by the authors to give readers additional information about their work.

© 2022 Koutsouleris N et al. *JAMA Psychiatry*.

eMethods.

Samples and participants

To address our hypotheses, we analyzed the data of a total of 1,870 patients and healthy controls provided by the German Frontotemporal Lobar Degeneration consortium (FTLDc, www.ftld.de), the Open Access Series of Neuroimaging Studies (OASIS-3) study (https://www.oasis-brains.org/), the Munich schizophrenia and depression cohorts,[1] and the Personalised Prognostic Tools for Early Psychosis Management (PRONIA; www.pronia.eu) study. Samples are described in detail below.

bvFTD and AD samples: Patients with bvFTD (n=108; mean [SD] age: 62.4 [9.5] years, 35.2% females; **Table 1**, main manuscript) were drawn from FTLDc, a quality-controlled, monitored multicenter initiative to register and trace patients with FTLD spectrum disorders.[2] Following international diagnostic guidelines, n=42 patients fulfilled criteria for possible bvFTD, n=52 for probable bvFTD, and n=14 for bvFTD with definitive FTLD-pathology at study inclusion.[3,4] Among latter patients, n=13 had known genetic mutations (C9orf72, n=11; progranulin, n=1; MAPT: n=2). As part of the FTLDc protocol, all patients were comprehensively assessed in annual intervals according to standard operating procedures (SOPs) which included neurological and psychiatric examinations, routine laboratory, and structural magnetic resonance imaging (sMRI). Detailed neuropsychological examinations were performed, including the German version of the Consortium to Establish a Registry of Alzheimer's Disease-Neuropsychological Assessment Battery (CERAD-NAB),[5] which contains the Mini-Mental State Examination (MMSE).[6] Dementia severity was measured using the Clinical Dementia Rating (CDR) scale. Furthermore, to assess the specificity of bvFTD-related analysis results, we extracted a sample of

© 2022 Koutsouleris N et al. *JAMA Psychiatry*.

patients with established Alzheimer's disease (n=44; mean [SD] age: 66.5 [8.7] years, 50% females), which had been recruited by FTLDc as an age-matched clinical control sample, as well as 40 healthy controls (HC; mean [SD] age: 66.4 [10.8], 47.5%). In 31 cases, the diagnosis was supported by a CSF biomarker positive status (Aβ-42 or Tau protein). In 13 cases, biomarker data was not available. HC individuals were recruited at the same FTLDc sites as the clinical samples.

We additionally analyzed 96 patients with MCI/early-stage AD (mean [SD] age: 73.3 [7.6] years, 36.5% females) and 138 HC (mean [SD] age: 71.3 [8.2] years, 39.1% females) drawn from the OASIS-3 project. This cohort is a retrospective compilation of data collected across several ongoing projects through the Washington University of Saint Louis Knight Alzheimer's Disease Research Center (ADRC) over the course of 30 years.[7] Of these patients, 65 (67.7%) met clinical criteria for Mild Cognitive Impairment (CDR=0.5), 30 (30.9%) for mild (CDR=1.0), and 1 (1.0%) for moderate dementia (CDR=2.0).

The rationale for this additional dementia control sample was three-fold: First, it extended the representation of AD to at-risk and early disease stages, thus allowing us to comprehensively model the heterogeneity of AD in order to differentiate disease stage/severity confounds from diagnosis-specific findings at the interface between bvFTD and schizophrenia. Second, we had the opportunity to gain additional certainty about the ability of our machine learning methodology to learn a disease construct across independent and heterogeneous disease representations. Third, we were able to probe the specificity of longitudinal findings obtained in the clinical high-risk group for psychosis to an analogous group at risk for AD development.

Schizophrenia and MD samples: The sample of patients with schizophrenia (n=157; mean [SD] age = 30.8 [10.0] years, 26.1% females) or MD (n=102; mean [SD] age = 42.2 [12.0] years,

© 2022 Koutsouleris N et al. *JAMA Psychiatry*.

49.0% females) has been previously described in detail.[1] In summary, patients were recruited at the Department of Psychiatry and Psychotherapy, Ludwig-Maximilian-University Munich (LMU) if they met diagnostic criteria for the respective disorders based on a consensus diagnosis of two independent psychiatrists using the Structured Clinical Interview for DSM-IV – Axis I & II Disorders. Patient ascertainment included the review of records and psychotropic medications, a semi-standardized assessment of the psychiatric and somatic history and symptom assessment using standard psychometric scales, such as the Positive and Negative Symptom Scale and the Scale for the Assessment of Negative Symptoms[8] for patients with schizophrenia and the Hamilton Depression Rating Scale[9] for patients with MD (**Table 1**, main manuscript). Exclusion criteria were (1) a history of (a) schizoaffective and/or bipolar disorder, (b) traumatic brain injury with loss of consciousness, mental retardation, anorexia nervosa, delirium, dementia, amnestic disorders, personality disorders, substance dependence, as defined by DSM-IV, (c) previous electroconvulsive treatments, and (d) somatic conditions affecting the central nervous system, as well as (2) insufficient knowledge of German, IQ < 70, and age < 18 or > 65. Samples covered both recent-onset stages (schizophrenia, n=67; depression, n=40) and relapsing stages (schizophrenia, n=90, depression, n=62) of both disorders. The MD patients' sMRI data were used to assess the specificity of findings related to the application of neurodegeneration classifiers to the schizophrenia sample. Furthermore, 335 HC (mean [SD] age = 42.3 [11.9] years, 49.0% females) were recruited as previously described, and used for sMRI image calibration (see below).[1] Written informed consent was obtained from each participant before study inclusion.

PRONIA samples: In addition to patients with neurodegenerative, schizophrenic or depressive disorders, we analyzed a sample of 321 young patients with CHR for psychosis (n=160; mean [SD] age = 23.8 [5.4] years, 51.0% females) or ROD (n=161; mean [SD] age = 25.8 [6.1] years,

© 2022 Koutsouleris N et al. *JAMA Psychiatry*.

52.8% females) recruited as part of the European PRONIA project (www.pronia.eu).[10] Furthermore, we used the structural MRI data of 529 HC from the PRONIA database to mitigate site/scanner effects in the patients' sMRI images.

PRONIA patients were followed for at least nine and up to 36 months, with three-monthly visits up to the 18-month point and 9-month visits thereafter based on the project's SOPs.[11] Here, we measured patients' functional outcomes using the GAF Symptoms and Disability scales (GAF split version),[12] as well as the Functional Remission of General Schizophrenia (FROGS) Scale,[13] which captures functioning in daily life, activities, relationships, quality of adaptation, and health and treatments. GAF and FROGS measures were available for 321 patients at the 9-month timepoint, and for 244 patients at the 18-month or later timepoints. A measure of global functional outcome was generated by standardizing the patients' baseline and follow-up GAF/FROGS scores using the baseline data and calculating a mean functioning score across the seven standardized measures for each patient at each available timepoint. Furthermore, we analyzed the data of 216 patients, who, in addition to the baseline data, had received a second sMRI scan at the 9-month timepoint, and had been examined using functional measures at the 9-month and 18-month (or later) timepoints.

Based on these data, we first assessed whether higher expression of neurodegenerative and schizophrenia patterns at *baseline* was associated with poorer functioning over time. Conversely, we then examined whether a machine learning model operating on the baseline sMRI data could correctly identify PRONIA patients with functional non-recovery, as defined by an average functional score across the 9-month and 18-month (or later) examinations ranging below the 25%-percentile of the baseline sample (**eFigure 24a**). We assessed whether the prognostic

estimates produced by this model for the bvFTD, established AD, MCI/early-stage AD, schizophrenia, or MD samples distinguished cases correctly from HC (**Figure 3B**, main manuscript). Finally, we used the serial MRI data to evaluate whether patients with functional non-recovery showed an increase of neurodegenerative and schizophrenia pattern expression between baseline and follow-up MRI scans (**Figure 4**, main manuscript).

Processing of structural MRI and genetic data

MRI data acquisition, preprocessing, and calibration across study groups

MRI data acquisition parameters are provided in **eTable 2**. All structural magnetic resonance images were processed using the Computational Anatomy Toolbox (CAT12, version 1207; http://dbm.neuro.uni-jena.de/cat12/), an extension of SPM12 (Statistical Parametric Mapping). CAT12 segmented images into grey matter (GM), white matter, and cerebrospinal fluid maps, and then high-dimensionally registered them to the stereotactic space of the Montreal Neurological Institute (MNI-152 space). The manual of the CAT12 toolbox (http://www.neuro.uni-jena.de/cat12/CAT12-Manual.pdf) details all processing steps applied to the structural images. In summary, processing steps consisted of (1) the 1st denoising step based on Spatially Adaptive Non-Local Means (SANLM) filtering; (2) an Adaptive Maximum A Posteriori (AMAP) segmentation technique, which models local variations of intensity distributions as slowly varying spatial functions and thus achieves a homogeneous segmentation across cortical and subcortical structures; (3) the 2nd denoising step using a Markov Random Field approach which incorporates spatial prior information of adjacent voxels into the segmentation estimation generated by AMAP; and (4) a Local Adaptive Segmentation (LAS) step, which adjusts the images for white matter (WM) inhomogeneities and varying gray matter (GM) intensities caused by differing iron content in e.g. cortical and subcortical structures. The LAS step is carried out before the final

© 2022 Koutsouleris N et al. *JAMA Psychiatry*.

AMAP segmentation; (5) a partial volume segmentation algorithm that is capable of modeling tissues with intensities between GM and WM, as well as GM and cerebrospinal fluid (CSF) and is applied to the AMAP-generated tissue segments; (6) high-dimensional DARTEL registration of the image to a MNI-template generated from the MRI data of 555 healthy controls in the IXI database (http://www.braindevelopment.org). The registered GM images were multiplied with the Jacobian determinants obtained during registration to produce GM volume (GMV) maps. GMV maps were resliced to 3 mm isotropic voxels before entering downstream analyses.

Two GMV maps in the FTLDc and Munich samples, and eight images from the PRONIA sample did not meet quality criteria and were excluded from subsequent analysis. The remaining images were resliced to a 3 mm isotropic voxel resolution. Finally, images were adjusted for cohort and age effects as described below.

Cohort adjustment procedure for the structural MRI data

eFigure 1 gives an overview of study's analytical framework. To enable transdiagnostic comparisons across the age ranges and projects covered by the patient samples under study, we followed a stepwise calibration strategy: First, 63 healthy control (HC) individuals matched for age and sex were selected across the FTLDc, Munich, and OASIS-3 samples (age: F=0.33, P=0.724; sex: χ^2=0; P=1). Partial correlation analysis was employed to compute cohort-level effects. The resulting beta coefficients were then applied to the entire FTLDc, Munich, and OASIS cohorts (n=1,020) to regress-out project-related differences. Second, a dynamic standardization procedure was implemented to correct each participant's GMV map for normal age-related variation: Based on an age window of ±3 years around the given participant's age, we drew a normative sample of mean (SD) N=58.6 (32.7) individuals from the cohort-corrected HC data pool (n=513). For HC individuals, we drew normative samples that did not contain the given person.

© 2022 Koutsouleris N et al. *JAMA Psychiatry*.

Voxel-level medians and standard deviations were computed for the given normative sample and were used to standardize the respective participant's GMV data.

As no HC individual in the PRONIA cohort was found to be in the age range of the FTLDc or OASIS-3 HC samples, the dynamic standardization of the PRONIA cohort was conducted separately: first, we computed site effects between HC samples by calculating the voxel-wise differences between the site-specific HC data and the global mean of the PRONIA HC individuals. This mean-centering model was applied to the entire PRONIA cohort to subtract the site-related differences from the GMV maps. The resulting site-adjusted HC pool was used for the dynamic standardization of all the mean-centered GMV maps of the PRONIA cohort, as described above.

Finally, based on the results of our previous work,[14] we included a masking procedure in all our classification analyses to further mitigate scanner-related differences between the cohorts under study. This procedure identified and removed voxels from the participants' GMV maps, which ranked below the median of the PRONIA inter-site reliability map. The reliability map was previously computed by applying generalization theory to the GMV maps of travelling healthy controls examined at all sites and scanners of the PRONIA consortium.[15]

To validate the effect of the dynamic standardization procedure, we computed global GMV measures for each study participant in the FTLDc, OASIS-3 and Munich cohorts by summing up the TIV-adjusted voxels, as well as the TIV-adjusted and dynamically standardized voxels in the respective GMV maps. Former were plotted in **eFigure 2a** and latter in **eFigure 2b** as a function of chronological age. Cubic functions were fitted to the two datasets to quantify the coefficient of determination (R^2) between global GMV measures and age before and after dynamic data calibration. We observed a reduction of R^2 from 0.66 to 0.024 through dynamic data standardization.

© 2022 Koutsouleris N et al. *JAMA Psychiatry*.

Genotyping

DNA could be extracted from the whole blood samples of 296 of 321 PRONIA patients (148 CHR, 148 ROD) who had provided blood for and consented to genetic testing. DNA was genotyped using Illumina's Infinium Global Screening (GSA) Array-24 BeadChip version 2 + Psych content (GSA). The GSA includes > 650,000 markers and offers an unparalleled genomic coverage and imputation performance. The Psych content comprises 50,000 variants associated with common psychiatric disorders such as schizophrenia, bipolar disorder, and autism spectrum disorders. After standard, stringent quality control using PLINK (e.g., sample call rate > 0.98; variant call rate > 0.98; Minor Allele Frequency > 0.01; removal of variants deviating from Hardy-Weinberg equilibrium, p < 10E-6; sex check and heterozygosity outlier analysis), a total of 505,687 variants remained in the dataset.

The post-QC genotype data were then phased with eagle v2.4.1 (https://alkesgroup.broadinstitute.org/Eagle/) and imputed with minimac 4 v1.0.2 (https://genome.sph.umich.edu/wiki/Minimac4) using 1000 genome phase 3 data as reference haplotypes panel (https://www.internationalgenome.org/home). To include reliable variants for polygenic risk score analysis we excluded imputed variants with lower imputation accuracy (i.e., R^2<0.8, n=10,962,225). Finally, we computed Polygenic Risk Scores for schizophrenia, frontotemporal dementia, and Alzheimer's Disease by means of the "clumping plus threshold" method. The PRS computation was run using PRSise v2 tool (https://www.prsice.info/) with the default parameters for clumping (i.e., R^2<0.1 considering 250kb flanking regions for each variant included in the PRS) while 10 *P*-value thresholds for variants selection were tested (i.e., 5.00e-08, 1.00e-06, 1.00e-04, 0.001, 0.01, 0.05, 0.1, 0.2, 0.5, 1.0).

© 2022 Koutsouleris N et al. *JAMA Psychiatry*.

For the computations, we employed the summary statistics for (a) schizophrenia from the most recent genome-wide association studies (GWAS) meta-analysis provided by the Psychiatric Genomic Consortium (PGC2, https://www.med.unc.edu/pgc/download-results/; n>36,989 patients and n>113,075 controls), (b) frontotemporal dementia using the GWAS meta-analysis conducted by the International Frontotemporal Dementia Genetics Consortium (https://ifgc-site.wordpress.com/data-access/; n=3526 patients and n=9402 controls), and (c) Alzheimer's Disease from the most recent GWAS meta-analysis (http://ftp.ebi.ac.uk/pub/databases/gwas/summary_statistics/GCST007001-GCST008000/GCST007511/; n=21,982 cases and n= 41,944 controls) provided by the International Genomics of Alzheimer's Project.[16] PRS were computed as the sum of the risk alleles weighed by the association estimates for the three disorders (beta of respective disorders) including all common variants (i.e., with Minor Allele Frequency > 1%) in the clumped dataset at a given *P* value threshold. PRS were standardized to have a direct comparison across sample PRS values considering variable *P* value thresholds. Moreover, since PRS values can be also influenced by population structure we extracted the first 10 Principal Components (PC) from the post QC genotype data. Pruning of genotyping data was applied prior to computing the PC to limit the effect of Linkage Disequilibrium (LD) across markers and thus to better represent the population structure in the eigenvectors. Genome-wide pruning was performed with PLINK considering window sizes of 50 variants and steps of 5 variants while a threshold of 0.5 in the R^2 correlation across paired variants was considered. The 30 PRS modelling the genetic liability for schizophrenia, frontotemporal dementia, and Alzheimer's Disease in the PRONIA patient groups were corrected for population structure effects using the partial correlation analysis conducted in the PRONIA HC sample. Specifically, beta coefficients

© 2022 Koutsouleris N et al. *JAMA Psychiatry*.

measuring the shared variance between population-structure PCs and PRS features were computed in the HC individuals and the obtained coefficients were applied to the respective patient data. Then, the adjusted patient PRS scores were standardized using the population-structure adjusted HC data and included in the machine learning analysis as described below.

Training, cross-validation, visualization, and application of machine learning models

Training and cross-validation of sMRI-based diagnostic models

First, we entered the resliced and adjusted GMV maps into our open-source machine learning software NeuroMiner (v1.05; www.proniapredictors.eu/neurominer/index.html) to train and cross-validate four diagnostic classifiers separating patients with bvFTD, established AD, MCI/early-stage AD and schizophrenia from the respective HC samples. As implemented in our previous work[10,17], the derivation and validation of diagnostic (or prognostic) classifiers was wrapped into a repeated, nested cross-validation structure[18] to exclude any possibility of information leakage between training, testing and validation data. Specifically, we used single 10-fold cross-validation at the inner cross-validation layer (CV_1) to conjointly optimize preprocessing and model training steps, and then applied the trained models to the outer validation data partition (CV_2) to generate decision scores for each participant in that partition. This derivation-validation process was repeated for the remaining 9 CV_2 training partitions until decision scores were generated for all validation participants in the CV_2 cycle. Then, participants were reshuffled within their group and the whole process was repeated 9 times, thus producing in total 10x10=100 decision scores for each study participant by only those models which had not seen the participant during the model derivation process. These 100 out-of-training (OOT) scores were concatenated into a model ensemble and the median decision score was computed for each study participant across these models.

© 2022 Koutsouleris N et al. *JAMA Psychiatry*.

Specifically, we used linear-kernel support-vector-machines (SVM) to be able to interpret the models' decision scores as distance measures between the participants' data and the optimally separating hyperplane (OSH).[19] The SVM optimizes the OSH weights so that the geometric margin between most similar study participants of opposite classes (=the support vectors) is maximized, thus making the method insensitive to outliers during the training process, and facilitating generalizability to new data during model application.[20] Importantly, this maximum-margin approach also allows to study patient cohorts with more intense or extended volumetric abnormalities compared to the training population because these abnormalities translate into higher absolute decision score values if they align with the OSH. To facilitate cross-diagnostic comparisons, we transformed median decision scores into *Z* scores by (1) computing the mean and standard deviation of the healthy controls' median decision scores in the respective disease cohort, and (2) using them to standardize the scores of all study participants in the respective cohort. Thus, standardized decision scores can be regarded as a global and inter-individually comparable measure of volumetric brain abnormalities aligning with the spatial distribution and severity of brain abnormalities encapsulated in the OSH.

Model optimization started by (1) smoothing the data with a 3, 6, or 9 mm Gaussian kernel, followed by (2) the selection of reliable voxels above an inter-site reliability cutoff of 50% as previously described,[10,11] (3) reduction of the processed GMV maps to $N_{PC} \in [15, 20, 25]$ eigenimages using Principal Component Analysis[21] (N_{PC} was informed by our previous neuroimaging work[11,14]), and (4) standardization of the resulting eigenscores using each component's median and standard deviation. Standardized eigenscores were then forwarded to linear-kernel, class-weighted SVM[22], which were trained at different regularization parameters $C \in 2^{[-6 \underset{\in\mathbb{Z}}{\rightarrow} +4]}$ to optimally separate cases from controls. Combining all free pre-processing and machine learning

parameters, model optimization was performed across 3 (smoothing) × 3 (PCA) × 11 (SVM) = 99 hyperparameter combinations. Within this hyperparameter space, the maximum average balanced accuracy [BAC=(Sensitivity+Specificity)/2] across the CV_1 test partitions identified the optimal combination, and hence the models to choose. Then, each of the 10 chosen SVM models entered a greedy forward-search wrapper to identify a parsimonious combination of eigenvariates that maximized the BAC in the given CV_1 training and CV_1 test data partitions.[10] The wrapper stopped when 50% of the features had been selected from the variable pool. Predictive features chosen by more than 50% of the CV_1 models in the given CV_2 partition were used to retrain models. Finally, the optimized models were applied to the CV_2 data, and their decision scores were combined into an ensemble-based prediction for each CV_2 validation participant.

Thus, each sMRI-based diagnostic classifier consisted of 10 CV_2 partitions * 10 CV_2 repetitions * 10 CV_1 models per CV_2 partition = 1000 SVM models. The performance of a given classifier was reported in **eTable 4** in terms of sensitivity, specificity, BAC, Positive and Negative Predictive Values (PPV/NPV), Area-Under-the-Curve (AUC), as well as Number Needed to Diagnose (NND)[23]. Furthermore, we computed the average SVM complexity across each diagnostic classifier's models, defined as $Cx = \frac{n_{SV}}{n}$, where n_{SV} is the number of support vectors in the given model and n the total number of training participants. Model complexity provides a useful metric to compare the data heterogeneity underlying a diagnostic signature, with higher values indicating higher usage of training data to derive a generalizable pattern from the training data.

Visualizing and quantitatively comparing sMRI-based disease signatures

Each classification system's diagnostic signature was visualized in terms of a cross-validation ratio (CVR) map. The CVR map describes the stability of the diagnostic signature at each feature

© 2022 Koutsouleris N et al. *JAMA Psychiatry*.

of the input data. CVR as a measure for pattern stability was inspired by the bootstrap ratio method used in the Partial Least Squares literature[24] and has been employed in our previous work for pattern visualization.[11,14] CVR maps are generated by computing the mean and standard error of all normalized SVM weight vectors (=SVM models)[25] across the entire repeated, nested cross-validation structure. Like the bootstrap ratio, the CVR of pattern element j is defined as:

$$\mathrm{CVR}_j = \frac{\left(\sum_{i=1}^{n=p_{CV_1}*k_{CV_1}*r_{CV_2}*k_{CV_2}} \hat{\mathbf{w}}_j^i\right)/n}{\sigma_{\hat{\mathbf{w}}_j^i}/\sqrt{n}}$$

Where n is the size of the SVM ensemble, p_{CV_1} is the number of CV_1 repetitions, k_{CV_1} the number of CV_1 folds, r_{CV_2} the number of CV_2 repetitions, k_{CV_2} the number of CV_2 folds, $\hat{\mathbf{w}}_j^i$ the jth element of the ith normalized weight vector $\hat{\mathbf{w}}^i = \mathbf{w}^i/\|\mathbf{w}^i\|$ in the SVM ensemble[25], $\sigma_{\hat{\mathbf{w}}_j^i}$ the standard deviation of $\hat{\mathbf{w}}_j^i$. Akin to Z scores, a CVR cutoff of ±2 can be applied to the CVR map to visualize stable pattern elements in the respective diagnostic classifier (**Figure 1**, main manuscript).

To better delineate and compare the spatial compositions of the four diagnostic classifiers, we projected their CVR maps onto the neuroanatomical atlas provided by Automated Anatomical Labeling 3 (AAL3; available at https://www.oxcns.org/aal3.html).[26] Specifically, for each classifier, we computed the percentage of voxels (K_{ROI}[%]) in each of the 170 regions-of-interest (ROI) of the atlas that scored at or below a CVR value of -2. Regions-of-interest were discarded, if we could not find any voxels meeting this stability criterion across the four diagnostic classifiers. Furthermore, we computed the most negative CVR value (CVR_{ROI}[min]) in each selected ROI, corresponding to the maximum local GMV reduction effect in the diagnostic signature separating patients from healthy controls. The K_{ROI}[%] and CVR_{ROI}[min] parcellations were visualized using spider plots (**eFigures 3a** and **4a**).

© 2022 Koutsouleris N et al. *JAMA Psychiatry*.

To compare the multivariate CVR metric to univariate statistics, we performed two-sample *t* tests in those voxels of the four diagnostic signatures that met the stability criterion of CVR≤-2. Then, the maximum T scores (T_{ROI}[max]) in each non-discarded ROI were identified by mapping each T score signature to the AAL3 atlas. The so obtained T_{ROI}[max] parcellations of the four classifiers were displayed using spider plots (**eFigure 5a**). We also used scatter plots and coefficients of determination (R^2) to measure the associations between CVR_{ROI}[min] and T_{ROI}[max] parcellations (**eFigure 6**). This analysis indicated that univariate T scores partially explained the CVR metric (R^2 range: 0.54-0.66). Finally, to quantify the similarity between the four diagnostic signatures, we computed the pairwise R^2 values between the K_{ROI} [%], CVR_{ROI}[min] and T_{ROI}[max] parcellations and plotted these values as gray-shaded matrices in **eFigures 3b**, **4b** and **5b**. This analysis showed that the K_{ROI} [%] and CVR_{ROI}[min] parcellations of our diagnostic signatures differed more strongly between each other than the respective univariate T_{ROI}[max] counterparts, except for the two AD signatures. We interpreted these differences between multivariate and univariate disease metrics in the light of the maximum-margin principle of the SVM: The algorithm focuses on those distributed and fine-grained aspects of the disease pattern that conjointly contribute to a generalizable separation of the given patient group from healthy controls. In consequence, this feature of the SVM leads to a higher degree of "spatial saliency" of the resulting diagnostic signature. We probed this assumption of spatial and predictive specificity in a set of supplementary classifier validation steps, as described below.

Computing neuroanatomical expression profiles for each study participant

To measure the presence of a given diagnostic signature across the different cohorts of our study, we applied all 1000 models of each classifier to the independent participants' standardized GMV maps. Specifically, for each participant, we computed the median decision score across these

© 2022 Koutsouleris N et al. *JAMA Psychiatry*.

1000 predictions to obtain an individualized, out-of-cross-validation (OOCV) metric for the neuroanatomical expression of the respective diagnostic signature. The OOCV-based decision scores were standardized using the healthy controls' mean and standard deviation to enable transdiagnostic comparisons as described above. Thus, at the end of this analysis, each study participant of the FTLDc, OASIS-3 and Munich cohorts was characterized by one OOT- and three OOCV-based *Z* scores. We termed these OOT- and OOCV-based *Z* scores 'diagnostic expression scores' and combined them into an individual diagnostic expression profile characterizing each study participant. These profiles were then statistically compared across diagnostic groups as described below. We also performed majority voting across the 1000 model predictions of each classifier to generate an ensemble-based label prediction for each participant.[27] Violin plots of the diagnostic expression score distributions were depicted in **Figure 1E-H**, main manuscript.

Exploring potential confounders of diagnostic classifiers

We performed several supplementary analyses to evaluate potential confounders of our diagnostic classifiers, including residual effects of cohort provenance (**eFigure 7**) and age (**eFigure 8a**), as well as potential effects of image quality ratings (IQR; **eFigure 8b**), sex (**eFigure 8c**) and total GMV (**eFigure 8d**). IQR and total GMV were estimated using the CAT12 toolbox.

First, we assessed the impact of our cohort adjustment strategy on classifiers' predictions. To this end, the four diagnostic classifiers (bvFTD vs. HC, Established AD vs. HC, MCI/early-stage AD vs. HC, Schizophrenia vs. HC) were retrained using GMV maps that had not been processed using dynamic standardization or inter-site reliability masking. The identical machine learning settings described above were employed for model training. This new set of classifiers was applied to each HC cohort in the study (FTLDc, Munich, OASIS-3, PRONIA) to produce OOT and OOCV-based decision scores. The four scores of each HC participant were averaged,

© 2022 Koutsouleris N et al. *JAMA Psychiatry*.

and the mean scores were entered into a one-way analysis of variance (ANOVA) to determine cohort-level differences caused by training classifiers without dynamic data standardization or inter-site reliability masking (**eFigure 7**). The same analysis was repeated with mean decision scores produced by the original classifiers (trained on the calibrated GMV data). The comparison of ANOVAs showed that the calibration procedure reduced the cohort-related differences from $F_{3;1039}$=173.3; P= 4.29*10^{-91} present in the non-calibrated data to $F_{3;1039}$=1.66; P=.173 in the fully calibrated images, indicating that dynamic standardization followed by inter-site reliability masking was effective in attenuating scanner- and age-related differences between study cohorts.

This finding was confirmed by the low, non-significant R^2 values computed as a measure of association between the HC individuals' age and their mean decision scores (**eFigure 8a**). However, we observed correlations between mean decision scores and IQR or total GMV (**eFigure 8b** and **8d**). We also found significant differences between male and female HC individuals, with male participants showing more patient-like mean decision scores than female participants (**eFigure 8c**). Therefore, we retrained the four diagnostic classifiers by including a covariate correction step in the preprocessing setup. Specifically, we applied partial correlation analysis to the HC data of the CV_1 training partitions to estimate the effects of sex, IQR and total GMV on the calibrated GMV maps. The resulting beta coefficients were then applied to the entire CV_1 training and test folds as well as the CV_2 validation partitions to regress-out covariate effects. **eTable 5** evaluates the predictive performance of the new OOT class predictions. The qualitative comparison of performance metrics did not demonstrate major differences in terms of sensitivity, specificity, and BAC between the two sets of diagnostic classifiers. Furthermore, we performed McNemar's tests[28] to assess the paired original-adjusted classifiers' class label predictions for

© 2022 Koutsouleris N et al. *JAMA Psychiatry*.

inequality and found no significant differences (**eTable 5**). Therefore, we decided to use the original classifiers in the downstream analysis process (see **eFigure 1**).

Next, we proceeded with specific classifier validation steps. We assessed the possibility that the early diagnostic stage of possible bvFTD (n=42) influenced the predictions of the bvFTD classifier. To this end, a post-hoc evaluation of interactions was performed between the bvFTD classifier's decision scores and diagnostic stages of bvFTD, with patients grouped into "possible", "probable", and "definitive frontotemporal lobar degeneration" (FTLD). Specifically, we conducted an ANOVA between these disease stages and HC, followed by post-hoc pairwise comparisons (**eFigure 9**). *P* values were corrected for multiple testing using Sidak's method[29] and significant effects were established at α=0.05. The same methodology was employed to assess whether Established AD patients of the FTLDc cohort differed in their neuroanatomical expression of AD (**eFigure 10**) if they had (1) a CSF biomarker-confirmed AD diagnosis (n=31) defined by a Aβ1-42 positive status (<550 pg/mL) or elevated Tau protein (>300pg/mL), or (2) no CSF biomarker data available (n=13).

Probing the diagnostic agreement between classifiers

We observed significant spatial overlaps between the diagnostic signatures of our classifiers as visualized in the pairwise R^2 matrices of **eFigures 3b** (K_{ROI}[%]) and **4b** (CVR_{ROI}[min]). This observation raised the possibility that the classifiers did not significantly differ in their case vs. control predictions. To assess this possibility, we conducted pairwise McNemar tests to probe the classifiers' diagnostic assignments for non-equality (**eTable 6**). Resulting *P* values were corrected per patient group using the False Discovery Rate (FDR) and significant classifier differences were established at α=0.05. These analyses revealed a gradient of classifier non-agreement

© 2022 Koutsouleris N et al. *JAMA Psychiatry*.

ranging from patients with Established AD (no significant differences between classifiers' predictions) to patients with schizophrenia (all pairwise non-equality comparisons P_{FDR}<.01).

Probing the spatial specificity of diagnostic classifiers

Different levels of brain atrophy present across the neurodegenerative and psychiatric disease groups of our study raised the possibility that spatially unspecific disease effects biased the training of diagnostic classifiers. Such global effects could gloss over the disease signatures during model application, thus mimicking specific disease patterns in patient groups with more pronounced global GMV reductions. For example, the schizophrenia signature was spatially more extended compared with the other disease patterns (**Figure 1a**, **eFigure 3**). This effect made the respective classifier prone to false positives if applied to bvFTD and established AD patients, who showed greater global brain atrophy compared to patients with schizophrenia (**eFigure 11a**). To test the null hypothesis of spatial non-specificity in the case of the schizophrenia and bvFTD classifiers, we performed a simulation analysis based on HC individuals pooled across the FTLDc, OASIS-3 and Munich cohorts (n=513). We rendered these participants patient-like, either (1) by adding global atrophy to their GMV maps so that the level of global atrophy (mean standardized GMV score) present in the target patient group (bvFTD, established AD, MCI/early-stage AD, schizophrenia, MD) would be matched (=null hypothesis), or (2) by matching HC and target patient groups through the computation of the mean voxel-level differences between groups and subtraction of this standardized GMV difference map from the HC group (=alternative hypothesis). All models of the bvFTD and schizophrenia classifiers were then applied to these two sets of modified GMV maps, and the obtained median decision scores were statistically compared with the scores previously calculated for the respective real patient group

© 2022 Koutsouleris N et al. *JAMA Psychiatry*.

using two-sample *t* tests. The null hypothesis of spatially unspecific prediction results was rejected at α=0.05. Findings are shown in **eFigure 11b1** and **b2** and presented in the **eResults**.

Neuroanatomical BrainAGE modeling

Because of the established evidence for increased BrainAGE (Brain Age Gap Estimation[30,31]) across neurodegenerative and psychiatric disorders,[32,33] it was critical to study the specificity of our findings with respect to this transdiagnostic marker of accelerated brain aging.[34] Specifically, we used ν-Support Vector Regression (ν-SVR)[35] to predict age from the *original* GMV maps of all HC participants included in the study, which had not undergone dynamic standardization, and hence contained the relevant age effects for predictive modelling (see **eFigure 2a**). As described above, we employed nested cross-validation to exclude any information leakage between training, test, and validation data during the model optimization process. Due to the large derivation sample size, we performed nested 5-fold cross-validation without any repetitions of the CV_1 or CV_2 cycles. The preprocessing pipeline started with scaling the target labels to the range [0, 1] and proceeded with smoothing the CV_1 training, CV_1 test and CV_2 validation cases' original GMV maps with a 3-, 6-, and 9-mm Gaussian kernel (FWHM), as described above. To adjust the data for cohort effects in this analytical scenario, we first masked the GMV maps with the inter-site reliability map as described above.

Then, within the nested cross-validation framework, we decided to use the ComBat algorithm[36,37] to attenuate cohort-related variation in the smoothed GMV data because of its ability to disambiguate covariate effects from the effects of interest (age). Of note, it was not possible to use ComBat in the training of the diagnostic classifiers because the algorithm would have modelled the effects of interest (here: patients vs. healthy controls) as a common neuroanatomical

© 2022 Koutsouleris N et al. *JAMA Psychiatry*.

pattern across cohorts. This would have potentially biased our ability to detect differences between diagnostic signatures at the initial model discovery or later cross-diagnostic model application steps. Furthermore, a known limitation of ComBat is that it cannot be readily applied to external, unseen data and therefore it would have impaired our ability to carry out the cross-diagnostic analyses within an external validation framework (**eFigure 1**).

Based on the large training sample size and informed by prior work,[31,33] the dimensionality of the adjusted training data was reduced to $N_{PC} \in [100, 250, 500]$ eigenimages by means of PCA and the eigenscores were standardized using each component's median and standard deviation. These scores were then forwarded to the linear-kernel ν-SVR algorithm to find an optimal age-predictive function among the 3 (FWHM) × 3 (PCA) = 9 preprocessing parameter combinations. We used fixed ν-SVR parameters (C=1, ν=0.1) to train predictive models, as determined by prior knowledge.[33] The adjustment, PCA transformation, and standardization parameters computed in the CV_1 training data were then invariantly applied to the CV_1 test and CV_2 validation data. The so processed test and validation data were projected into the linear kernel space, where the trained ν-SVR algorithm produced an age estimate for each case. Then, the age estimates were scaled back to the original age range of the pooled HC cohort. The rescaled age estimates were finally adjusted for the known age correlation effects using linear regression of BrainAGE (=Predicted Age – Chronological Age) against chronological age,[38] following a leave-one-out approach. The BrainAGE model's neuroanatomical signature was visualized in **eFigure 12a** using CVR mapping, as described above. BrainAGE findings are further detailed in the **eResults.** To obtain BrainAGE scores for the clinical participants the finalized BrainAGE and age bias correction models were applied without any retraining to the patients' original GMV maps. BrainAGE distributions were compared between study groups using box plots (**eFigure 12c**).

© 2022 Koutsouleris N et al. *JAMA Psychiatry*.

The BrainAGE signature was compared in relation to the diagnostic signatures by mapping the CVR map to the AAL3 atlas as described above (**eFigure 13**).

Probing the spatial specificity of diagnostic signatures at the voxel-level

As we confirmed spatial specificity of the bvFTD and schizophrenia classifiers in patient groups (see **eFigures 11b** and **c**), we now probed specificity further at the voxel-level by fitting the standardized and resliced GMV data of the bvFTD and schizophrenia cohorts with their diagnostic expression profiles. To this end, we used mass-univariate regression as provided by Statistical Parametric Mapping (SPM12, https://www.fil.ion.ucl.ac.uk/spm/software/) and entered the participants' four diagnostic expression scores as regressors of interest, as well as sex, IQR, total GMV (as computed by the CAT12 toolbox), and BrainAGE estimates (see previous section) as covariates in the design matrix. The Threshold-Free Cluster Enhancement toolbox for SPM12 (TFCE, http://www.neuro.uni-jena.de/tfce/) was employed to perform non-parametric permutation tests (5000 permutations). Contrasts were constructed to identify negative voxel-level associations between the given diagnostic expression score and standardized GMV (higher patient likeness-less GMV), while controlling for the effects of all other regressors in the design matrix. The resulting T maps were corrected for multiple comparisons using the FDR and depicted in **eFigure 14** (FTLDc cohort: bvFTD patients, HC) and **eFigure 15** (Munich cohort: patients with schizophrenia, major depression, HC). Voxel-level significance was determined at q=0.05.

Comparison of neuroanatomical expression profiles between study groups

Using SPSS (version 25, IBM Inc.), we conducted repeated-measures analyses of variances to assess differences in the expression of bvFTD, established AD, schizophrenia patterns across the bvFTD, established AD, MCI/early-stage AD, schizophrenia, and MD groups (**eFigure 16**). Sig-

© 2022 Koutsouleris N et al. *JAMA Psychiatry*.

nificant main effects were further investigated using estimated marginal means analyses of overall classifier effects (**eFigure 16a**), classifier effects within each patient group (**eFigure 16b**) and overall patient group effects (**eFigure 16c**). Significance was determined at α=0.05, corrected for multiple comparisons using Sidak's method.[29] Based on our previous findings on the associations between neuroanatomical separability of psychiatric patient cohorts and accelerated brain aging,[1,33] we repeated this analysis with BrainAGE included as covariate (**eFigure 17**).[39]

Differential diagnostic classification and neuroanatomical similarity analysis of psychiatric cohorts and patients with MCI/early-stage AD.

Then, we assessed the neuroanatomical separability between bvFTD and established AD and evaluated how the patients with schizophrenia, major depression and MCI/early-stage AD align with this different diagnostic brain space. To this end, we trained a machine learning classifier using the identical algorithmic setup as described above for the case-control analyses (**eFigure 18a** and **b**). The trained differential diagnostic classifier was then applied without any changes to the three cohorts (schizophrenia, major depression, MCI/early-stage AD). We analyzed the decision scores of these three groups alongside the OOT-based decision scores of the bvFTD and established AD patients using ANOVA. As this omnibus test was significant, pairwise post hoc comparisons were conducted. The P values obtained from these comparisons were corrected for multiple comparisons using Sidak's method and established significance at α=0.05 (**eFigure 18c**). Finally, we evaluated possible associations between BrainAGE and differential diagnostic scores in each of the five patient groups using univariate regression analyses (**eFigure 18d**).

© 2022 Koutsouleris N et al. *JAMA Psychiatry*.

Predicting diagnostic signature expression using non-imaging data

Using ν-SVR,[35] we analyzed whether bvFTD, established AD, MCI/early-stage AD or schizophrenia expression scores, as well as BrainAGE could be predicted in a subset of 127 patients with schizophrenia using baseline sociodemographic, disease course, treatment and psychometric variables, as well as BMI (**eTable 3**). A similar analysis was carried out in 81 bvFTD patients based on sociodemographic, disease course and behavioral variables, inflammatory and neurodegenerative CSF markers, as well as *C9orf72* mutation carrier status.[40,41] Predictive variables were selected based on the overlap of measured clinical constructs between cohorts, wherever possible, and formal criteria such as the degree of missing data per variable (maximum 25% of missing values per variable).

For both analyses, the same repeated nested cross-validation setup was employed as in the classification analyses (see methodological descriptions above). Specifically, we performed a feature-wise standardization and imputation of missing values using an Euclidean-distance based nearest-neighbor approach.[10,17] The training of the ν-SVR models involved finding the hyperparameter combination that maximized the R^2 between observed and predicted diagnostic expression scores within the parameter range $C \in 2^{[-6 \rightarrow 0]}$ and $\nu \in [0.05, 0.25, 0.45, 0.65, 0.85]$. We predetermined this C parameter range based on prior experience as the ν-SVR algorithm's predictive performance usually does not improve at $C \geq 1$. Furthermore, the optimization of the algorithm becomes computationally inefficient at $C \geq 1$.

As described above, the optimal-parameter model entered a greedy forward-search wrapper[10], which stopped when 50% of the features had been selected from the variable pool. Then, predictive features chosen by more than 50% of the CV_1 models in the given CV_2 partition were used to retrain models before being applied to the CV_2 cases. As in the discriminative machine

© 2022 Koutsouleris N et al. *JAMA Psychiatry*.

learning analyses, we used CVR mapping to compute the stability of the features' predictive value (**Figure 2,** main manuscript; **eFigures 19-20**). Furthermore, we repeated these analyses with BrainAGE as target label to probe the predictability and underlying feature spaces of diagnostic vs. accelerated brain aging patterns in the bvFTD and schizophrenia samples (**eFigures 19-20**). Finally, Quade test[42] was employed to compare all regression models in the bvFTD and schizophrenia samples at the omnibus level, followed by post hoc pairwise tests.[43] *P* values were corrected for multiple comparisons using the False-Discovery Rate (FDR)[44] and visualized in **eFigure 21**.

Prognostic and polygenic validation of the bvFTD, AD and schizophrenia signatures in the PRONIA cohort

Moderating group-level effects of the four neuroanatomical classifiers on functional outcomes were assessed by applying the models to the site- and age-adjusted GMV data of the PRONIA patients, thus generating diagnostic expression scores for further analyses (**eFigure 1**). To facilitate classifier comparisons, we defined patients above/below the 75%-percentile of the given expression score as belonging to the high-/low-expression sample of the respective signature. Then, for each classifier, we performed a mixed-effects linear model analysis to investigate main effects of 'neuroanatomical pattern expression' ('high' vs. 'low') and 'baseline study group assignment' (CHR vs. ROD) on the patients' global functional outcome trajectories (**eFigure 22**). The patients' recruitment sites were modeled as random effect in the analysis design. Specifically, if both main effects were significant at α=0.05, we repeated the mixed linear modeling for each of the seven functional scores (two GAF, five FROGS subscales) to explore whether specific functional domains drove effects at the global level (**eTable 7**). A correction of the α-level for multi-

© 2022 Koutsouleris N et al. *JAMA Psychiatry*.

ple testing was carried out using the FDR [44]. If both main effects in given analysis reached significance, estimated marginal means analyses were conducted to investigate whether effects were driven by CHR vs. ROD patients with high vs. low pattern expression for the given functional trajectory and classifier score. We additionally explored the effect of BrainAGE on the longitudinal analysis results by including it as covariate in the statistical design and repeating the four global functioning analyses (**eTable 8**).

Then, we explored whether the polygenic risk scores for FTD, AD and schizophrenia were associated with high vs. low pattern expression subgroups in the CHR or ROD samples (**eFigure 23**). To this end, we entered the ancestry-adjusted and standardized 30 PRS scores (3 x 10 genome-wide significance thresholds) into CHR- and ROD-specific machine learning analyses with the aim to identify multivariable genetic signatures that predicted expression groups at the single-patient level. A 10-times-repeated, nested 10-fold cross-validation was used to train and validate genetic models, following a similar algorithmic setup as described for the neuroanatomical pattern analysis. An important difference was that we decided to omit the wrapper-based feature selection previously used in the imaging domain when training now the PRS-based classifiers. This choice was informed by the high-collinearity structure of the PRS input data that consisted of PRS predictors computed at incrementally growing genome-wide significance thresholds. In contrast, the structure of the imaging based PCA space does not show any collinearity by design, thus being more suitable for a greedy forward search wrapper that filters out useful from redundant information in a binary fashion.

Optimally discriminative multi-PRS classifiers for bvFTD, schizophrenia, established AD MCI/early-stage AD, or BrainAGE-defined pattern expression groups were tested for significance using 1000 labels permutations [45], and *P* values were corrected for multiple comparisons

© 2022 Koutsouleris N et al. *JAMA Psychiatry*.

using the False-Discovery Rate. Classification results, ROC analyses and feature reliability profiles of significant models were shown in **eFigure 23**.

Testing a neuroanatomical continuum between neurodegenerative, schizophrenic and early-stage psychotic and affective disorders

Following these analyses, we conducted a separate two-step prognostic machine learning analysis to further evaluate a possible neuroanatomical continuum between PRONIA patients with functional non-recovery (**eFigure 24a**, main manuscript) and patients with bvFTD, established or MCI/early-stage AD, schizophrenia, and MD. To this end, we first analyzed the PRONIA patients' baseline GMV maps using the identical machine learning analysis setup as described above, except for the inner cross-validation cycle where four repetitions were added to increase the robustness of the training process due to a highly unbalanced outcome group distribution (n=23 non-recovery individuals in a sample of 244 patients). The obtained prognostic signature (**eFigure 24d**) was analyzed using CVR-based mapping to the AAL3 atlas as described above (**eFigure 25**) and then applied to the bvFTD, established AD, MCI/early-stage AD, schizophrenia, and MD patients, as well as the respective HC individuals to generate non-recovery decision scores for these study participants. The amount of shared variance between these scores and the respective classifiers' diagnostic decision scores was evaluated using linear regression (**Figure 3A**, main manuscript). This analysis was repeated after controlling for BrainAGE-related variation (**eTable 9**). Then we evaluated whether the prognostic classifier did not only predict functional outcomes in PRONIA, but also separated these patients from HC (**Figure 3B**, main manuscript), thus implementing a strategy of 'reverse validation' of diagnostic patterns through an independently trained prognostic model. An important step in this analysis was to probe the non-

© 2022 Koutsouleris N et al. *JAMA Psychiatry*.

recovery classifier for potential bias induced by different levels of global atrophy present in neurodegenerative and psychiatric conditions (**eFigure 26** and **Supplementary Results**). The simulation-based test has been detailed above for the topographical validation of classifiers.

In the second step, we used the PRONIA patients' age and sex, BrainAGE, and the diagnostic scores produced by the four case-control classifiers to train an alternative prognostic classifier predicting non-recovery in the PRONIA cohort. The seven features' predictive relevance was investigated using the CVR metric and depicted in **eFigure 27**. An additional sensitivity analysis of these two prognostic models was performed after defining non-recovery at a more lenient threshold (**eTable 10**).

Furthermore, we used the serial MRI data available for 216 PRONIA patients to test whether patients with functional non-recovery differed from preserved-recovery patients in terms of a progressive course of dementia or schizophrenia pattern expression. To this end, the four diagnostic classifiers were applied to the patients' follow-up scans after correcting their GMV tissue maps with the same individualized normative samples used for the respective baseline data. Additionally, we computed the patients' BrainAGE scores at the follow-up MRI examination. Generalized estimating equations with a binary logistic model were used to investigate the effects of the within-subject factors 'classifier' (schizophrenia, bvFTD, MCI/early-stage AD or established AD) and 'timepoint' (baseline vs. follow-up) as well as the between-subject factor 'recovery type' (non-recovery vs. recovery) on the predicted diagnostic class (case vs. control). The patients' BrainAGE scores measured at baseline and follow-up were added as main effect to the statistical design. Following the analysis of main and interaction effects (**eTable 11**), we conducted estimated marginal means analysis to evaluate effects per recovery type, timepoint and classifier type (**Figure 4**, main manuscript). Significance was determined at α=0.05.

© 2022 Koutsouleris N et al. *JAMA Psychiatry*.

Finally, we performed a trajectory analysis in patients with MCI/early-stage AD or healthy controls from the OASIS-3 dataset, that covered a nine-years follow-up period of Clinical Dementia Rating scores (**eFigure 28**). The goal of this supplementary analysis was to gain insight into the value of the diagnostic and non-recovery classifiers predicting long-term outcome, in analogy to the analysis conducted in the CHR and ROD patients of the PRONIA cohort. Accordingly, we assigned patients to two either high or low-scoring groups as defined by the upper quartile cutoff applied to the respective classifier's decision score distribution. The design matrix included (1) the between-subject factor 'high vs. low classifier expression' (high/low: diagnostic/prognostic score ≥75%/<75%-percentile of the respective classifiers' decision score distribution), (2) the within-subject factors 'follow-up interval' (index timepoint, 1-2 years, 2-4 years, 4-6 years, 6-9 years), 'classifier type' (MCI/early-stage AD vs. HC, bvFTD vs. HC, established AD vs. HC, schizophrenia vs. HC, PRONIA non-recovery vs. recovery classifier), and 'study group' (MCI/early-stage AD vs. HC). BrainAGE was entered as covariate in the statistical design to control for transdiagnostic accelerated aging effects. Main effects of these factors on the dependent variable Clinical Dementia Rating (CDR) score are shown in **eTable 12**. Following significant main effects of 'study group', 'high vs. low classifier expression', 'follow-up interval', and BrainAGE, we added two-way and three-way interaction contrasts to evaluate high vs. low classifier expression scores vs. BrainAGE effects with respect to the factors 'study group' and 'follow-up interval' (**eTable 12**). Statistically significant effects were determined at α=0.05.

© 2022 Koutsouleris N et al. *JAMA Psychiatry*.

eResults.

Classifier validation analyses

Classifiers were not affected by residual cohort or age-related confounds (**eFigures 7**). Potentially confounding effects of sex, IQR and total GMV were also not relevant to models' predictions (**eFigure 8, eTable 5**). Furthermore, no differences were found between possible and probable bvFTD, or between AD samples with positive or unknown CSF biomarker status in terms of the respective classifiers' decision scores (**eFigures 9-10**).

The simulation of different levels of global GMV atrophy observed between bvFTD, established AD and schizophrenia samples did not significantly explain the predictions of the bvFTD and schizophrenia classifiers in the respective patient groups (**eFigure 11**). However, the schizophrenia classifier was more biased by global atrophy due to its larger spatial extent (**Figure 1**, main manuscript; **eFigure 3**), which may have resulted in topographically non-specific predictions for the MD and MCI/early-stage AD groups (**eFigure 11b1**).

The prognostic non-recovery classifier was identically tested for bias induced by varying global atrophy levels present across case-controls samples (**eFigure 26**). Like for the diagnostic classifiers, topographically specific atrophy simulation produced decision scores for the different target patient groups which did not significantly differ from the respective observed scores. In contrast, we found that the decision scores produced by global atrophy simulation were significantly lower than observed decision scores across patient groups, except for MD (**eFigure 26**).

BrainAGE predictor and univariate topographic specificity results

The BrainAGE model predicted age with a mean average error of 5.8 years (R^2=0.86) in the CV_2 HC participants (see **Supplementary Methods**). We found high accelerated aging effects in patients with bvFTD (mean [SD]: +27.4 [16.7] years) and Established AD (+20.1 [12.8]), followed

© 2022 Koutsouleris N et al. *JAMA Psychiatry*.

by patients with schizophrenia (+9.0 [7.6]), major depression (+.3.3 [8.1]) and MCI/early-stage AD (+1.4 [8.7]; **eFigure 12**). The BrainAGE signature overlapped significantly with the bvFTD and schizophrenia patterns, in particular covering similar portions of the anterior cingulate cortex, the medial and lateral prefrontal, as well as orbitofrontal, inferior parietal, and lateral temporal cortices as well as the cerebellar regions (**eFigure 13a)**. Differences were observed in terms of a reduced spatial extent of the BrainAGE pattern compared with the bvFTD and schizophrenia signatures in the insular and medial temporal lobe structures (hippocampus, amygdala, parahippocampus), the posterior cingulate and occipital cortices, and the anterior thalamic nuclei. The overlaps between the BrainAGE, bvFTD and schizophrenia signatures were greater than those with the two AD patterns (**eFigure 13b**).

A voxel-level univariate specificity analysis was conducted because of the similarities found between diagnostic signatures, as well as between these patterns and the BrainAGE signature (**eFigures 14** and **15**). In summary, this analysis provided a fine-grained statistical mapping of the differences between diagnostic signatures described in **eFigure 3** (comparison of K_{ROI}[%]) and **eFigure 4** (comparison of CVR_{ROI}[min]). Despite significant neuroanatomical overlaps (**eFigures 3**, **4, 13**), the diagnostic signatures were also characterized by specific pattern components: (1) the schizophrenia signature specifically encompassed the cerebellum, the medial and lateral occipital cortices, the precuneus and posterior cingulate, as well as the medial and lateral temporal, posterior insular and parietal cortices, and the thalamus; (2) the bvFTD signature particularly involved the anterior cingulate, medial and lateral prefrontal cortices, the caudate nucleus and putamen, as well as the anterior insular cortex; finally (3) the two AD signatures occupied predominantly the medial temporal lobe structures and temporopolar cortices with extensions to the anterior cingulate, lateral prefrontal and inferior temporal cortices (**eFigures 14** and

© 2022 Koutsouleris N et al. *JAMA Psychiatry*.

15). A second result of these analyses was that specific pattern components could be detected across cohorts, i.e., the specific spatial aspects of the bvFTD, schizophrenia and AD patterns were present in patients with bvFTD, schizophrenia or major depression. We also found that diagnostic signature specificity was attenuated in bvFTD (**eFigure 14**) compared to schizophrenia and major depression (**eFigure 15**), while the BrainAGE pattern became more prominent in latter cohort.

Longitudinal effects of diagnostic patterns in MCI/early-stage AD patients.

A high expression of *any* neuroanatomical pattern (F=141.0; P<.001; classifier type: F=0.0; P=1.0) was associated with worse nine-year CDR courses (**eFigure 28**, **eTable 12**) in the OASIS-3 sample. This stratification effect increased significantly over time (F=24.1; P<.001) and was differentially expressed in patients (F=125.8; P<.001) vs. HC (F=24.0; P<.001). BrainAGE was independently associated with CDR course (F=85.5; P<.001), interacted with the follow-up period (F=5.7; P<.001) and differentiated patients from HC (F=19.9; P<.001).

© 2022 Koutsouleris N et al. *JAMA Psychiatry*.

eTable 1. Description of Sociodemographic and Clinical Features of Patient Cohorts and Healthy Control (HC) Samples

Samples and variables	Patients, No. (%)		HC	df	F/T/Z/χ^2	P_{FDR}
	**bvFTD**	**Established AD**				
**FTLDc**						
No.	108	44	40			
Age, mean (SD), y	62.4 (9.5)	66.5 (8.7)	66.4 (10.8)	191	4.2	.039
Female sex	38 (35.2)	22 (50)	19 (47.5)	2	3.7	.212
Educational years, mean (SD)	13.6 (3.1)	13.9 (3.2)	13.8 (2.9)	185	0.2	.875
Relationship status, in partnership	90 (83.5)	40 (90.9)	29 (72.5)	2	5.2	.117
Age at symptom onset, mean (SD), y	58.6 (11.3)	62.3 (9.1)	-	146	3.5	.110
Illness duration, mean (SD), y	3.9 (4.4)	4.0 (3.8)	-	146	−0.2	.827
Mini-Mental State Evaluation, mean (SD)	24.6 (5.0)	21.7 (6.1)	29.0 (0.8)	180	23.5	<.001
Clinical Dementia Rate Scale, mean (SD)	5.62 (3.49)	5.45 (3.04)	0.03 (0.12)	170	46.0	<.001
Delusions present, yes	10 (9.3)	0 (0.0)	-	1	4.4	.072
Hallucinations present, yes	5 (4.7)	1 (2.3)	-	1	0.5	.563
Affective flattening present, yes	52 (48.6)	9 (20.5)	-	1	10.3	.003
Depression present, yes	32 (29.9)	18 (40.9)	-	1	1.7	.236
Euphoria present, yes	6 (5.6)	0 (0.0)	-	1	2.6	.159
Anxiety present, yes	12 (11.2)	5 (11.4)	-	1	0.001	.979
Impulsivity present, yes	46 (43.0)	2 (4.5)	-	1	21.3	<.001
Treated with AP, yes	31 (28.7)	5 (11.4)	0 (0.0)	2	17.8	<.001
Treated with antidepressants, yes	51 (47.2)	15 (34.1)	1 (2.5)	2	25.7	<.001
	MCI/early-stage AD					
**OASIS-3**						
No.	96		138			
Mild cognitive impairment	65 (67.7)		-	-	-	-
Age, mean (SD), y	73.3 (7.6)		71.3 (8.2)	232	1.8	.078
Female sex	35 (36.5)		54 (39.1)	2	0.2	.679
Educational years, mean (SD)	15.2 (3.0)		16.0 (2.8)	207	−2.0	.065
Relationship status, in partnership	70 (80.5)		89 (73)	1	1.6	.228
Age at symptom onset, mean (SD), y	69.6 (8.3)		-	-	-	-

© 2022 Koutsouleris N et al. *JAMA Psychiatry*.

Illness duration, mean (SD), y	3.9 (2.4)		-	-	-	-
Mini-Mental State Evaluation, mean (SD)	24.7 (4.1)		29.0 (1.3)	107.6[a]	−9.9	<.001
Clinical Dementia Rate Scale, mean (SD)	0.67 (2.7)		0.00 (0.0)	95.5[a]	24.4	<.001
NPI-Q						
Mild-severe delusions, yes	7 (8)		0 (0)	1	10.1	.003
Mild-severe apathy, yes	30 (34.5)		5 (4.1)	1	33.6	<.001
Mild-severe depression, yes	30 (34.5)		8 (6.6)	1	26.3	<.001
Mild-severe euphoria, yes	6 (6.9)		0 (0.0)	1	8.6	.004
Mild-severe anxiety, yes	30 (34.5)		2 (1.7)	1	41.9	<.001
Mild-severe irritability, yes	39 (44.8)		11 (9.0)	1	35.8	<.001
GDS score, mean (SD), y	2.39 (2.1)		0.95 (1.4)	137	5.5	<.001
	Schizophrenia	MD				
**Munich**						
No.	157	102	335			
Age, mean (SD), y	30.8 (10.0)	42.2 (12.0)	33.0 (11.1)	596	36.8	<.001
Female sex	41 (26.1)	50 (49.0)	171 (51.0)	2	28.2	<.001
Schooling years, mean (SD), y	10.6 (2.1)	10.8 (1.7)	12.0 (1.5)	569	39.5	<.001
Age at symptom onset, mean (SD), y	25.5 (8.0)	36.5 (12.0)	-	254	78.0	<.001
Illness duration, mean (SD), y	4.5 (7.0)	5.8 (7.8)	-	250	−1.3	.185
PANSS, mean (SD), y						
Total score	81.8 (29.9)	-	-	-	-	-
Positive score	18.7 (8.1)	-	-	-	-	-
Negative score	21.9 (9.9)	-	-	-	-	-
General score	41.2 (16.4)	-	-	-	-	-
HDRS score, mean (SD), y	-	21.5 (9.3)	-	-	-	-
Treated with AP at MRI, yes	133 (88.1)	18 (17.6)	-	1	125.5	<.001
Treated with typical AP at MRI, yes	48 (31.8)	10 (9.8)	-	1	16.7	<.001
Treated with atypical AP at MRI, yes	100 (66.2)	9 (8.8)	-	1	81.8	<.001
Treated with antidepressants at MRI, yes	11 (7.3)	74 (72.5)	-	1	116.2	<.001
	CHR	ROD				
**PRONIA**						
No.	160	161	529			

© 2022 Koutsouleris N et al. *JAMA Psychiatry*.

Age, mean (SD), y	23.8 (5.4)	25.8 (6.1)	25.6 (6.1)	849	6.3	.005
Female	80 (51.0)	85 (52.8)	307 (58.9)	2	4.1	.166
Educational years, mean (SD)	13.6 (2.8)	14.9 (2.9)	15.8 (3.2)	836	30.2	<.001
Relationship status, in partnership	38 (24.2)	42 (26.1)	255 (48.9)	2	46.7	<.001
PANSS, mean (SD), y						
Total score	46.1 (15.4)	41.9 (10.8)	-	282.7[a]	2.8	.010
Positive score	9.6 (3.8)	7.5 (1.2)	-	189.3[a]	6.5	<.001
Negative score	11.6 (5.6)	10.7 (4.4)	-	298.8[a]	1.5	.166
General score	25.0 (8.6)	23.7 (6.9)	-	302.8[a]	1.5	.166
BDI-2 score, mean (SD), y	23.8 (11.0)	24.5 (12.3)	-	295	−0.5	.604
Treated with AP at MRI, yes	34 (21.5)	28 (17.5)	-	1	0.8	.399
Treated with antidepressants at MRI, yes	80 (50.6)	104 (65.0)	-	1	6.7	.015
Hospitalization before or at MRI, yes	61 (38.6)	92 (57.5)	-	1	11.4	.003

Abbreviations: AD, Alzheimer disease; AP, antipsychotics; BDI-2, Beck-Depression Inventory, version 2; bvFTD, behavioral-variant frontotemporal dementia; CHR, clinical high-risk states for psychosis; FDR, false-discovery rate; FTLDc, data from the German Frontotemporal Lobar Degeneration Consortium; GDS, Geriatric Depression Scale; HDRS, Hamilton Depression Rating Scale; MCI, mild cognitive impairment; MD, major depression; MRI, magnetic resonance imaging; NA, not applicable; NPI-Q, Neuropsychiatric Inventory–Questionnaire; PANSS, Positive and Negative Symptoms Scale; PRONIA, Personalised Prognostic Tools for Early Psychosis Management; ROD, recent-onset depression.
[a]Corrected if the Levene test for equality of variances was significant.

© 2022 Koutsouleris N et al. *JAMA Psychiatry*.

eTable 2. MR Scanner Systems and Structural MRI Sequence Parameters Used to Examine Study Participants in the Different Cohorts of the Study

Site	Model	Field Strength	Coil Chan-nels	Flip Angle	TR [ms]	TE [ms]	Voxel Size [mm]	FOV	Slice Number
**FTLDc**									
Ulm	SIEMENS Allegra	3T	1/12/32	8/9	2.2/2.3	2.0/2.1/ 4.4		256*256	
Erlangen	SIEMENS TrioTim	3T		9/10	1.3/2.3	3.5/3.0		240*256/ 256*256	
Göttingen	SIEMENS TrioTim	3T	12/32	9	2.3	3.0		224*256/ 232*256/ 237*237/ 240*256	
Munich-LMU / TUM	GE Signa/SIEMENS Biograph/Verio	3T	8	9/15	2.3	3.0		240*256	
Leipzig	SIEMENS Bio-graph/Tri-oTim/Verio	3T	12/32	9/10/18	1.3/1.9/ 1.9/2.3	3.0/3.5/ 4.3	1.0*1.0*1.0	240*256/ 256*256	
Rostock	SIEMENS Verio	3T		7/9	1.9/2.5	2.5/4.8		250*250/ 256*256/ 240*256	
Bonn	SIEMENS Skyra	3T		7/9	2.3/2.5	3.1/4.8		240*256/ 256*256	
Hamburg	SIEMENS Skyra	3T		9	2.5	3.6		256*256	
Homburg	SIEMENS Skyra	3T		9	2.0/2.3	3.0		240*256/ 256*256	
Tuebingen	SIEMENS Skyra	3T		10	2.3	2.9		240*256	
**OASIS-3**									
	SIEMENS TrioTim	3T	20	8	2.4	3.2	1.0*1.0*1.0	256*256	
	BioGraph PET-MR	3T	20	9	2.3	2.9	1.0*1.0*1.2	256*256	
**Munich data-base**	SIEMENS Magne-tom	1.5T	8	12	11.6	4.9	0.45*0.45* 1.5	230*230	126
**PRONIA**									
Munich	Philips Ingenia	3T	32	8	9.5	5.5	0.97*0.97* 1.0	250*250	190
Milan Niguarda	Philips Achieva In-tera	1.5T	8	12	Shortest (8.1)	Shortest (3.7)	0.93*0.93* 1.0	240*240	170
Basel	SIEMENS Verio	3T	12	8	2000	3.4	1.0*1.0*1.0	256*256	176
Cologne	Philips Achieva	3T	8	8	9.5	5.5	0.97*0.97* 1.0	250*250	190
Birmingham	Philips Achieva	3T	32	8	8.4	3.8	1.0*1.0*1.0	288*288	175
Turku	Philips Ingenuity	3T	32	7	8.1	3.7	1.0*1.0*1.0	256*256	176
Udine	Philips Achieva	3T	8	12	Shortest (8.1)	Shortest (3.7)	0.93*0.93* 1.0	240*240	170

© 2022 Koutsouleris N et al. *JAMA Psychiatry*.

eTable 3. Variables Available in the Munich and FTLDc Studies for the Prediction of Neuroanatomical Expression Scores in the ν-SVR Analysis

See **Figure 2** in the main manuscript and **eFigures 19-21**.

Variables used for neuroanatomical expression score prediction in the schizophrenia sample	Variables used for neuroanatomical expression score prediction in the bvFTD patient sample
**Sociodemographic predictors**	
Age	Age
Sex [male/female]	Sex [male/female]
Schooling years	Educational years
—	Relationship status [in partnership (yes/no)]
**Physical / Genetic predictors**	
Body-mass index [kg/m^2]	Cell count in CSF
—	Albumin in CSF [mg/dl]
—	Oligoclonal banding [yes/no]
—	Total Tau protein in CSF [pg/ml]
—	Phospho-Tau in CSF [pg/ml]
—	Aβ1-42 [pg/ml]
—	C9orf72 mutation carrier status [yes/no]
**Disease-course predictors**	
Age of disease onset	Age of initial symptoms
Illness duration [log(value)]	—
**Treatment predictors**	
Treated with antipsychotics at MRI scanning	Treated with antipsychotics at MRI scanning
Treated with antidepressants at MRI scanning	Treated with antidepressants at MRI scanning
**Cognitive status predictors**	
—	Mini-Mental-State Examination [MMSE]
—	Clinical Dementia Rating [CDR]
—	Clinical Dementia Rating, FTLD version [FTLD-CDR]
**Psychopathological predictors**	
PANSS-P1: Delusions	Delusions present [yes/no]
PANSS-P2: Conceptual disorganization	—
PANSS-P3: Hallucinations	Hallucinations present [yes/no]
PANSS-P4: Excitement	Euphoria present [yes/no]
PANSS-P5: Grandiosity	—
PANSS-P6: Suspiciousness/persecution	Irritability present [yes/no]
PANSS-P7: Hostility	—
PANSS-N1: Blunted affect	—
PANSS-N2: Emotional withdrawal	—
PANSS-N3: Poor rapport	—
PANSS-N4: Passive/apathetic social withdrawal	—
PANSS-N5: Difficulty in abstract thinking	—
PANSS-N6: Lack of spontaneity and flow of conversation	—
PANSS-N7: Stereotyped thinking	—
PANSS-G1: Somatic concern	—
PANSS-G2: Anxiety	Anxiety present [yes/no]
PANSS-G3: Guilt feelings	—
PANSS-G4: Tension	—
PANSS-G5: Mannerisms and posturing	—
PANSS-G6: Depression	Depression present [yes/no]
PANSS-G7: Motor retardation	—
PANSS-G8: Uncooperativeness	—
PANSS-G9: Unusual thought content	—

© 2022 Koutsouleris N et al. *JAMA Psychiatry*.

PANSS-G10: Disorientation	—
PANSS-G11: Poor attention	—
PANSS-G12: Lack of judgment and insight	—
PANSS-G13: Disturbance of volition	—
SANS: Unchanging Facial Expression	—
SANS: Decreased Spontaneous Movements	—
SANS: Paucity of Expressive Gestures	—
SANS: Poor Eye Contact	—
SANS: Affective Nonresponsivity	—
SANS: Lack of Vocal Inflections	—
SANS: Global Rating of Affective Flattening	Affective flattening present [yes/no]
SANS: Poverty of Speech	—
SANS: Poverty of Content of Speech	—
SANS: Blocking	—
SANS: Increased Latency of Response	—
SANS: Global Rating of Alogia	—
SANS: Grooming and Hygiene	—
SANS: Impersistence at Work or School	—
SANS: Physical Anergia	—
SANS: Global Rating of Avolition – Apathy	—
SANS: Recreational Interests and Activities	—
SANS: Sexual Interest and Activity	—
SANS: Ability to Feel Intimacy and Closeness	—
SANS: Relationships with Friends and Peers	—
SANS: Global Rating of Anhedonia-Asociality	—
SANS: Social Inattentiveness	—
SANS: Inattentiveness During Mental Status Testing	—
SANS: Global Rating of Attention	—

© 2022 Koutsouleris N et al. *JAMA Psychiatry*.

eTable 4. Classification Performance of Disease Classifiers as Measured Using Repeated Nested Cross-validation

In addition, mean model complexity (Cx) computed across the CV_1 cross-validation partitions measured the challenge for the SVM algorithm to detect an optimally separating hyperplane between the respective patient and health control samples. Higher Cx values indicate higher morphological heterogeneity requiring a higher percentage of individuals from the training sample to serve as support vectors in the definition of the optimally separating hyperplane. All trained models were tested for statistical significance (P) using 1000 label permutations as described in the Supplementary Methods.

**Classifiers**	**Mean (SD) Cx [%]**	**TP**	**TN**	**FP**	**FN**	**Sens [%]**	**Spec [%]**	**BAC [%]**	**AUC**	**FPR [%]**	**LR+**	**LR-**	**NND**	***P***
bvFTD vs. HC	41.8 (13.1)	87	36	4	21	80.6	90.0	85.3	0.93	10.0	8.1	0.2	1.4	<.001
Established AD vs. HC	46.6 (20.4)	35	37	3	9	79.5	92.5	86.0	0.94	7.5	10.6	0.2	1.4	<.001
MCI/early-stage AD vs. HC	59.0 (9.9)	67	109	29	29	69.8	79.0	74.4	0.83	21.0	3.3	0.4	2.1	<.001
Schizophrenia vs. HC	66.1 (5.4)	105	250	85	52	66.9	74.6	70.8	0.77	25.4	2.6	0.4	2.4	<.001

Abbreviations. Samples: *AD* Alzheimer's Disease, *bvFTD* Frontotemporal dementia, behavioral variant, *HC* healthy controls, *MCI* Mild Cognitive Impairment; **Performance metrics:** *Cx* model complexity measured as mean percentage of cases defined as support vectors at the parameter combination with the highest mean BAC in the CV_1 test data, *TP* number of true positives, *TN* number of true negatives, *FP* number of false positives, *FN* number of false negatives, *Sens* Sensitivity, *Spec* Specificity, *BAC* Balanced Accuracy, *AUC* Area-under-the Curve, *LR+* Positive Likelihood Ratio, *LR-* Negative Likelihood Ratio, *NND* Number Needed to Diagnose, *P* Permutation-based model significance.

© 2022 Koutsouleris N et al. *JAMA Psychiatry*.

eTable 5. Effects of Covariate Correction on Classifier Performance in Healthy Controls

Based on the covariate correlations identified between the healthy participants' mean decision scores and sex, IQR and total GMV (**eFigure 8**) the effects of these variables were further assessed in the OOT predictions of our original classifiers. Then, partial correlations were used to adjust the GMV data for these covariates as described in the Supplementary Methods. The OOT performance of the models retrained using the adjusted data was evaluated using sensitivity, specificity, and balanced accuracy. Inequality between the original and covariate-adjusted models' predictions was determined using McNemar's tests. Also, the associations between the adjusted classifiers' decision scores and the three covariates were recomputed to evaluate the effect of covariate correction.

	**Original classifiers**						**Classifiers adjusted for sex, IQR and GMV**						
**Diagnostic tasks**	**Sens [%]**	**Spec [%]**	**BAC [%]**	**T (P) [sex]**	**r (P) [IQR]**	**r (P) [GMV]**	**Sens [%]**	**Spec [%]**	**BAC [%]**	**χ^2 (P)**	**T (P) [sex]**	**r (P) [IQR]**	**r (P) [GMV]**
bvFTD vs. HC	80.6	90.0	85.3	**2.99 (.005)**	0.04 (.823)	-0.06 (.718)	76.9	90.0	83.4	1.50 (.221)	1.49 (.145)	0.16 (.325)	-0.17 (.307)
Established AD vs. HC	79.5	92.5	86.0	1.56 (.126)	-0.14 (.404)	-0.03 (.864)	81.8	95.0	88.4	0.10 (.752)	0.66 (.511)	-0.23 (.149)	-0.02 (.911)
MCI/early-stage AD vs. HC	69.8	79.0	74.4	0.00 (.974)	0.07 (.488)	**-0.22 (.009)**	72.9	81.2	77.0	0.00 (1.00)	0.40 (.694)	0.02 (.838)	-0.16 (.069)
Schizophrenia vs. HC	66.9	74.6	70.8	**6.34 (<.001)**	**0.29 (<.001)**	-0.04 (.456)	64.3	74.6	69.5	0.32 (.583)	0.60 (.548)	0.04 (.448)	-0.01 (.841)

Abbreviations. Samples: *AD* Alzheimer's Disease, *bvFTD* Frontotemporal dementia, behavioral variant, *HC* healthy controls, *MCI* Mild Cognitive Impairment; **Performance metrics:** *BAC* Balanced Accuracy, *D* Decision scores of original and covariate-adjusted classifiers, *Sens* Sensitivity, *Spec* Specificity; **Covariates:** *GMV* Global Gray Matter Volume, *IQR* Image quality rating, **Association metrics:** *P* P value, *r* Pearson correlation coefficient, R^2 coefficient of determination, *T* T value.

eTable 6. Pairwise McNemar Tests Probing Classifiers for Nonequality of Patients' Diagnostic Assignments

The class label predictions produced by the four classifiers in each of the five patient cohorts were compared for non-equality using McNemar tests for paired nominal data. *P* values were corrected sample-wise for multiple comparisons using FDR and determined significant at q<0.05. Significance indicates greater divergence of classifiers' diagnostic label prediction in the respective patient cohort.

	**Classifier comparisons [χ^2 (P_{FDR})]**					
**Patient samples**	**bvFTD vs. Established AD**	**bvFTD vs. MCI/Early-stage AD**	**bvFTD vs. Schizophrenia**	**Established AD vs. MCI/Early-stage AD**	**Established AD vs. Schizophrenia**	**MCI/Early-stage AD vs. Schizophrenia**
Established AD	0.36 (.820)	3.20 (.221)	0.00 (1.00)	0.17 (.820)	0.90 (.686)	4.17 (.221)
bvFTD	4.00 (.091)	3.27 (.106)	2.29 (.157)	0.17 (.683)	**7.56 (.018)**	**7.56 (.018)**
MCI/Early-stage AD	- (1.00)	**6.26 (.025)**	**4.27 (.047)**	**6.26 (.025)**	**4.27 (.047)**	0.38 (.540)
Major Depression	**17.05 (<.000)**	**4.50 (.034)**	**31.03 (<.000)**	**5.82 (.019)**	**50.02 (<.000)**	**41.02 (<.000)**
Schizophrenia	**33.23 (<.000)**	**9.03 (.003)**	**36.21 (<.000)**	**11.17 (.001)**	**71.31 (<.000)**	**50.77 (<.000)**

Abbreviations. Samples: *AD* Alzheimer's Disease, *bvFTD* Frontotemporal dementia, behavioral variant, *HC* healthy controls, *MCI* Mild Cognitive Impairment; **Association metrics:** χ^2 score of McNemar's test, P_{FDR} FDR-corrected *P* value.

© 2022 Koutsouleris N et al. *JAMA Psychiatry*.

eTable 7. Results of Mixed-Linear Models Investigating Group-Level Associations Between Neuroanatomical Pattern Expression and Functioning Trajectories in Patients With Clinical High-Risk (CHR) States for Psychosis or Recent-Onset Depression (ROD)

First, for each classifier score, the main effects of pattern expression (high [≥75% percentile] vs. low [≥75% percentile] expression groups) and study group (CHR vs. ROD patients) on global functioning trajectories were evaluated. See also **eFigure 22** for a graphical representation of the global functioning analysis results and **eTable 8** for BrainAGE-adjusted analysis results. If both main effects were significant, the analysis proceeded to functional subdomains as measured by the GAF split version and FROGS instruments. In addition, post hoc interaction effect analyses were carried out, investigating differential effects of pattern expression on functional trajectories per PRONIA study group.

	**Fixed main effects**										**Estimated marginal means analysis: pattern expression × study group**									
	**Pattern expression**					**Study group**					**CHR**					**ROD**				
	**Low [<75%]**	**High [≥75%]**	**df_2**	**F**	**P_{FDR}**	**CHR**	**ROD**	**df_2**	**F**	**P_{FDR}**	**Low [<75%]**	**High [≥75%]**	**df_2**	**F**	***P***	**Low [<75%]**	**High [≥75%]**	**df_2**	**F**	***P***
**bvFTD classifier**																				
Global functioning	0.54 (0.09)	0.29 (0.10)	772.9	**15.5**	**<.001**	0.33 (0.10)	0.51 (0.10)	778.1	**8.9**	**.003**	0.49 (0.10)	0.16 (0.12)	751.4	**12.6**	**<.001**	0.59 (0.10)	0.43 (0.11)	797.1	3.8	.053
GAF Symptoms	0.77 (0.11)	0.54 (0.13)	778.7	**7.5**	**.006**	0.54 (0.12)	0.77 (0.12)	778.0	**7.3**	**.007**	0.69 (0.12)	0.40 (0.15)	760.5	**5.7**	**.017**	0.85 (0.12)	0.69 (0.14)	799.8	2.0	.155
GAF Disability	0.68 (0.13)	0.45 (0.14)	784.0	**8.1**	**.005**	0.50 (0.14)	0.63 (0.14)	788.4	2.4	.122										
FROGS: Daily Life	0.38 (0.08)	0.06 (0.09)	806.3	**18.8**	**<.001**	0.16 (0.09)	0.27 (0.08)	809.2	2.3	.134										
FROGS: Activities	0.57 (0.10)	0.32 (0.12)	741.2	**11.1**	**.001**	0.34 (0.11)	0.56 (0.11)	745.6	**7.9**	**.005**	0.52 (0.11)	0.16 (0.14)	731.6	**10.3**	**.001**	0.63 (0.11)	0.48 (0.13)	752.3	2.0	.157
FROGS: Relationships	0.42 (0.09)	0.14 (0.11)	758.4	**12.8**	**<.001**	0.18 (0.10)	0.38 (0.10)	762.9	**5.9**	**.015**	0.33 (0.10)	0.04 (0.13)	750.3	**6.0**	**.014**	0.51 (0.10)	0.24 (0.13)	768.0	**6.8**	**.009**
FROGS: Qual. of adaption	0.54 (0.10)	0.33 (0.12)	734.5	**6.8**	**.009**	0.28 (0.11)	0.59 (0.11)	739.9	**14.8**	**<.001**	0.43 (0.11)	0.12 (0.14)	723.4	**6.8**	**.009**	0.64 (0.11)	0.53 (0.14)	747.4	1.0	.314
FROGS: Health and treat.	0.31 (0.10)	0.11 (0.12)	785.8	**7.3**	**.007**	0.14 (0.11)	0.28 (0.11)	789.0	3.5	.062										
**Schizophrenia classifier**																				
Global functioning	0.54 (0.10)	0.31 (0.10)	774.6	**13.6**	**<.001**	0.32 (0.05)	0.52 (0.04)	779.1	**9.7**	**.002**	0.48 (0.10)	0.16 (0.12)	754.1	**11.5**	**.001**	0.59 (0.10)	0.45 (0.11)	804.9	2.9	.087
GAF Symptoms	0.77 (0.11)	0.55 (0.13)	780.3	**7.0**	**.008**	0.56 (0.12)	0.77 (0.12)	784.9	**6.6**	**.010**	0.68 (0.12)	0.68 (0.14)	763.1	**4.1**	**.044**	0.87 (0.12)	0.68 (0.14)	806.9	2.9	.091
GAF Disability	0.69 (0.13)	0.45 (0.14)	786.1	**8.3**	**.004**	0.51 (0.14)	0.63 (0.13)	789.9	2.5	.113										
FROGS: Daily Life	0.37 (0.08)	0.09 (0.09)	807.0	**14.9**	**<.001**	0.17 (0.09)	0.29 (0.08)	810.1	2.7	.099										
FROGS: Activities	0.57 (0.10)	0.34 (0.12)	744.6	**9.6**	**.002**	0.34 (0.11)	0.57 (0.11)	749.3	**8.3**	**.004**	0.51 (0.11)	0.17 (0.14)	733.0	**8.9**	**.003**	0.63 (0.11)	0.50 (0.13)	765.3	1.7	.196
FROGS: Relationships	0.43 (0.09)	0.13 (0.11)	759.8	**14.6**	**<.001**	0.15 (0.10)	0.40 (0.10)	764.4	**9.9**	**.002**	0.35 (0.10)	-0.04 (0.13)	749.9	**10.9**	**.001**	0.50 (0.10)	0.30 (0.12)	778.2	**4.0**	**.045**
FROGS: Qual. of adaption	0.54 (0.10)	0.33 (0.12)	738.7	**7.1**	**.008**	0.26 (0.11)	0.60 (0.11)	744.0	**17.5**	**<.001**	0.44 (0.11)	0.09 (0.14)	725.7	**8.6**	**.004**	0.64 (0.11)	0.56 (0.13)	761.6	0.5	.483
FROGS: Health and treat.	0.31 (0.10)	0.09 (0.11)	789.1	**8.7**	**.003**	0.10 (0.11)	0.30 (0.11)	792.8	**7.4**	**.007**	0.31 (0.11)	-0.12 (0.14)	782.7	**13.8**	**<.001**	0.32 (0.11)	0.29 (0.13)	801.4	0.1	.802
**Established AD classifier**																				
Global functioning	0.48 (0.09)	0.50 (0.10)	784.5	0.1	.777	0.44 (0.10)	0.54 (0.10)	779.9	2.6	.107										
**MCI/early-stage AD classifier**																				
Global functioning	0.51 (0.09)	0.42 (0.10)	788.0	2.1	.148	0.38 (0.10)	0.54 (0.10)	789.1	**6.4**	**.011**										

Abbreviations: *FROGS* Functional Remission Of General Schizophrenia, *GAF* Global Assessment of Functioning scale, *Qual.* Quality, *Treat.* Treatment

© 2022 Koutsouleris N et al. *JAMA Psychiatry*.

eTable 8. Results of BrainAGE-Adjusted Mixed-Linear Models Investigating Group-Level Associations Between High vs Low Neuroanatomical Pattern Expression and Global Functioning Trajectories in Patients With CHR States or ROD

eTable 8.1. The association between low. vs. high bvFTD and schizophrenia expression scores and functioning trajectories was reduced by the inclusion of BrainAGE as covariate in the statistical design, but main effects remained significant. As in the main analysis, the stratification effects of both neuroanatomical patterns were primarily present in the CHR group. The main effect of BrainAGE on global functioning was significant across all four classifiers but particularly present in the established AD and MCI/early-stage AD classifiers.

	Main effects					Estimated marginal means analysis: pattern expression × study group									
						CHR					ROD				
	Group 1	Group 2	df_2	F	*P*	Low [<75%]	High [≥75%]	df_2	F	*P*	Low [<75%]	High [≥75%]	df_2	F	*P*
**bvFTD classifier**															
High vs. low expression score	0.52 (0.09)	0.36 (0.10)	813.5	**5.0**	**.025**	0.47 (0.09)	0.23 (0.12)	**776.1**	**6.3**	**.013**	0.56 (0.10)	0.49 (0.11)	818.9	0.6	.424
Study group	0.35 (0.10)	0.53 (0.10)	779.9	**7.6**	**.006**										
BrainAGE			849.9	**7.1**	**.008**										
**Schizophrenia classifier**															
High vs. low expression score	0.51 (0.09)	0.37 (0.10)	809.1	**4.3**	**.038**	0.47 (0.09)	0.24 (0.12)	**778.5**	**6.3**	**.024**	0.56 (0.10)	0.50 (0.11)	818.6	0.5	.472
Study group	0.36 (0.10)	0.53 (0.09)	782.8	**7.5**	**.006**										
BrainAGE			851.3	**8.4**	**.004**										
**Established AD classifier**															
High vs. low expression score	0.46 (0.09)	0.56 (0.10)	796.7	2.5	.120										
Study group	0.47 (0.09)	0.55 (0.09)	780.8	1.5	.229										
BrainAGE			850.1	**21.2**	**<.001**										
**MCI/early-stage AD classifier**															
High vs. low expression score	0.48 (0.09)	0.47 (0.10)	802.4	0.04	.849										
Study group	0.40 (0.09)	0.55 (0.09)	789.7	**5.3**	**.021**										
BrainAGE			848.8	**16.2**	**<.001**										

eTable 8.2. We also tested BrainAGE interactions in our analyses but did not find any significant effects, suggesting a global and transdiagnostic influence of BrainAGE on functioning.

	df_2	F	*P*
**bvFTD classifier**			
BrainAGE × Study group	505.5	0.92	.399
BrainAGE × Visit	785.0	0.06	.801
BrainAGE × Study group × Visit	505.2	0.02	.977
**Schizophrenia classifier**			
BrainAGE × Study group	506.7	0.12	.887
BrainAGE × Visit	789.9	0.05	.821
BrainAGE × Study group × Visit	506.5	0.80	.450
**Established AD classifier**			
BrainAGE × Study group	499.9	0.85	.429
BrainAGE × Visit	777.3	1.62	.203
BrainAGE × Study group × Visit	499.8	0.53	.592
**MCI/early-stage AD classifier**			
BrainAGE × Study group	499.0	0.45	.638
BrainAGE × Visit	773.9	0.26	.608
BrainAGE × Study group × Visit	498.9	0.43	.653

© 2022 Koutsouleris N et al. *JAMA Psychiatry*.

eTable 9. Evaluation of Moderating BrainAGE Effects on The Association Between Diagnostic Expression Scores and Nonrecovery Expression Scores

Evaluation of moderating BrainAGE effects on the correlations observed between non-recovery scores and diagnostic scores in patients with bvFTD, established AD, MCI/early-stage AD and schizophrenia**.** This table supplements **Figure 3** of the main manuscript. The OOT-based diagnostic scores produced by the four classifiers for the patients in the respective derivation cohorts were correlated with the scores generated by the prognostic model (**a**). Further correlation analyses assessed the variance of prognostic and diagnostic scores explained by BrainAGE (**b** and **c**). Finally, analysis (a) was repeated after residualizing prognostic and diagnostic scores for BrainAGE using partial correlation analysis. While all correlations remained significant after controlling for BrainAGE, the mediating effects of the accelerated aging marker were more pronounced in the two AD samples, reducing explained variance by 20.8% in these samples. In contrast, the explained variance dropped on average by 6.9% in bvFTD and schizophrenia patients after controlling for BrainAGE effects.

	**a)** Correlation analysis: [non-recovery vs. diagnostic scores]		**b)** Correlation analysis: [non-recovery score vs. BrainAGE]		**c)** Correlation analysis: [diagnostic score vs. BrainAGE]		**d)** Partial correlation analysis of non-recovery vs. diagnostic scores, BrainAGE as control variable	
**Sample**	R^2	***P***	R^2	***P***	R^2	***P***	R^2	***P***
bvFTD	0.142	<.001	0.067	.007	0.787	<.001	0.108	.001
Established AD	0.478	<.001	0.541	<.001	0.418	<.001	0.175	.005
MCI/early-stage AD	0.603	<.001	0.394	<.001	0.220	<.001	0.490	<.001
Schizophrenia	0.851	<.001	0.425	<.001	0.426	<.001	0.748	<.001

© 2022 Koutsouleris N et al. *JAMA Psychiatry*.

eTable 10. Sensitivity Analysis Comparing a More Lenient Definition of Nonrecovery in the PRONIA Sample With the Original Label Definition

In this supplementary analysis, non-recovery was defined as having an average global functioning during follow-up of equal or below the median of the baseline distribution of global functioning, while in the original analyses the cutoff was the lower quartile of the distribution. Two evaluate the effect of this more lenient definition of non-recovery on the neuroanatomical continuum between the signatures of the diagnostic classifiers and non-recovery signature produced by analyzing the PRONIA patients' GMV maps. Two analysis steps were performed:

eTable 10.1. First, we repeated training and cross-validation of the PRONIA non-recovery classifier using the patients' GMV maps and the identical parameter setup as described in the main analysis. The trained classifier was then applied to the dementia, schizophrenia, and major depression samples to evaluate its performance in discriminating between cases and controls. Bold-text rows list the cross-validated performance results, while the remaining rows contain the results obtained by applying the respective prognostic classifier to the external dementia, schizophrenia, and major depression samples.

Classifiers	Mean (SD) Cx [%]	TP	TN	FP	FN	Sens [%]	Spec [%]	BAC [%]	AUC	FPR [%]	LR+	LR-	NND
**Model trained with original non-recovery labels defined at lower quartile cutoff of baseline global functioning.**	**78.2**	**17**	**120**	**101**	**6**	**73.9**	**54.3**	**64.1**	**0.67**	**45.7**	**1.6**	**0.5**	**3.5**
**Model used to classify:**													
bvFTD vs. HC		97	27	13	11	89.8	67.5	78.7	0.91	32.5	2.8	0.2	1.8
Established AD vs. HC		34	27	13	10	77.3	67.5	72.4	0.84	32.5	2.4	0.3	2.2
MCI/early-stage AD vs. HC		81	75	63	15	84.4	54.3	69.4	0.75	45.7	1.8	0.3	2.6
Schizophrenia vs. HC		130	177	158	27	82.8	52.8	67.8	0.77	47.2	1.8	0.3	2.8
Major depression vs. HC		68	177	158	34	66.7	52.8	59.8	0.64	47.2	1.4	0.6	5.1
**Model trained with new non-recovery labels defined at median cutoff of baseline global functioning.**	**86.1**	**28**	**88**	**105**	**23**	**54.9**	**45.6**	**50.2**	**0.53**	**54.4**	**1.0**	**1.0**	**200.9**
**Model used to classify:**													
bvFTD vs. HC		85	16	24	23	78.7	40.0	59.4	0.65	60.0	1.3	0.5	5.4
Established AD vs. HC		39	16	24	5	88.6	40.0	64.3	0.74	60.0	1.5	0.3	3.5
MCI/early-stage AD vs. HC		80	55	83	15	83.3	39.9	61.6	0.62	60.1	1.4	0.4	4.3
Schizophrenia vs. HC		130	109	226	27	83.8	32.5	57.7	0.64	57.7	1.2	0.5	6.5
Major depression vs. HC		74	109	226	28	72.6	32.5	52.5	0.58	67.5	1.1	0.8	19.7

eTable 10.2. Second, to assess the neuroanatomical continuity between the diagnostic cohorts and the non-recovery patients in PRONIA, we used the PRONIA patients' diagnostic scores as produced by the bvFTD, established AD, MCI/early-stage AD, and schizophrenia classifiers, as well as BrainAGE scores, age, and sex to train and cross-validate an alternative non-recovery prediction model (see also **eFigure 27**). We also compared original and more lenient non-recovery labels as we did for the model trained directly on the patients' GMV maps:

Classifiers	Mean (SD) Cx [%]	TP	TN	FP	FN	Sens [%]	Spec [%]	BAC [%]	AUC	FPR [%]	LR+	LR-	NND
Original non-recovery labels	89.1	18	131	90	5	78.3	59.3	68.8	0.65	40.7	1.9	0.4	2.7
New non-recovery labels	95.1	22	122	71	29	43.1	63.2	53.2	0.58	36.8	1.2	0.9	15.7

Performance metrics: *Cx* model complexity measured as mean percentage of cases defined as support vectors at the parameter combination with the highest mean BAC in the CV_1 test data, *TP* number of true positives, *TN* number of true negatives, *FP* number of false positives, *FN* number of false negatives, *Sens* Sensitivity, *Spec* Specificity, *BAC* Balanced Accuracy, *AUC* Area-under-the Curve, *LR+* Positive Likelihood Ratio, *LR-* Negative Likelihood Ratio, *NND* Number Needed to Diagnose.

© 2022 Koutsouleris N et al. *JAMA Psychiatry*.

eTable 11. Longitudinal Analysis of Neuroanatomical Predictions in PRONIA Patients With Nonrecovery vs Recovery Trajectories Performed Using Generalized Estimating Equations

The statistical design included the within-subject factors 'timepoint' (baseline vs. one-year follow-up) and 'classifier type' (schizophrenia, bvFTD, MCI/early-stage AD, established AD vs. HC, respectively), as well as the between-subject factors 'recovery type' (non-recovery vs. preserved recovery) and 'PRONIA recruitment site'. BrainAGE was entered as covariate in the design matrix to control for potential effects of this transdiagnostic marker of accelerated aging. Main and interaction effects of these factors on the dependent binary variable 'diagnostic prediction' (case vs. control) were computed using a binomial distribution model with a probit link function. Following significant main effects of 'timepoint', 'recovery type', and 'classifier', we conducted estimated marginal means (EMM) analyses to compare classifiers (EMM 1), evaluate 'classifier type'-specific effects in patients with non-recovery vs. recovery trajectories (EMM 2) and interactions between 'classifier type', 'recovery type' and 'timepoint' (EMM 3). P values were corrected for multiple comparisons using Sidak's method. In summary, we observed a significant increase of diagnostic case predictions between baseline and follow-up MRI scans in the non-recovery vs. the recovery sample. This effect was produced by the bvFTD and schizophrenia classifiers which labeled more PRONIA non-recovery participants as bvFTD or schizophrenia patients based on their follow-up MRI compared to their baseline scan. This effect was not observed in the MCI/early-stage AD or established AD models. Furthermore, we did not observe independent 'recovery type', or 'recovery type' × 'timepoint' interactions of BrainAGE, suggesting a global effect of this covariate on PRONIA patients' neuroanatomical caseness likelihood. See **Figure 4** for a visual representation of the analysis.

**Main and interaction effects**		**df**	**Wald χ^2**	***P***
Timepoint		1	**8.8**	**.003**
Recovery type		1	**9.0**	**.003**
Classifier type		3	**41.1**	**<.001**
BrainAGE		1	**28.1**	**<.001**
PRONIA recruitment site		6	5.5	.482
Recovery type × BrainAGE		1	2.1	.147
Recovery type × Timepoint x BrainAGE		2	3.3	.190
**EMM 1: Classifier type**	**Mean (SEM) difference: pair-wise classifiers**	3	**32.7**	**<.001**
Schizophrenia vs. bvFTD classifier	0.18 (0.04)	1		**<.001**
Schizophrenia vs. MCI/early-stage AD classifier	0.20 (0.06)	1		**.002**
Schizophrenia vs. Established AD classifier	0.29 (0.06)	1		**<.001**
bvFTD vs. MCI/early-stage AD classifier	0.02 (0.04)	1		.998
bvFTD vs. Established AD classifier	0.11 (0.04)	1		**.020**
MCI/early-stage AD vs. Established AD classifier	0.09 (0.04)	1		.068
**EMM 2: Classifier type → Recovery type**	**Mean (SEM) difference: poor vs. good recovery types**			
Schizophrenia classifier	0.30 (0.11)	1	7.1	**.008**
bvFTD classifier	0.24 (0.10)	1	5.6	**.018**
MCI/early-stage AD classifier	0.17 (0.13)	1	1.8	.178
Established AD classifier	0.11 (0.08)	1	2.2	.140
**EMM 3: Classifier type × Recovery type → Timepoint**	**Mean (SEM) difference: timepoint 2 vs. 1**			
**Schizophrenia classifier**				
Non-recovery	0.20 (0.09)	1	**4.6**	**.032**
Preserved recovery	0.02 (0.03)	1	0.4	.542
**bvFTD classifier**				
Non-recovery	0.23 (0.09)	1	**5.6**	**.018**
Preserved recovery	0.02 (0.02)	1	0.3	.396
**MCI/early-stage AD classifier**				
Non-recovery	0.15 (0.08)	1	2.6	.108
Preserved recovery	0.02 (0.02)	1	0.5	.493
**Established AD classifier**				
Non-recovery	0.11 (0.07)	1	1.2	.277
Preserved recovery	0.00 (0.10)	1	0.02	.889

Abbreviations: *SEM* Standard error of the mean

© 2022 Koutsouleris N et al. *JAMA Psychiatry*.

eTable 12. Mixed-Effects Linear Model Analysis of Clinical Dementia Score Trajectories in Patients With MCI/Early-Stage AD and Healthy Controls (HC) Covering a 9-Years Follow-up Period

A significant stratification effect of high. vs. low scores on the CDR trajectories of both MCI/early-stage AD patients and healthy controls was found which was independent of specific neuroanatomical classifiers. Furthermore, we found independent effects of BrainAGE on CDR trajectories and significant interaction effects involving follow-up, and study group factors. See **eFigure 28** for a visual representation of classifier-stratified CDR trajectories in patients and controls.

**Main and interaction effects**	**df_1**	**df_2**	**F**	***P***
Follow-up interval	4	943.4	**75.4**	**<.001**
Classifier type	4	872.6	0.0	1.0
High vs. low classifier expression	1	1532.0	**141.0**	**<.001**
Study group	1	1663.2	**2196.9**	**<.001**
BrainAGE	1	1583.9	**85.5**	**<.001**
High vs. low classifier expression × Follow-up interval	4	914.0	**24.1**	**<.001**
High vs. low classifier expression × Follow-up interval × Study group	9	1007.4	**12.2**	**<.001**
BrainAGE × Follow-up interval	4	1001.2	**5.7**	<**.001**
BrainAGE × Follow-up interval × Study group	5	627.4	**19.9**	<**.001**
**EMM 1: Study group × Classifier type → High vs. low expression score**	**df_1**	**df_2**	**F**	***P***
**MCI/early-stage AD**				
All classifiers	1	1220.8	**125.8**	**<.001**
**Healthy controls**				
All classifier	1	1890.6	**24.0**	**<.001**

© 2022 Koutsouleris N et al. *JAMA Psychiatry*.

eFigure 1. Schematic representation of the analysis flow implemented in the study.

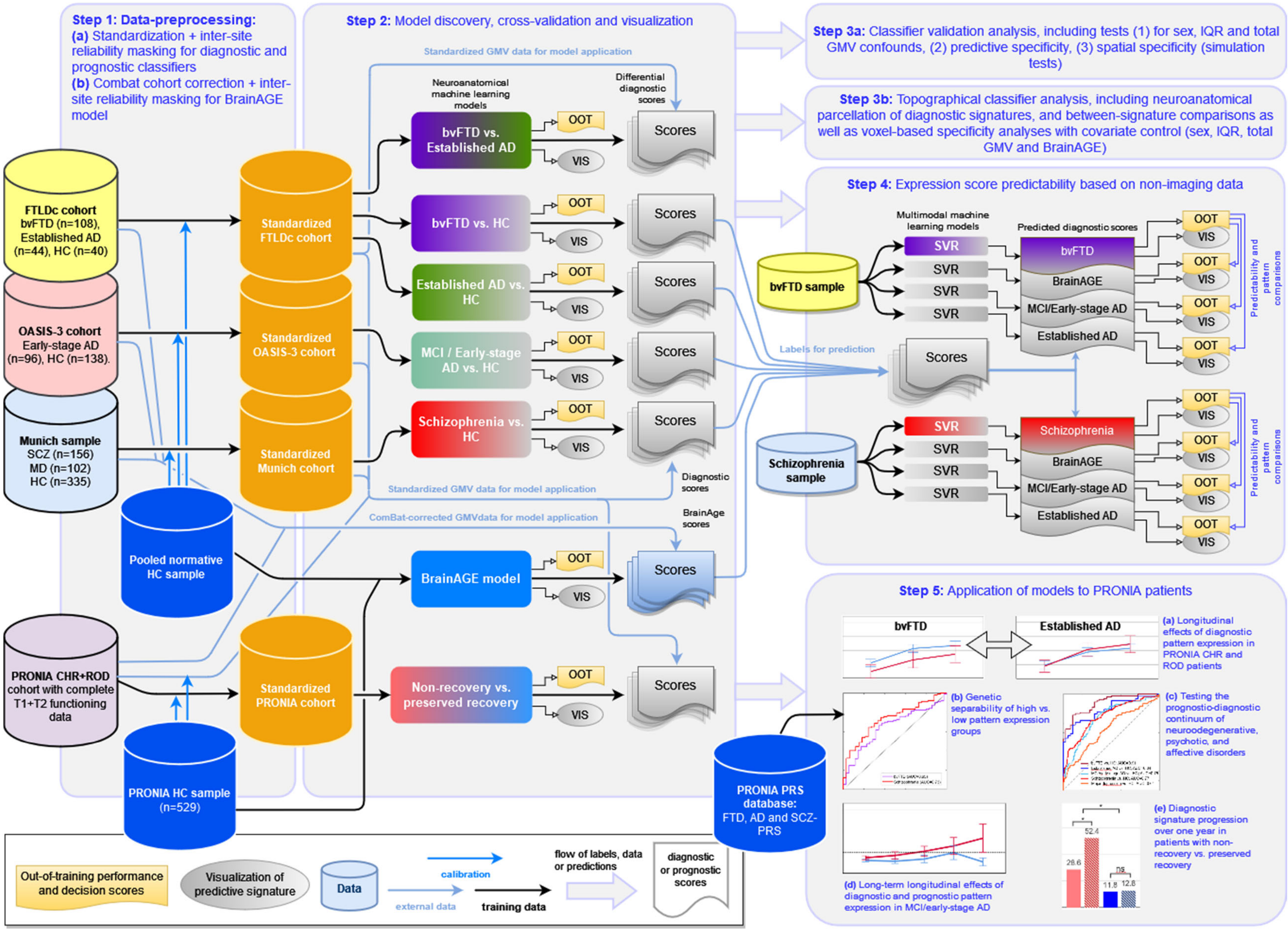

© 2022 Koutsouleris N et al. *JAMA Psychiatry*.

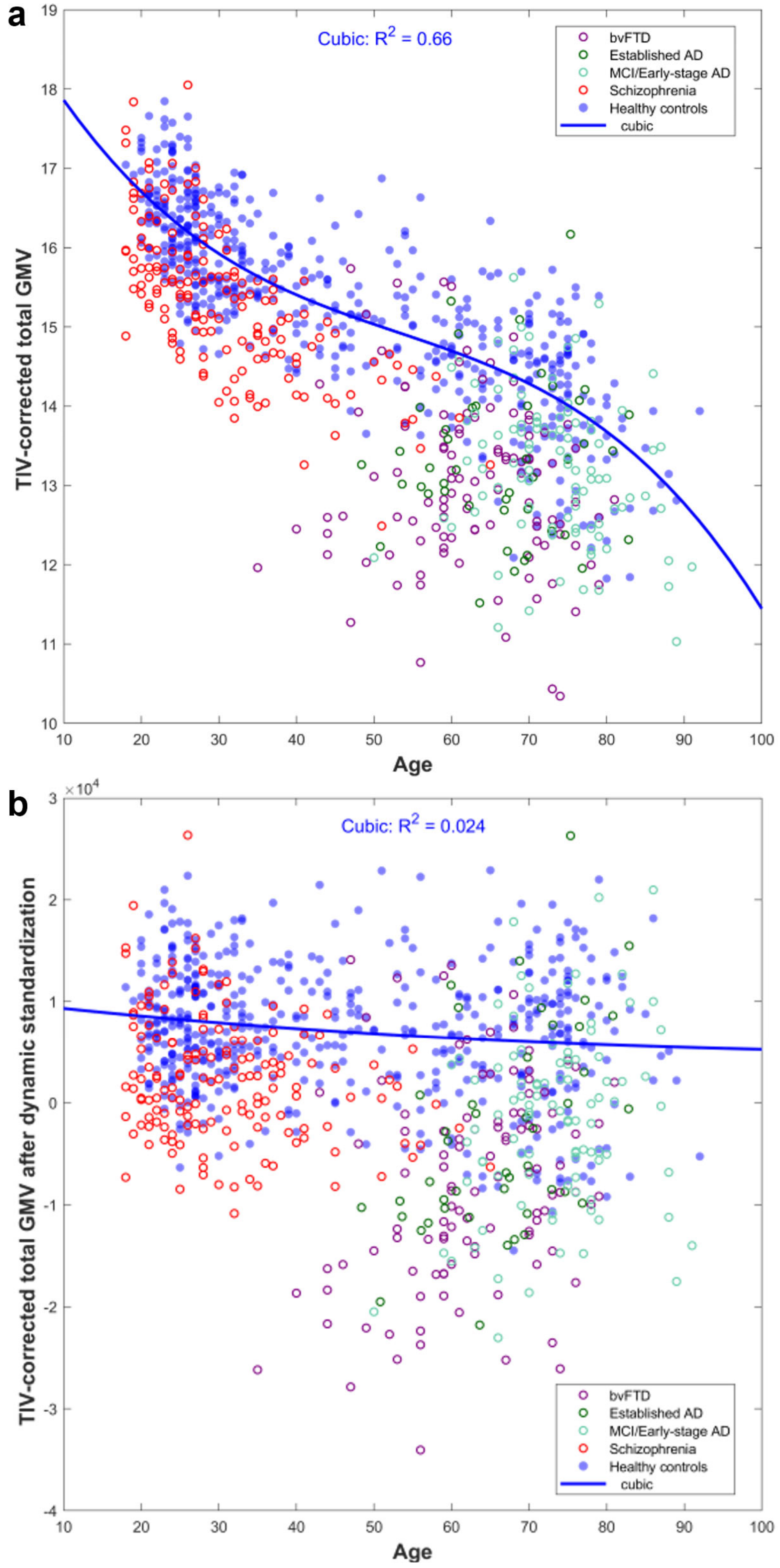

eFigure 2. Effects of the dynamic voxel standardization on the relationship between age and total gray matter volume estimates. (a) The study participants' total GMV values were computed by summing up the voxel values in the respective TIV-corrected GMV tissue maps. Healthy participants' (blue filled circles) and patients' total GMV values were plotted against their chronological age. The age-GMV relationship was visualized by fitting the healthy participants' data with a cubic function and measuring the coefficient of determination (R^2) between both variables. **(b)** This procedure was repeated after dynamically standardizing the TIV-adjusted GMV tissue maps as described in the Supplementary Methods.

© 2022 Koutsouleris N et al. *JAMA Psychiatry*.

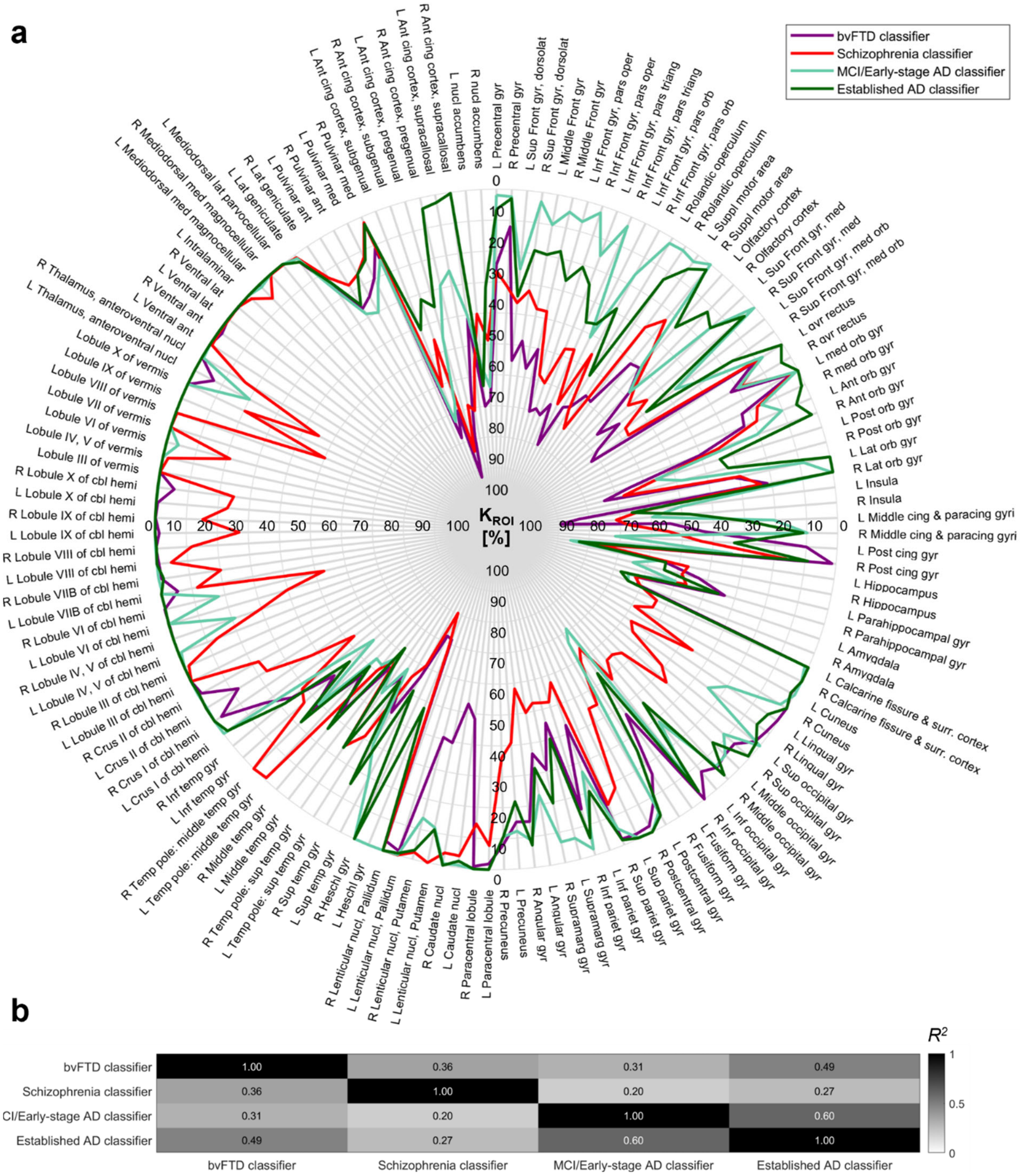

eFigure 3. Mapping of diagnostic signatures to the AAL3 atlas based on spatial extent. Each diagnostic classifier's cross-validation-ratio (CVR) signature was mapped to the 170 regions-of-interest (ROI) defined in the AAL3 atlas.[26] For each ROI, the percentage of voxels with an absolute CVR value ≥ 2 [K_{ROI}%] in the given intersecting signature volume was computed. If no voxels survived the CVR cutoff in any of the four signatures volumes, the given ROI was excluded from the mapping procedure. **(a)** The four K_{ROI}% vectors with 139 ROI entries are represented in a spider plot for the qualitative analysis of neuroanatomical overlaps and differences between the diagnostic signatures. **(b)** Pairwise coefficients of determination (R^2) between the K_{ROI}% mappings of the CVR signatures are displayed as an R^2 matrix. **Abbreviations:** *Ant* Anterior, *Cbl* Cerebellum, *Cing* Cingulate, *Dorsolat* Dorsolateral, *Front* Frontal, *Gyr* Gyrus, *Hemi* Hemisphere, *Inf* Inferior, *Lat* Lateral, *Med* Medial, *Nucl* Nucleus, *Orb* Orbital, *Post* Posterior, *Sup* Superior, *Supramarg* Supramarginal, *Suppl* Supplementary, *Surr* Surrounding, *Temp* Temporal.

© 2022 Koutsouleris N et al. *JAMA Psychiatry*.

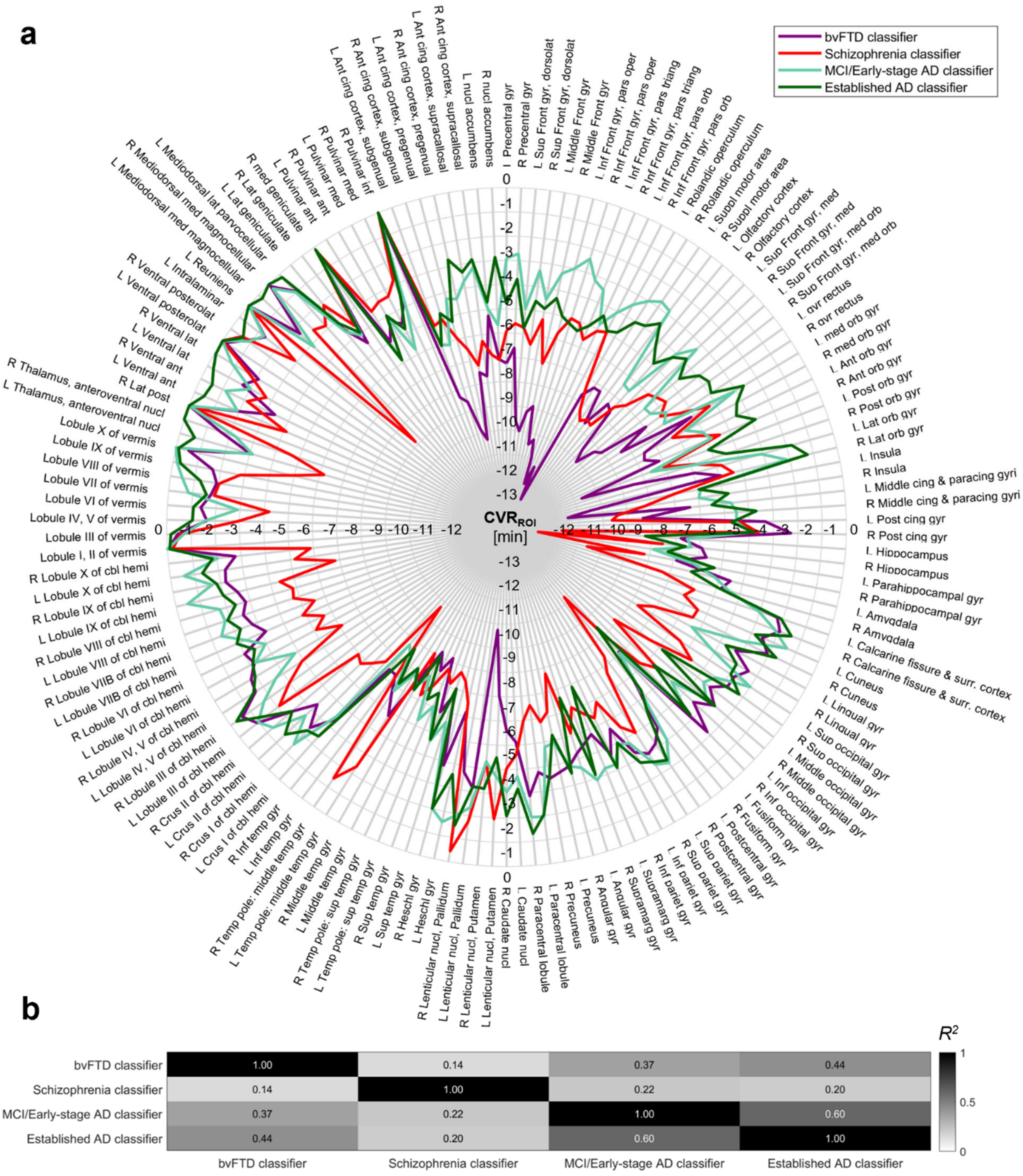

eFigure 4. Mapping of diagnostic signatures to the AAL3 atlas based on peak CVR values. In regions-of-interest (ROI) with at least one absolute CVR value ≥ 2 in any classifier signature (see **eFigure 3**), the peak CVR value (CVR_{ROI}[min]) of each classifier (bvFTD, schizophrenia, MCI/Early-stage AD, Established AD) was determined. The CVR_{ROI}[min] parcellations of all four classifiers are displayed in spider plots. **(b)** Pairwise associations between the four CVR_{ROI}[min] parcellations are expressed as coefficients of determination and displayed in an R^2 matrix. **Abbreviations:** *Ant* Anterior, *Cbl* Cerebellum, *Cing* Cingulate, *Dorsolat* Dorsolateral, *Front* Frontal, *Gyr* Gyrus, *Hemi* Hemisphere, *Inf* Inferior, *Lat* Lateral, *Med* Medial, *Nucl* Nucleus, *Orb* Orbital, *Post* Posterior, *Sup* Superior, *Supramarg* Supramarginal, *Suppl* Supplementary, *Surr* Surrounding, *Temp* Temporal.

© 2022 Koutsouleris N et al. *JAMA Psychiatry*.

a

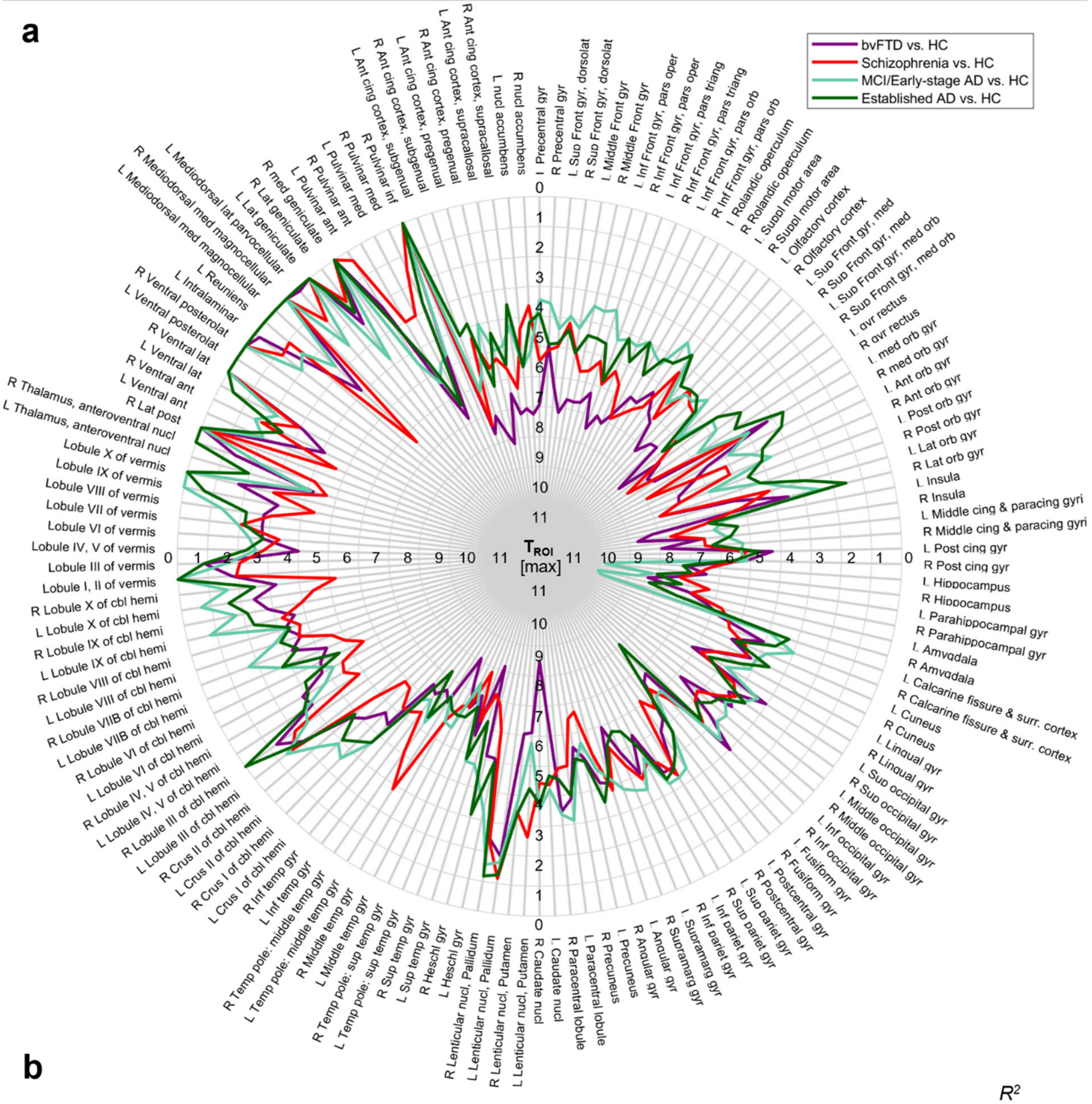

b

	bvFTD vs. HC	Schizophrenia vs. HC	MCI/Early-stage AD vs. HC	Established AD vs. HC
bvFTD vs. HC	1.00	0.44	0.52	0.58
Schizophrenia vs. HC	0.44	1.00	0.27	0.27
MCI/Early-stage AD vs. HC	0.52	0.27	1.00	0.65
Established AD vs. HC	0.58	0.27	0.65	1.00

R^2 (scale 0 – 0.5 – 1)

eFigure 5. Univariate group-level differences between patients and healthy controls in the AAL3 atlas. To compare the peak CVR measures of neuroanatomical deviation (**eFigure 4**) to univariate T values metrics, all voxels in the selected ROIs were assessed for reductions of standardized GMV in respective patients vs. healthy controls using independent two-sample T tests. **(a)** For each univariate group-level comparison (bvFTD vs. HC, schizophrenia vs. HC, MCI/Early-stage AD vs. HC, Established AD vs. HC), the peak T values in each ROI (T_{ROI}[max]) are displayed in a spider plot. **(b)** Pairwise associations of these four T_{ROI}[max] vectors are analyzed using the coefficient of determination and displayed as an R^2 matrix. **Abbreviations:** *Ant* Anterior, *Cbl* Cerebellum, *Cing* Cingulate, *Dorsolat* Dorsolateral, *Front* Frontal, *Gyr* Gyrus, *Hemi* Hemisphere, *Inf* Inferior, *Lat* Lateral, *Med* Medial, *Nucl* Nucleus, *Orb* Orbital, *Post* Posterior, *Sup* Superior, *Supramarg* Supramarginal, *Suppl* Supplementary, *Surr* Surrounding, *Temp* Temporal.

© 2022 Koutsouleris N et al. *JAMA Psychiatry*.

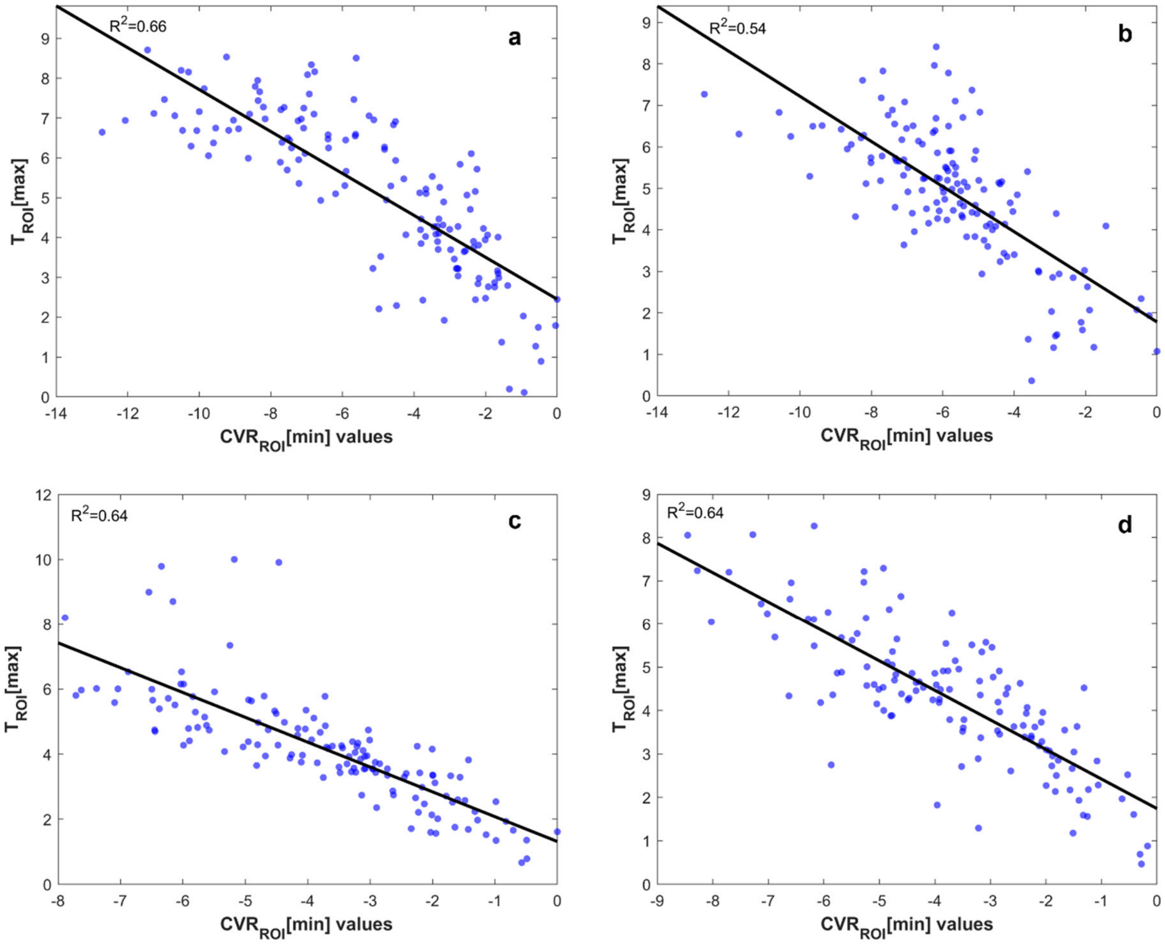

eFigure 6. Associations between multivariate and univariate measures of neuroanatomical differences between patients and healthy controls. Scatter plots with fitted slope lines and coefficients of determination (R^2) depict the correlations between ROI-based CVR_{ROI}[min] and T_{ROI}[max] vectors in each of the four diagnostic comparisons (**a:** bvFTD vs HC, **b:** Schizophrenia vs. HC, **c:** MCI/Early-stage AD vs. HC, **d:** Established AD vs. HC).

© 2022 Koutsouleris N et al. *JAMA Psychiatry*.

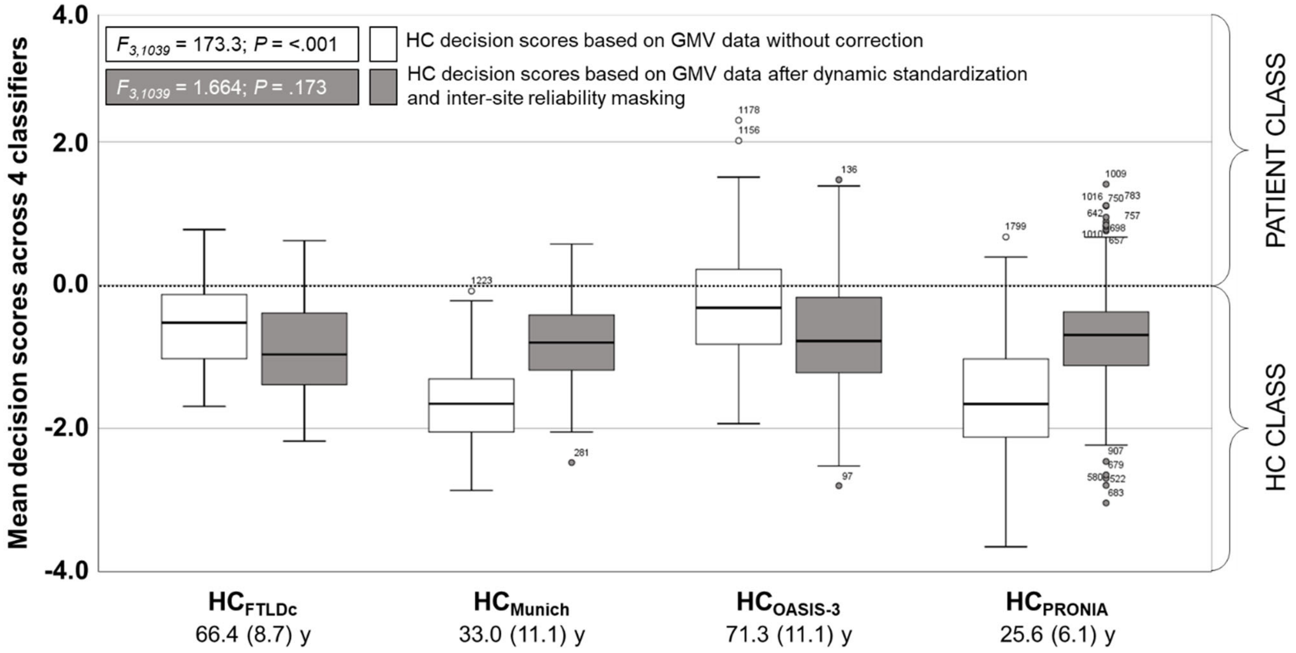

eFigure 7. Validation of the cohort adjustment strategy. A box plot analysis was conducted to compare of training classifiers with vs. without our cohort adjustment procedure. White box plots quantified the distributions of HCs' mean decision scores that were calculated by averaging the output of the four classifiers trained on unadjusted GMV data. In contrast, grey box plots show HC individuals' mean decision score distributions resulting from training classifiers on adjusted GMV, followed by inter-site reliability masking of adjusted GMV maps. Two ANOVAs were performed to assess cohort differences in unadjusted and adjusted mean decision scores.

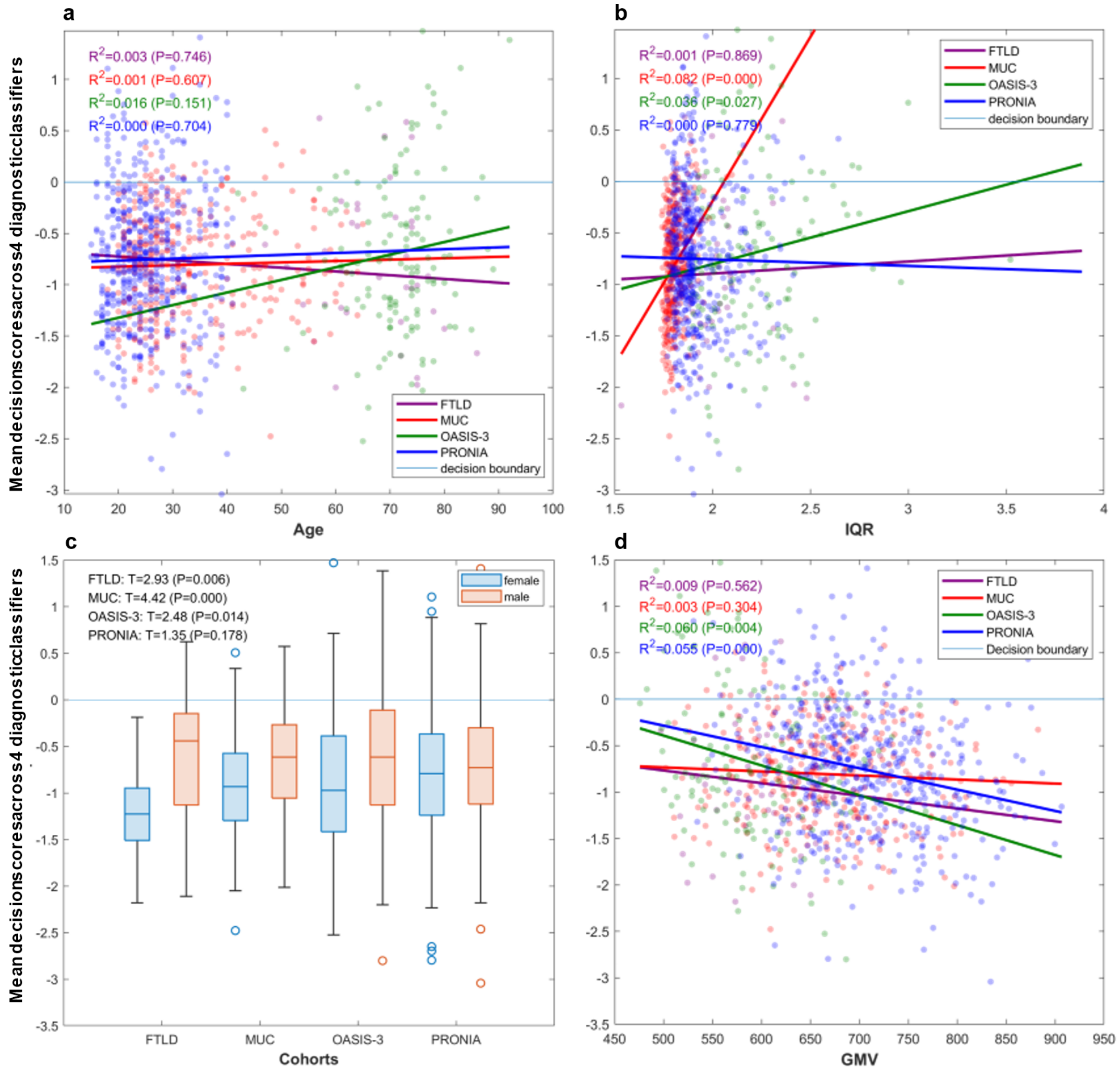

eFigure 8. Covariate effects on classifiers' decision scores in the different healthy control samples of the study. Scatter plots (**a**, **b**, **d**) were used to investigate associations between potential confounding effects of age (**a**), image quality ratings (**b**; IQR, higher = lower quality), and total GMV (**d**) on the HC participants mean decision scores, computed across the four diagnostic classifiers. Effects of sex were analyzed by means of box plots indicating 95% confidence intervals, upper and lower quartiles, and medians of respective mean decision score distributions. To identify significant associations between potential confounders and mean decision scores at the cohort-level, R^2 values were computed for continuous measures, and student t tests were conducted for sex-related differences. Potentially relevant effects were detected for sex, IQR and total GMV and further analyzed in **eTable 5**.

© 2022 Koutsouleris N et al. *JAMA Psychiatry*.

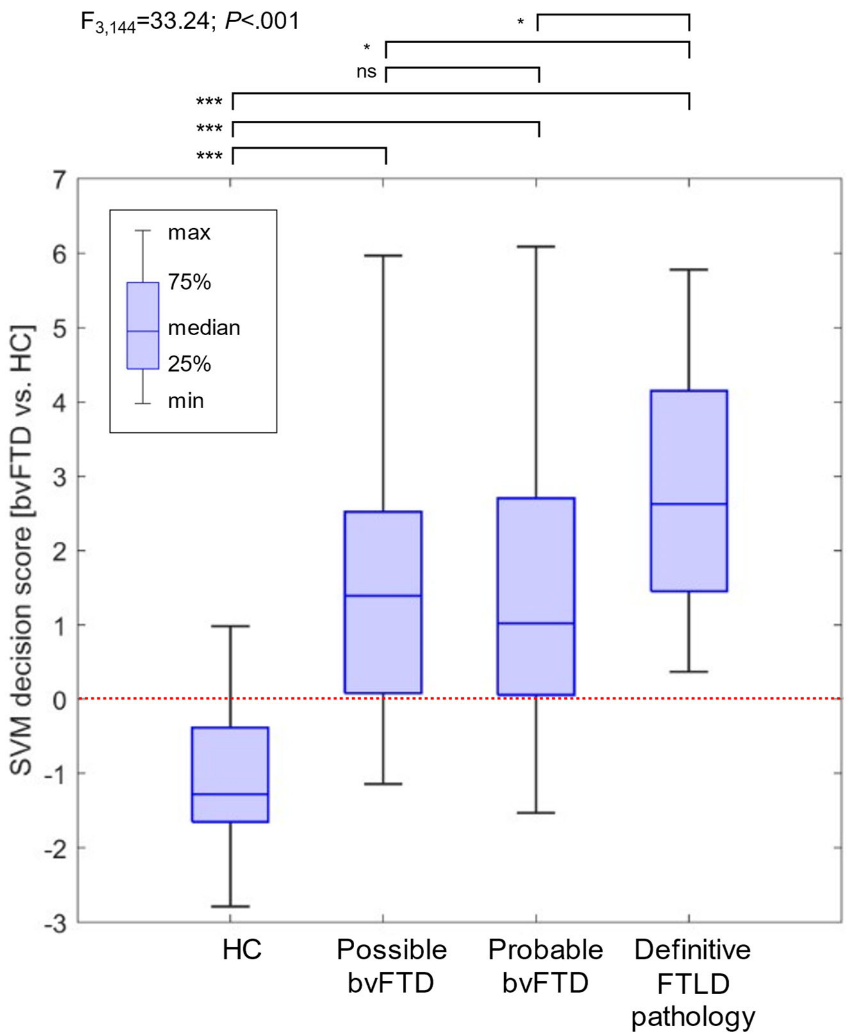

eFigure 9. Post-hoc evaluation of interactions between bvFTD decision scores and diagnostic criteria of bvFTD subgrouping patients into possible, probable, and definitive frontotemporal lobar degeneration (FTLD). **(a)** Box plot analysis of decision score distribution according to diagnostic subgroups. The omnibus ANOVA analysis was significant (F=33.2; P<.001). Pairwise post-hoc comparisons revealed significant differences between the decision scores of HC and the two bvFTD subgroups, respectively, as well as between patients with definitive FTLD pathology and all other diagnostic groups. No differences were found between patients with probable vs. possible FTD.

© 2022 Koutsouleris N et al. *JAMA Psychiatry*.

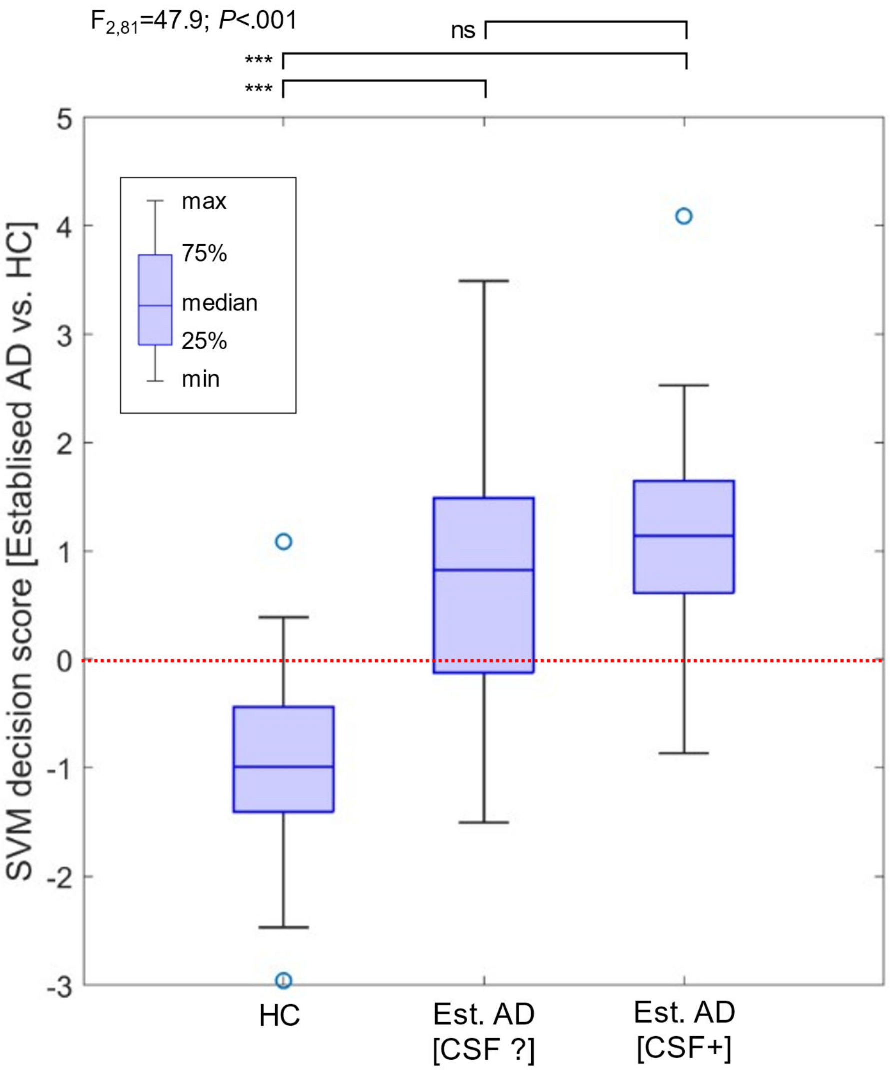

eFigure 10. Post-hoc evaluation of established AD decision scores in relation to the known or unknown CSF biomarker status in patients with established AD. CSF positive findings were defined by Aβ1-42 <550 pg/ml or Tau protein >300 pg/ml. **(a)** Box plot analysis of decision score distributions according to study group (HC, n=40; positive CSF biomarker findings [CSF+], n=31, or unknown CSF biomarker status [CSF?], n=13). The omnibus ANOVA analysis was significant (F=45.7; P<.001). Pairwise comparisons revealed significant decision score difference between HC and both AD subgroups, but no differences between the CSF+ and CSF? AD patients.

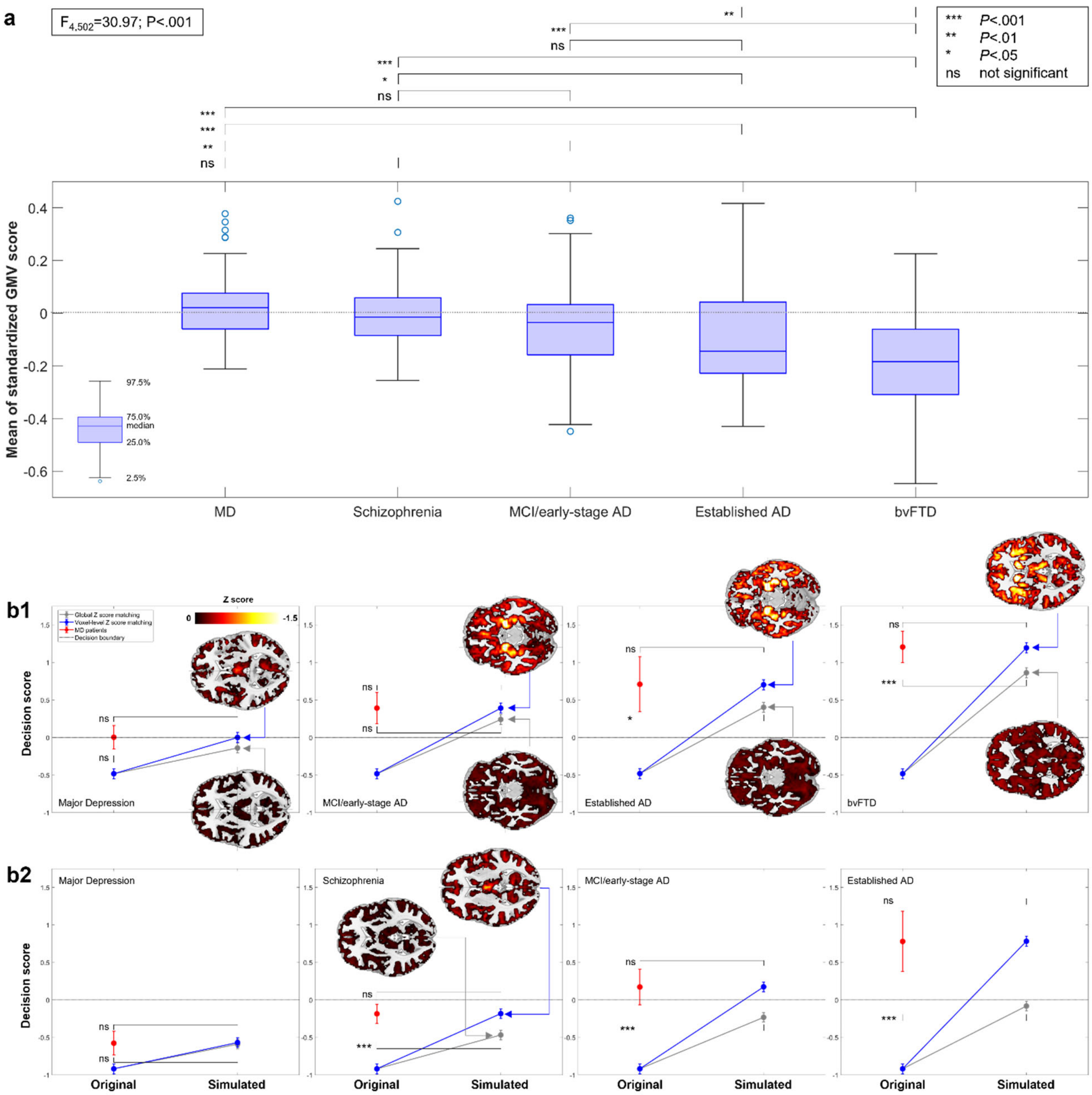

eFigure 11. Probing the bvFTD and schizophrenia classifiers' spatial specificity via atrophy simulation. In **(a)** global levels of brain atrophy were compared patients with bvFTD, established AD, MCI/early-stage AD, schizophrenia, and major depression samples by computing the mean across standardized voxels in study participants' GVM maps and conducting an ANOVA on this measure. To test the schizophrenia **(b1)** and bvFTD (**b2**) classifiers for spatial specificity against these global GMV differences, they were applied to the HC individuals, who were pooled across the FTLDc, Munich and OASIS-3 cohorts, and whose GMV maps were systematically manipulated to make them patient-like. The null hypothesis of spatial non-specificity (grey lines) was created by calculating the mean difference between the HC group and the respective target patient group across all voxels in the inter-site reliability mask and subtracting this value from all voxels. For the alternative hypothesis (blue lines), the voxel-wise Z score difference image was computed between HC and target patient groups and subtracted from the HC sample. The HC sample's mean Z map for both simulation scenarios is shown for each targeted patient group. All maps were scaled in the Z score range from -1.5 to +1.5. Two-sample *t* tests were conducted to compare simulated with observed group-level decision scores of the respective target group and corrected classifier-wise for multiple comparisons using FDR (q=0.05).

© 2022 Koutsouleris N et al. *JAMA Psychiatry*.

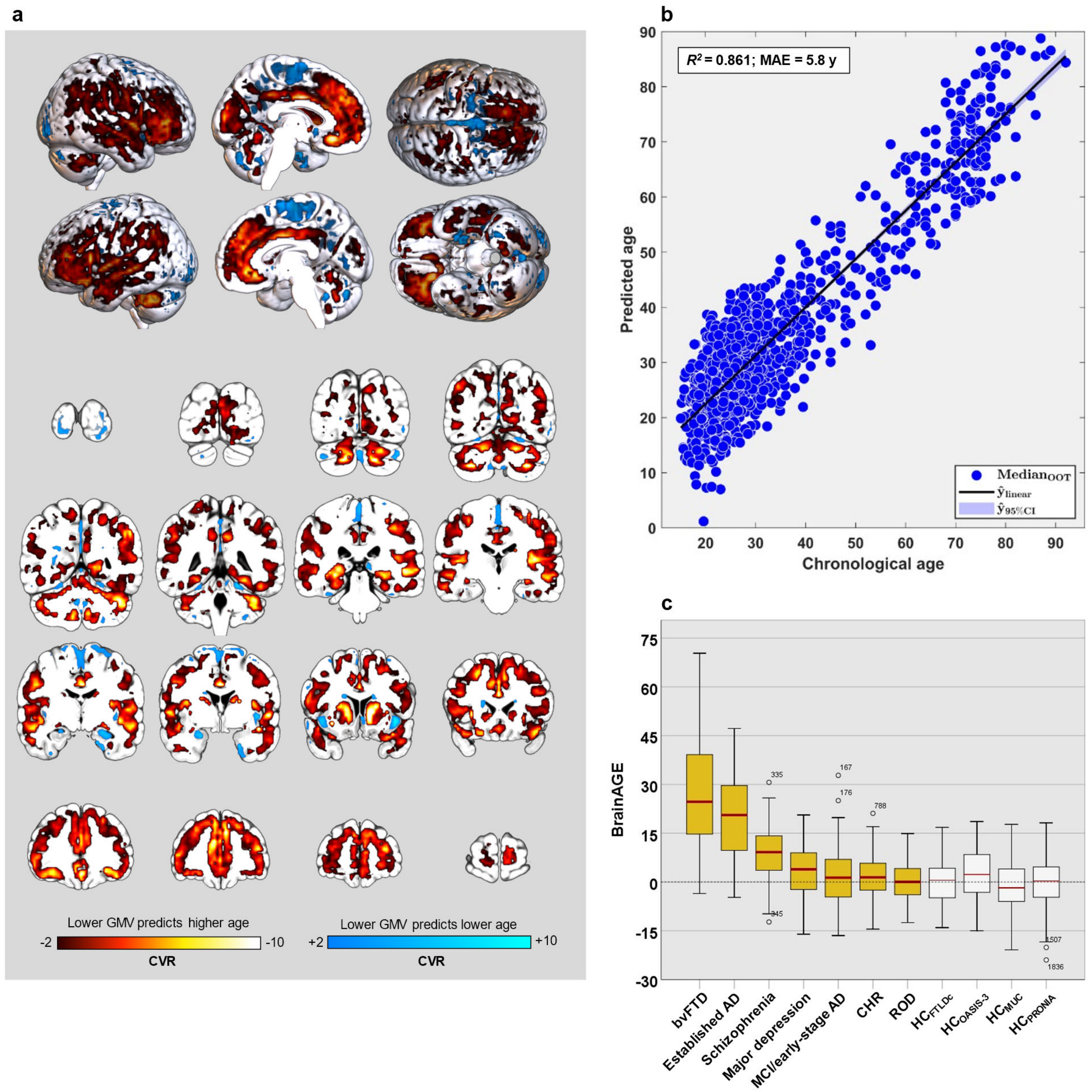

eFigure 12. Visualization and quantitative analysis of the BrainAGE model developed in the pooled HC cohort and applied to the clinical samples of the study. (**a**) Reliability of neuroanatomical pattern elements as measured by cross-validation ratio mapping. (**b**) Prediction performance of the model in unseen HC data described visually by plotting predicted vs. chronological age as well as numerically in terms of the model's mean average error (MAE) and coefficient of determination (R^2). (**c**) Box plot analysis comparing the distributions of the patients' (ochre boxes) and HC individuals' (white boxes) BrainAGE scores.

© 2022 Koutsouleris N et al. *JAMA Psychiatry*.

a

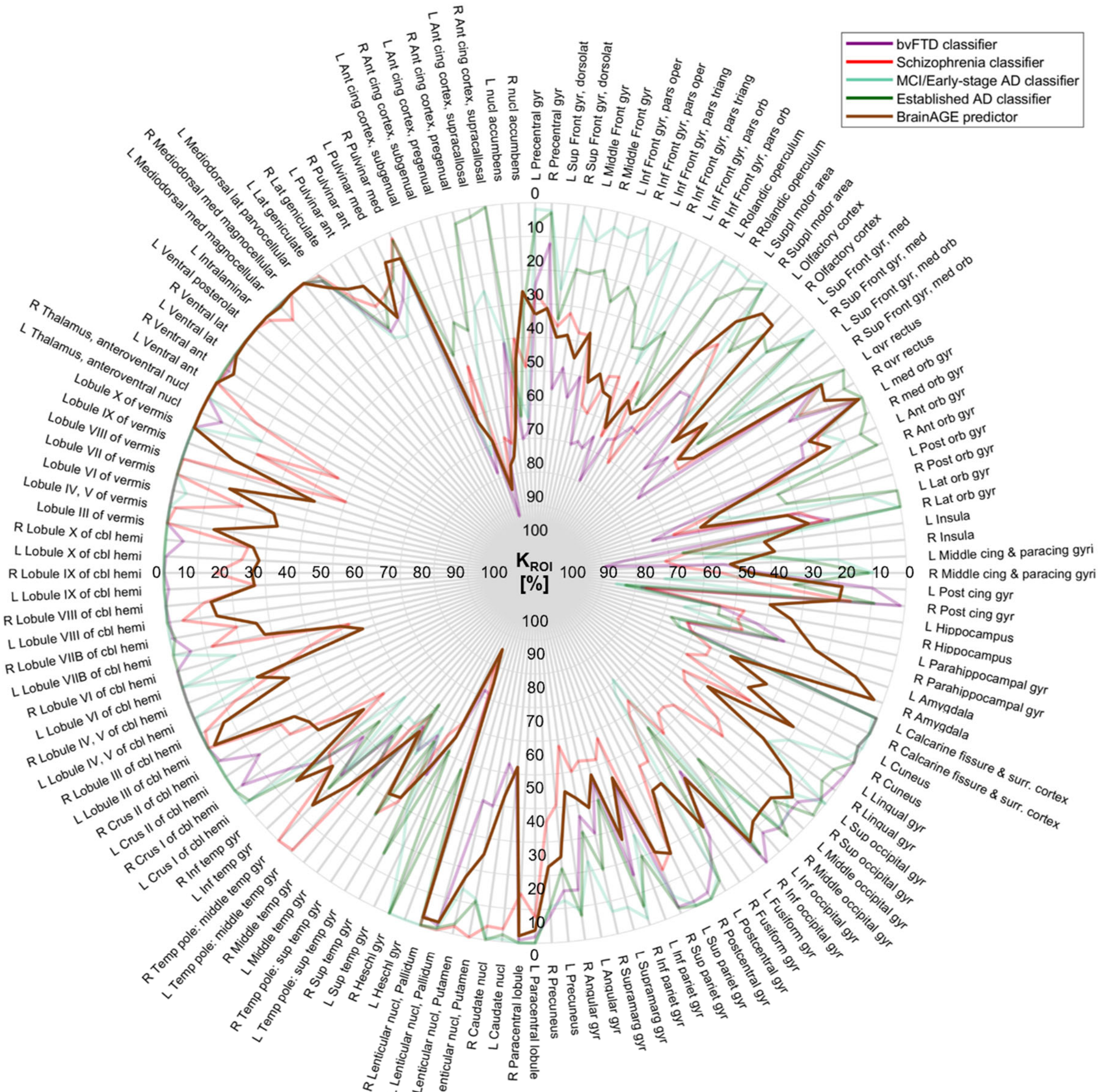

b

	bvFTD classifier	Schizophrenia classifier	MCI/Early-stage AD classifier	Established AD classifier	BrainAGE predictor
bvFTD classifier	1.00	0.37	0.31	0.49	0.46
Schizophrenia classifier	0.37	1.00	0.20	0.27	0.47
MCI/Early-stage AD classifier	0.31	0.20	1.00	0.60	0.05
Established AD classifier	0.49	0.27	0.60	1.00	0.12
BrainAGE predictor	0.46	0.47	0.05	0.12	1.00

R^2 (scale 0 – 0.5 – 1)

eFigure 13. Mapping of the BrainAGE signature to the AAL3 atlas based on spatial extent. See legend of **eFigure 3** and Supplementary Methods for a description of the mapping procedure. The K_{ROI}% parcellations of the four diagnostic classifiers were added to the spider plot in transparent colors for comparison with the non-recovery predictor. **Abbreviations:** *Ant* Anterior, *Cbl* Cerebellum, *Cing* Cingulate, *Dorsolat* Dorsolateral, *Front* Frontal, *Gyr* Gyrus, *Hemi* Hemisphere, *Inf* Inferior, *Lat* Lateral, *Med* Medial, *Nucl* Nucleus, *Orb* Orbital, *Post* Posterior, *Sup* Superior, *Supramarg* Supramarginal, *Suppl* Supplementary, *Surr* Surrounding, *Temp* Temporal.

© 2022 Koutsouleris N et al. *JAMA Psychiatry*.

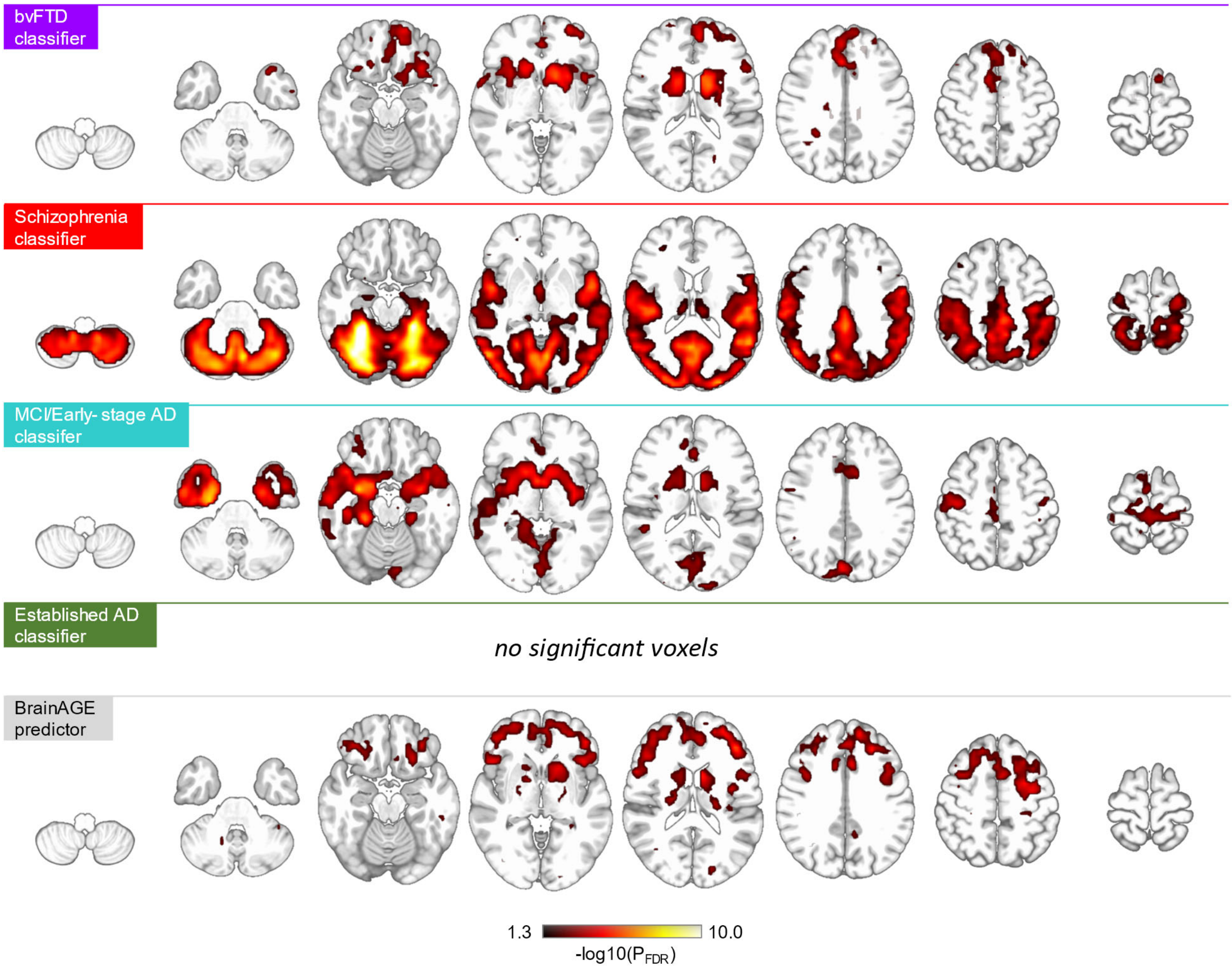

eFigure 14. Univariate voxel-based spatial specificity results showing covariate-corrected negative associations between diagnostic expression scores and dynamically standardized GMV maps of patients with bvFTD (n=108) and healthy controls (n=40). The analyses were conducted using statistical non-parametric mapping (5000 permutations) as implemented in the Threshold-Free Cluster Enhancement (TFCE) toolbox for SPM12 (http://www.neuro.uni-jena.de/tfce/). All *P* value maps were corrected for multiple comparisons using FDR (significance threshold: q=0.05).

© 2022 Koutsouleris N et al. *JAMA Psychiatry*.

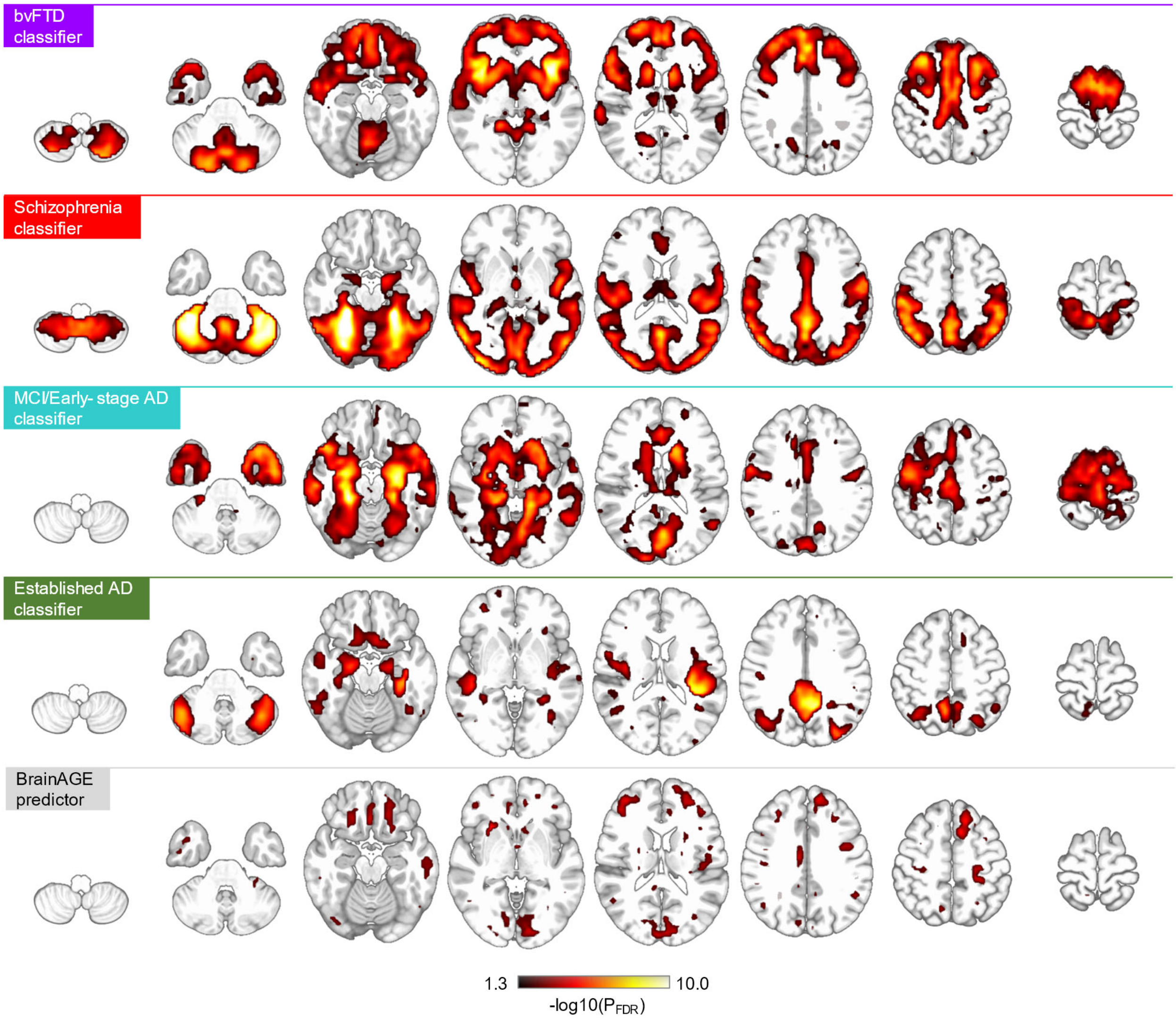

eFigure 15. Univariate voxel-based spatial specificity results showing covariate-corrected negative associations between diagnostic expression scores and dynamically standardized GMV maps of a pooled sample of patients with schizophrenia (n=157), major depression (n=102) and healthy controls (n=335). The analyses were conducted using statistical non-parametric mapping (5000 permutations) as implemented in the Threshold-Free Cluster Enhancement (TFCE) toolbox for SPM12 (http://www.neuro.uni-jena.de/tfce/). All *P* value maps were corrected for multiple comparisons using FDR (significance threshold: q=0.05).

© 2022 Koutsouleris N et al. *JAMA Psychiatry*.

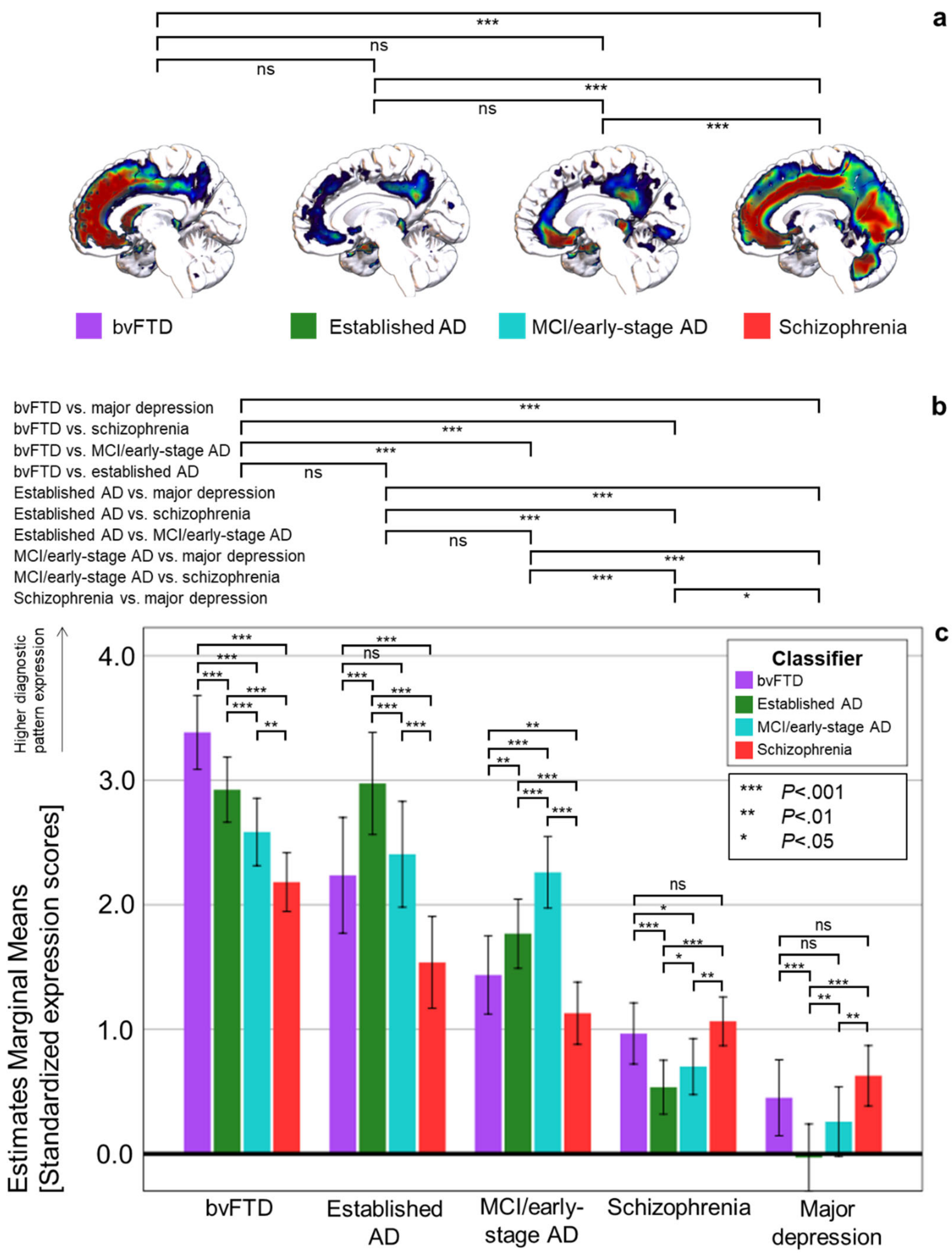

eFigure 16. Results of the repeated-measures analysis of variance comparing diagnostic expression Scores between patient groups. The ANOVA compared main effects of (**a**) standardized neuroanatomical expression scores for bvFTD, established AD, MCI/early-stage AD, schizophrenia), (**b**) diagnosis (bvFTD, established AD, MCI/early-stage AD, schizophrenia, major depression), and (**c**) diagnosis-by-classifier interaction effects. Bar plots show estimated marginal means and 95% confidence intervals of standardized neuroanatomical expression scores (see Methods) with higher z scores indicating more pronounced group-level expression of given diagnostic pattern by the respective patient group. See also **eFigure 17** for a BrainAGE-adjusted version of the analysis.

© 2022 Koutsouleris N et al. *JAMA Psychiatry*.

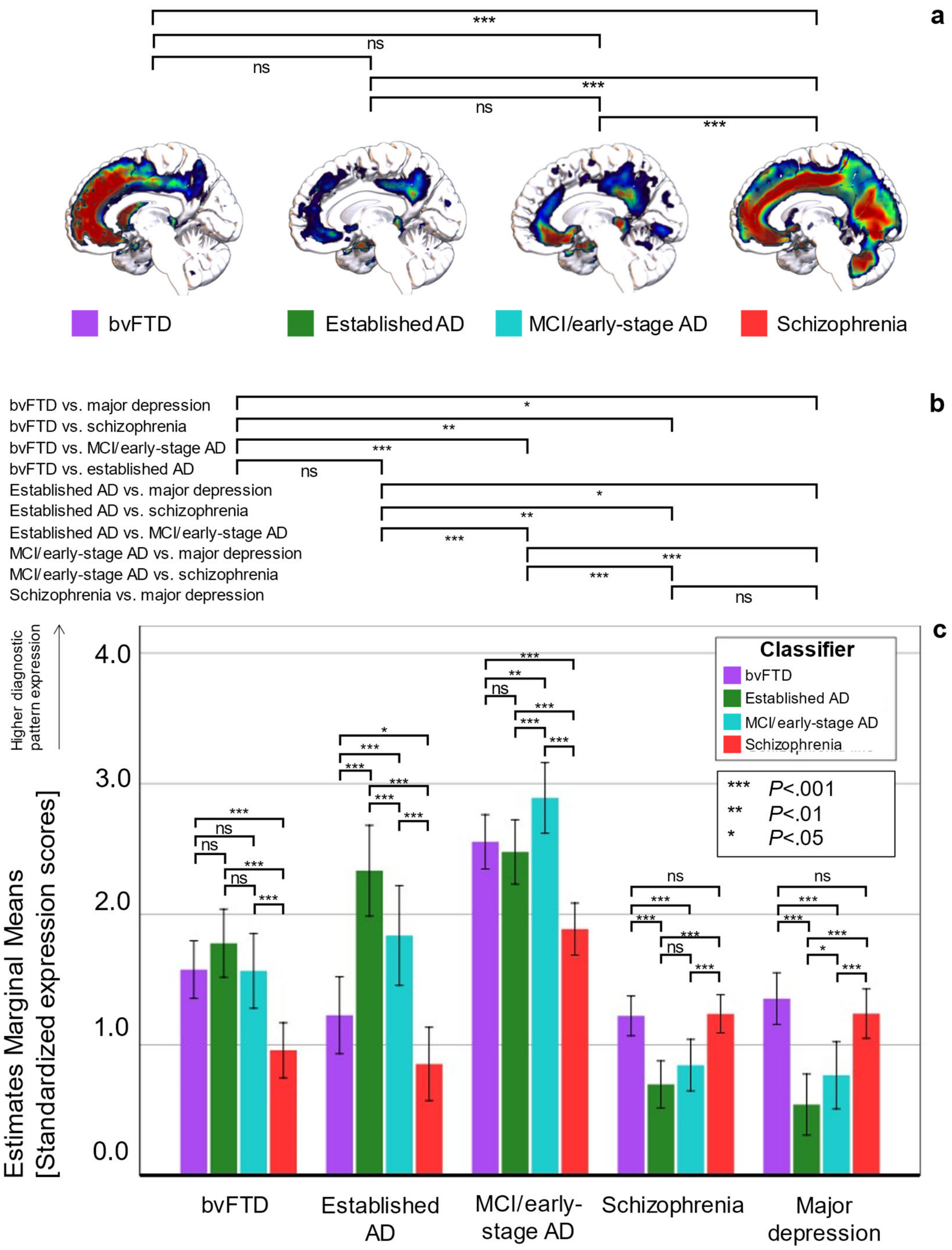

eFigure 17. Repeated-measures analysis of variance comparing patient groups' diagnostic expression scores with BrainAGE included as a covariate Estimated marginal means comparisons comprised classifier effects (**a**), diagnostic effects (**b**), and classifier-by-diagnosis interactions (**c**). *P* values were corrected for multiple comparisons using Sidak's method.[29] Estimated marginal means of standardized diagnostic scores were reduced in the bvFTD and established AD group, while increased scores were found in the MCI/early-stage AD, schizophrenia and MD samples compared with the uncorrected analysis. BrainAGE residualization reduced between-group differences, particularly in bvFTD and established AD compared with the other samples, and in schizophrenia with respect to MD, suggesting a significant contribution of BrainAGE to the diagnostic separability of these study groups. However, the difference between bvFTD and established AD expression scores in patients with schizophrenia or MD remained significant, indicating a specific loading of these patients on the bvFTD signature beyond the variance explained by BrainAGE.

© 2022 Koutsouleris N et al. *JAMA Psychiatry*.

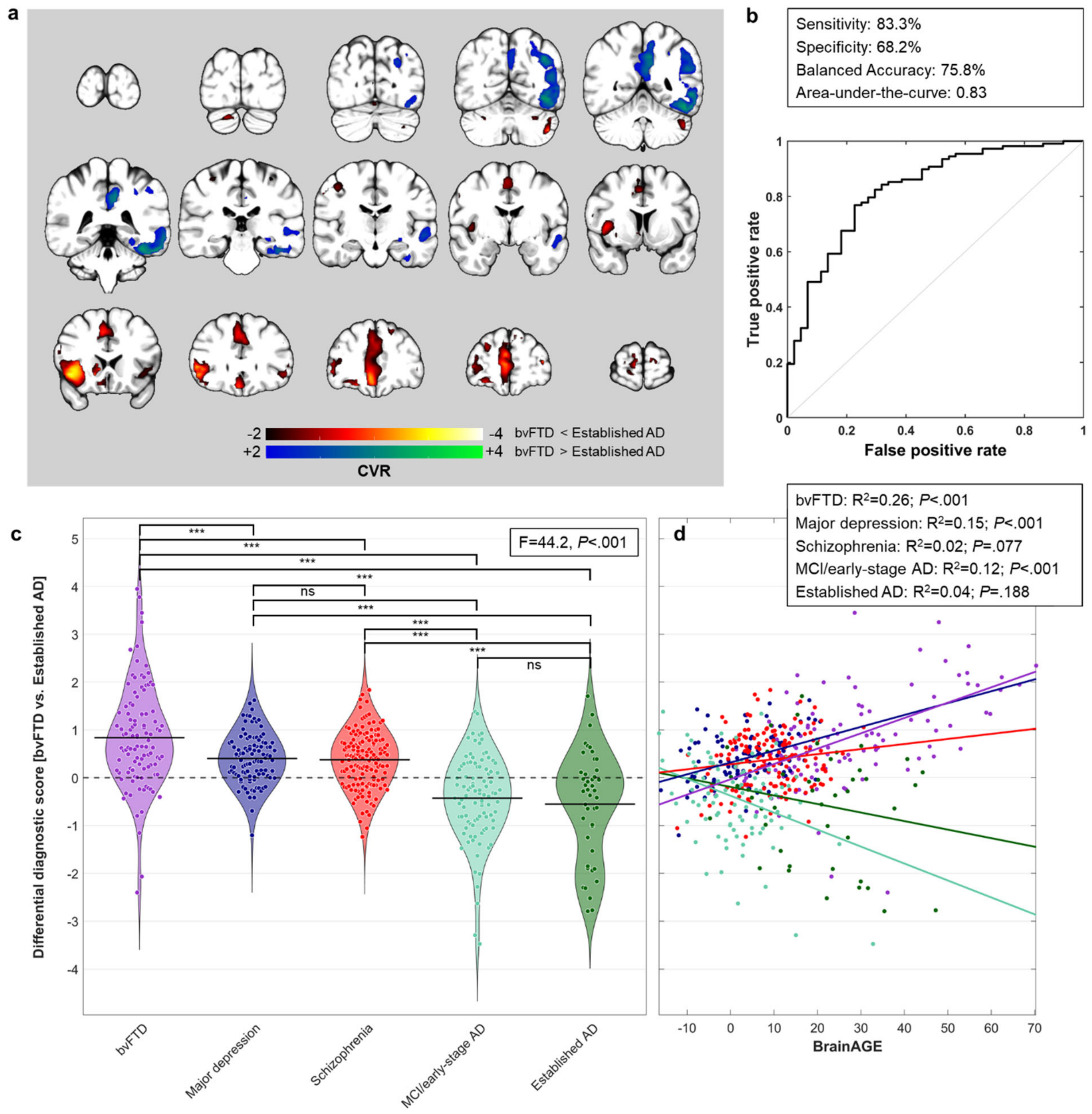

eFigure 18. Differential diagnostic classifier trained to separate between bvFTD and established AD and applied to patients with schizophrenia, major depression and MCI/early-stage AD. (**a**) The neuroanatomical pattern of the classifier involved relative, left-lateralized insular, medial prefrontal and cingulate cortex volume reductions in bvFTD compared to established AD, as well as relative, right-lateralized volume reductions in the temporal and medial as well as lateral areas of the parieto-occipital cortex in patients with established AD vs. bvFTD. (**b**) The cross-validated performance of the classifier was higher in bvFTD (83.3% correctly classified) compared to AD (68.2%) patients. (**c**) Violin plots show the decision score distributions of the derivation cohorts (bvFTD, left; established AD, right) and of the application samples (major depression, schizophrenia, MCI/early-stage AD). ANOVA results and post-hoc pairwise tests (Sidak correction for multiple comparisons) confirmed that psychiatric patient cohorts align neuroanatomically with bvFTD, while patients with MCI/early-stage AD are similar to established AD. (**d**) Interaction analysis between BrainAGE, and differential diagnostic scores demonstrated that higher BrainAGE was associated with widening neuroanatomical differences between the classifier's derivation samples. This effect was also present between the two psychiatric samples on the one hand, and MCI/early-stage AD patients on the other hand.

© 2022 Koutsouleris N et al. *JAMA Psychiatry*.

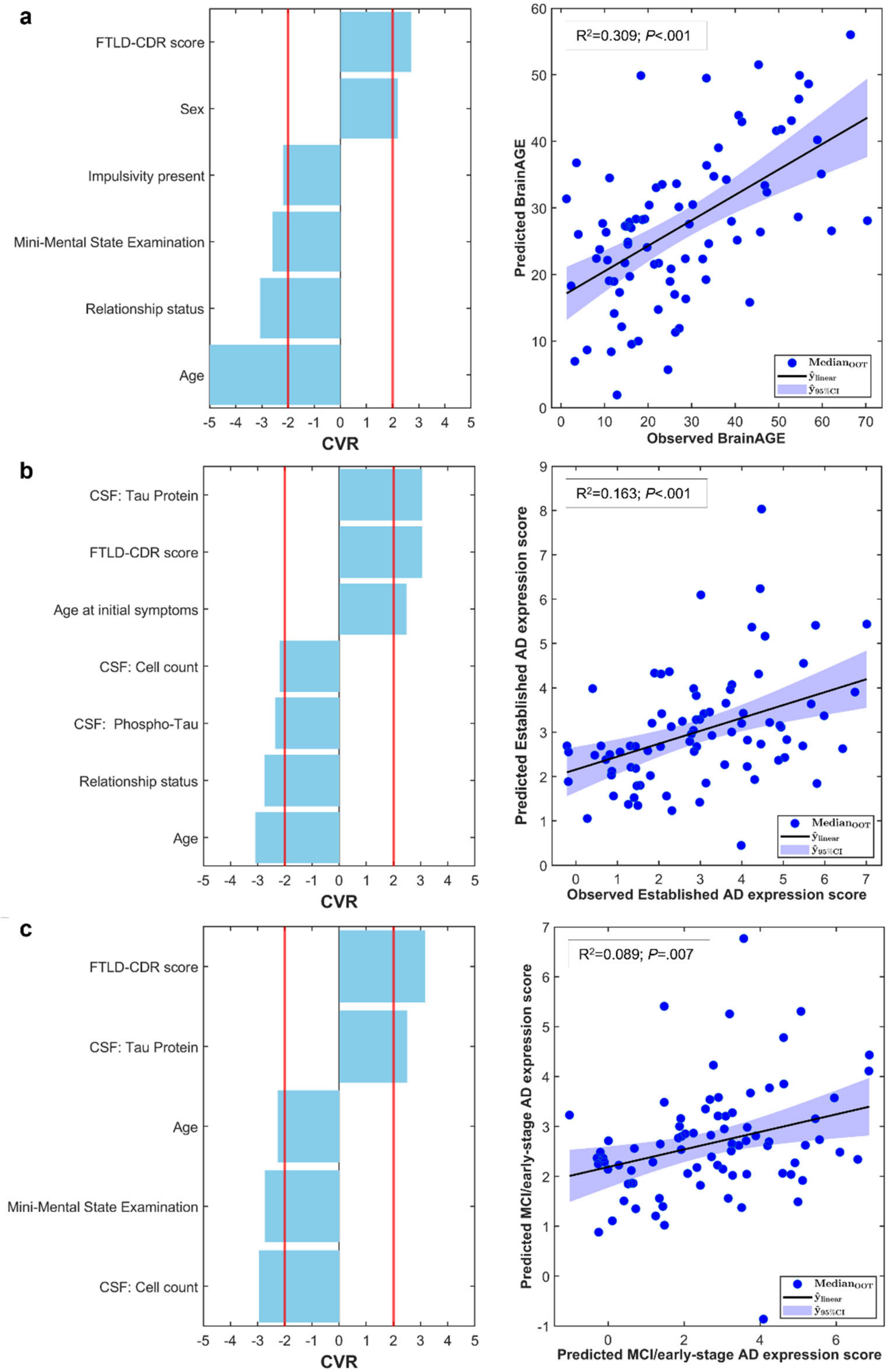

eFigure 19. Analysis of SVR models predicting bvFTD patients' BrainAGE and diagnostic expression scores. Panels show predictive features and scatter plots of BrainAGE **(a)**, Established AD **(b)**, and MCI/early-stage AD **(c)** score predictors. This eFigure complements **Figure 2** in the main manuscript. Bar plots show the ranked reliability (cross-validation ratio, CVR) of features informing the SVR models' predictions. Positive/negative CVR values indicate positive/negative predictive associations between features and observed scores. Scatter plots with linear fits, 95% confidence intervals and explained variances (R^2) describe the accuracy of the predictive model.

© 2022 Koutsouleris N et al. *JAMA Psychiatry*.

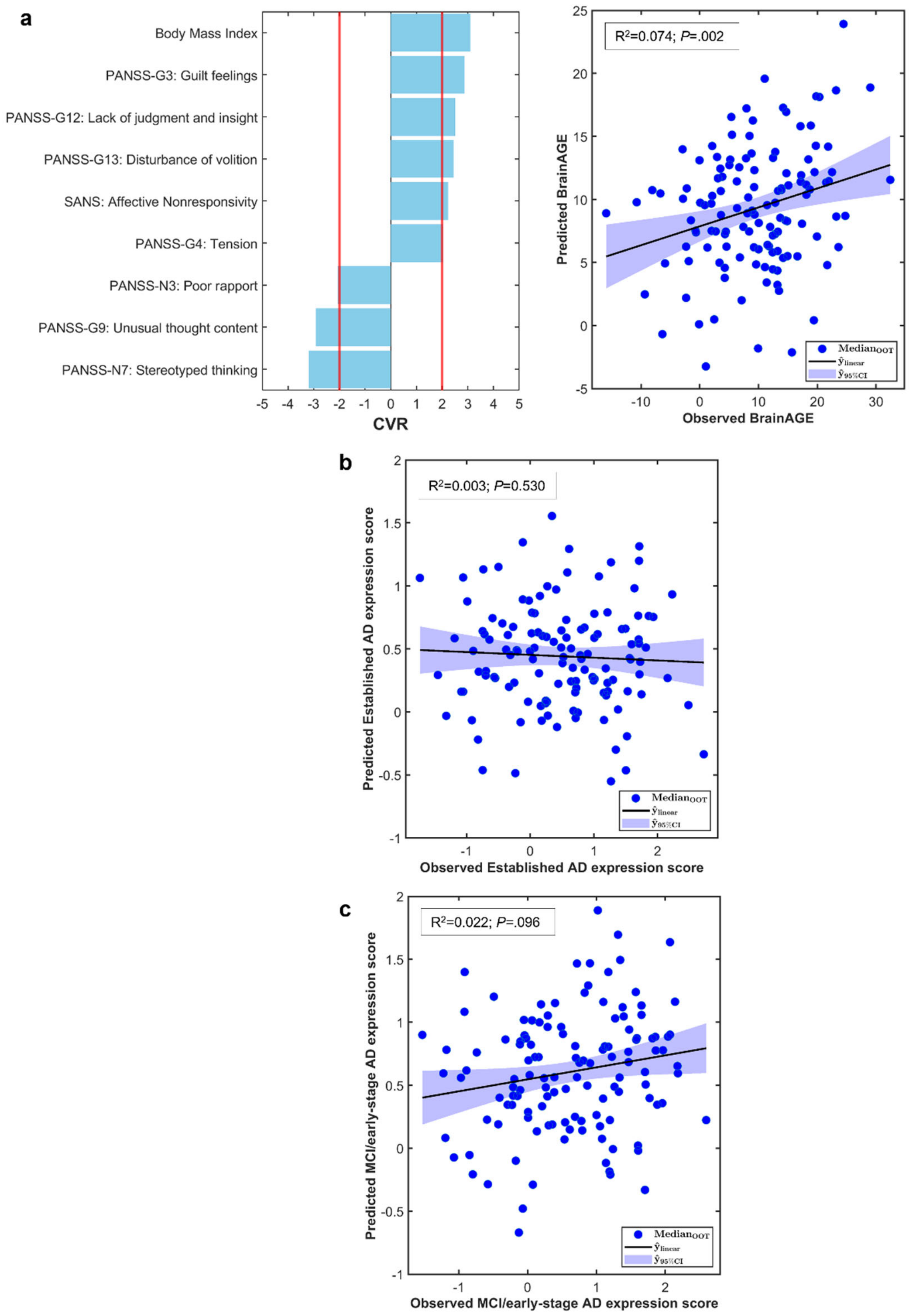

eFigure 20. Analysis of SVR models predicting schizophrenia patients' BrainAGE and diagnostic expression scores. Panels show predictive features and scatter plots of BrainAGE **(a)**, Established AD **(b)**, and MCI/early-stage AD **(c)** score predictors. Bar plots show the ranked reliability (cross-validation ratio, CVR) of features informing the SVR models' predictions. Positive/negative CVR values indicate positive/negative predictive associations between features and observed scores. Scatter plots with linear fits, 95% confidence intervals and explained variances (R^2) describe the accuracy of the predictive model. Features of non-significant models were omitted.

© 2022 Koutsouleris N et al. *JAMA Psychiatry*.

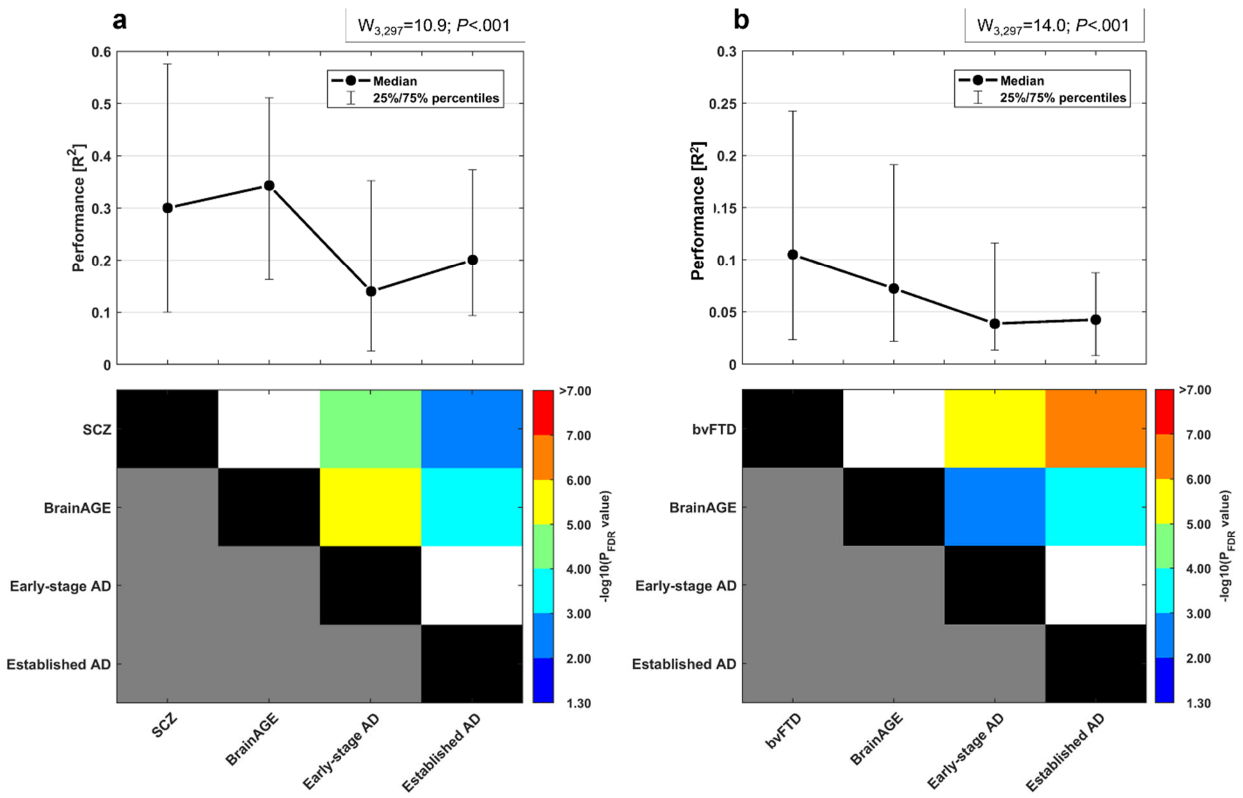

eFigure 21. Quade tests comparing the performance of the ν-SVR models in predicting diagnostic expression scores and BrainAGE in bvFTD patients (**a**) or schizophrenia patients (**b**). No significant differences were found in the schizophrenia (SCZ) expression scores and BrainAGE of bvFTD patients **(a)**, as well as in the bvFTD expression scores and BrainAGE of schizophrenia patients **(b)**. AD-related expression scores did not differ between each other in either group.

© 2022 Koutsouleris N et al. *JAMA Psychiatry*.

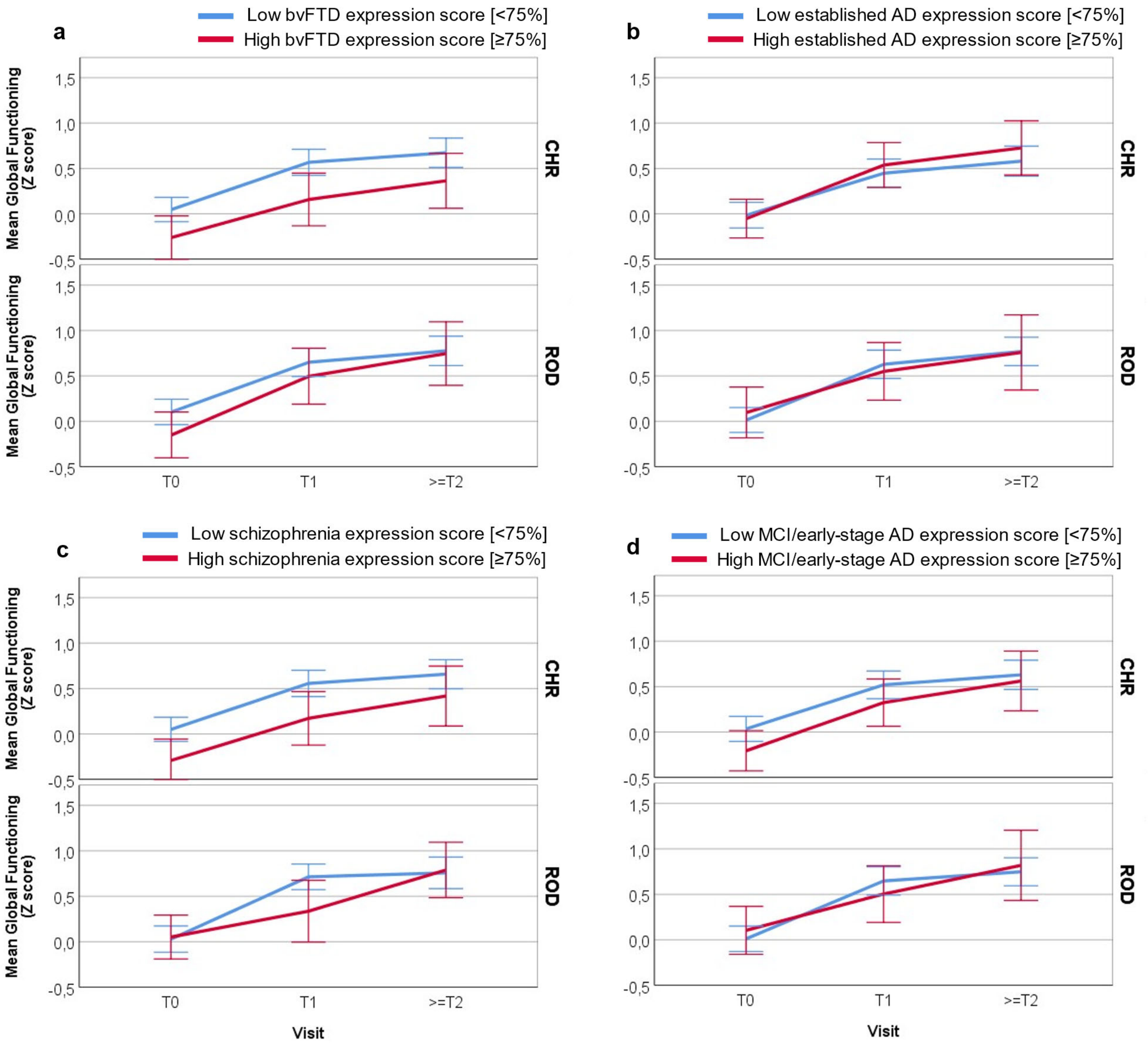

eFigure 22. Global functioning trajectories of the PRONIA cohort demonstrating interaction effects between bvFTD and schizophrenia pattern expression. Panels a-d depict global functioning trajectories of PRONIA study groups (CHR vs. ROD patients) stratified into high vs. low pattern expression groups by the bvFTD (**a**), Established AD (**b**), schizophrenia (**c**), and MCI/early-stage AD (**d**) classifiers . Red vs. blue line plots show estimated marginal means and 95% confidence intervals of standardized global functioning trajectories computed for high vs. low pattern expression groups. Groups were defined by a standardized pattern expression above vs. below the 75% percentile of the respective diagnostic score. See also **eTable 7** for the respective mixed models' analysis results and **eTable 8** for BrainAGE-adjusted analysis results.

© 2022 Koutsouleris N et al. *JAMA Psychiatry*.

a

High vs. Low pattern expression	Clinical high-risk states for psychosis				
**Pattern**	**Sens**	**Spec**	**BAC**	**AUC**	P_{FDR}
bvFTD	**65.7**	**64.6**	**65.2**	**0.65**	**.008**
Schizophrenia	**63.6**	**72.2**	**67.9**	**0.73**	**.008**
Established AD	45.9	62.2	54.1	0.56	.263
MCI / Early-stage AD	48.6	58.6	53.6	0.58	.263
BrainAGE	42.9	63.2	53.0	0.56	.263
	**Recent-onset depression**				
**Pattern**	**Sens**	**Spec**	**BAC**	**AUC**	P_{FDR}
bvFTD	41.0	59.6	50.3	0.50	.676
Schizophrenia	55.0	58.3	56.7	0.57	.156
Established AD	30.6	58.0	44.3	0.46	.890
MCI / Early-stage AD	40.5	57.7	49.1	0.51	.676
BrainAGE	46.9	69.0	57.9	0.55	.156

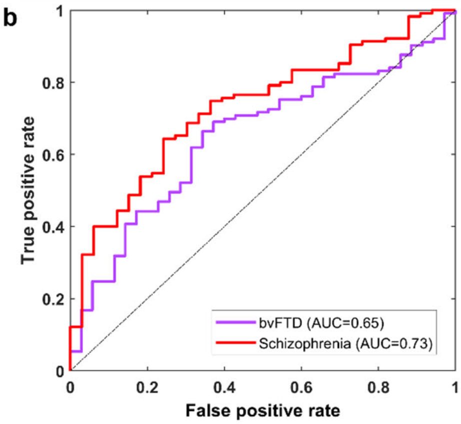

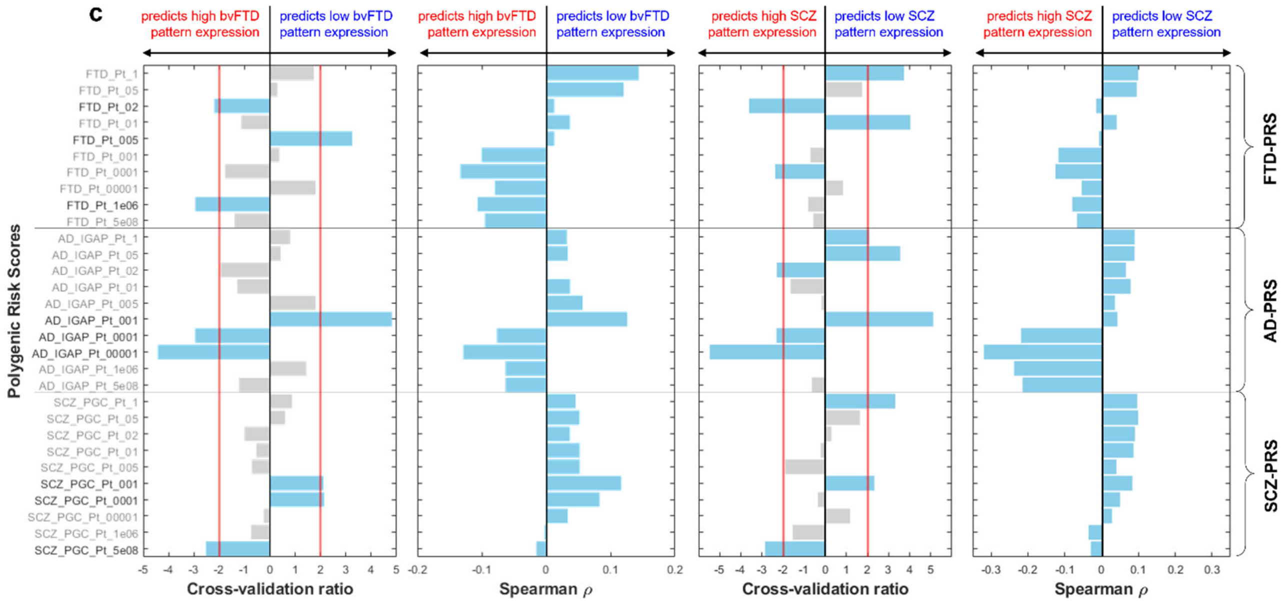

eFigure 23. Machine-learning based analysis of multivariate polygenic risk signatures informing a possible genetic discrimination of high vs. low bvFTD, schizophrenia (SCZ), established AD, MCI/early-stage AD and BrainAGE pattern groups in CHR or ROD patients. The Table (**a**) lists the discriminative performance of CHR- or ROD-specific models for the 5 different binary expression labels (*Sens* Sensitivity, *Spec* Specificity, *BAC* Balanced Accuracy, *AUC* Area-under-the-curve). Each of the 10 models was tested for significance using 1000 random label permutations (P_{FDR} Permutation-based *P* values corrected for multiple comparisons using FDR). After FDR correction, only the CHR-specific model separating high vs. low bvFTD and schizophrenia pattern expression subgroups remained significant and was further analyzed. The ROC plots (**b**) depict subject-level genetic classification results while the multivariate feature reliability (cross-validation ratio) and univariate association effects (Spearman ρ) are shown in **c**.

© 2022 Koutsouleris N et al. *JAMA Psychiatry*.

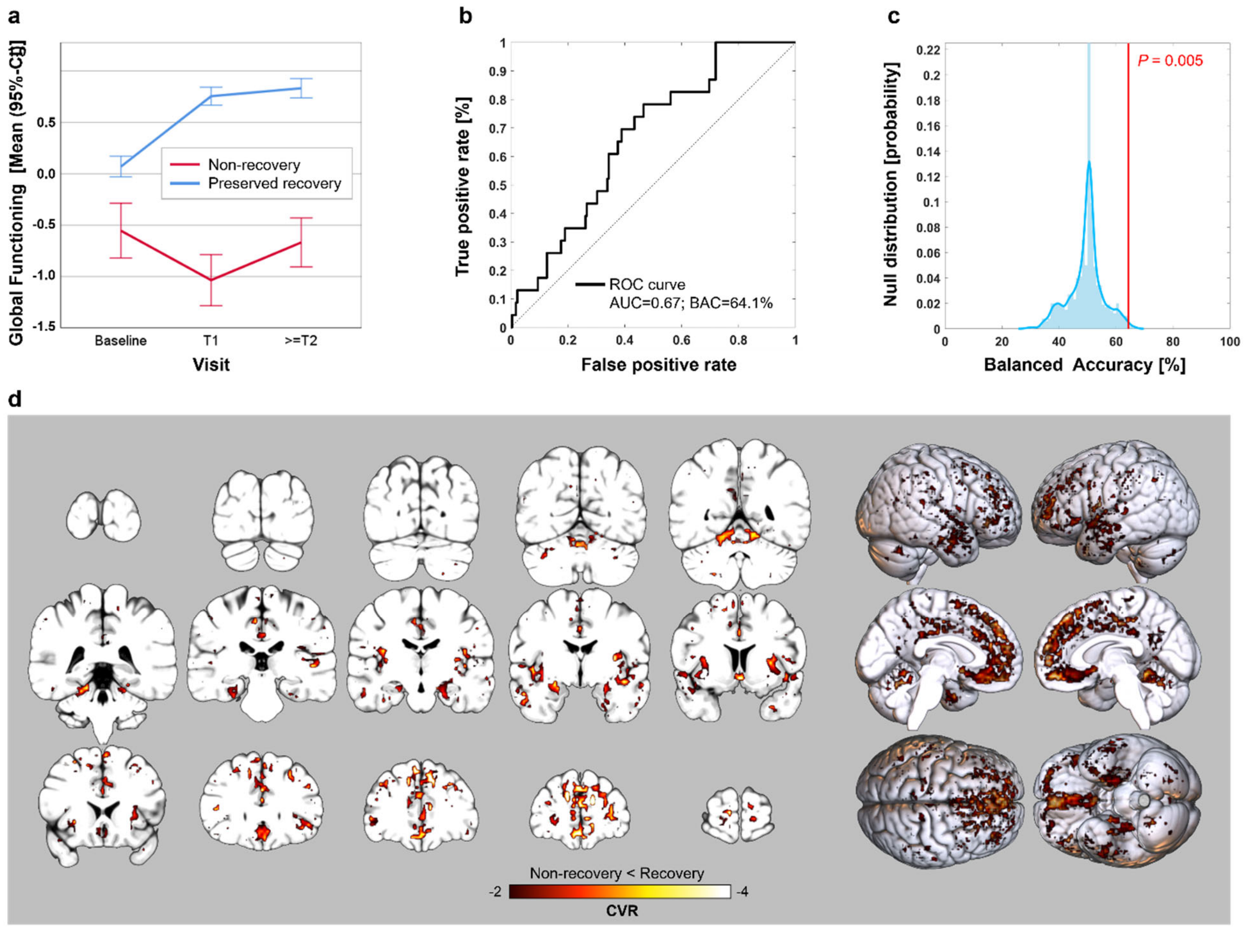

eFigure 24. The prognostic non-recovery classifier trained on PRONIA CHR and ROD patients. The figure shows **(a)** the mean (95% confidence interval) trajectories of PRONIA CHR and ROD patients with poor vs. good global functioning trajectories spanning on average (SD) 821.5 (270.6) days, **(b)** the receiver-operator curve (ROC) and are-under-the-curve (AUC) of the prognostic classifier differentiating between these two functioning courses, **(c)** the permutation-based significance analysis of the classifier, and **(d)** its neuroanatomical distribution as measured by cross-validation ratio mapping.

© 2022 Koutsouleris N et al. *JAMA Psychiatry*.

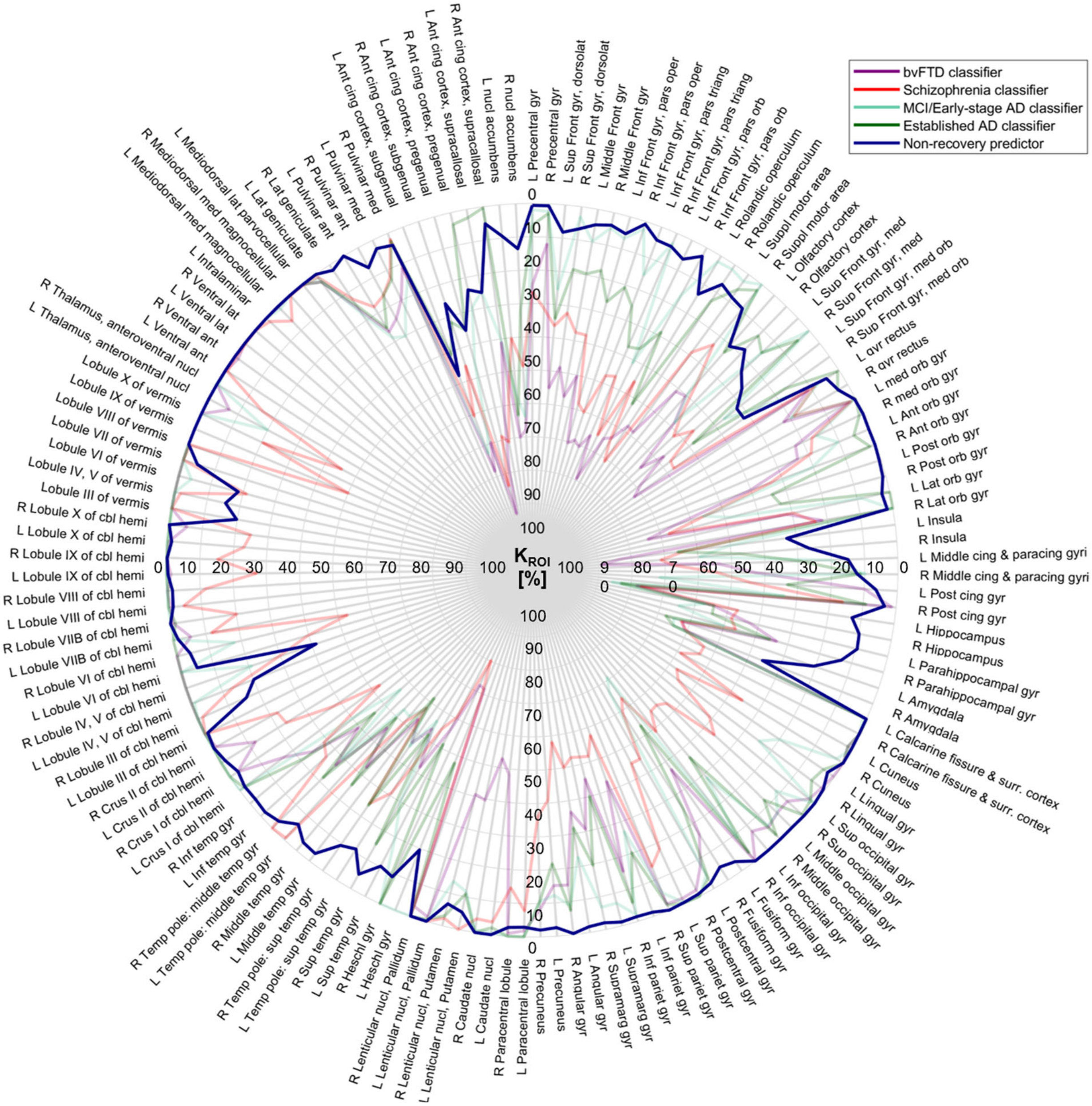

eFigure 25. Mapping of the prognostic non-recovery classifier's signature to the AAL3 atlas based on spatial extent. See legend of **eFigure 3** and Supplementary Methods for a description of the mapping procedure. The K_{ROI}% parcellations of the four diagnostic classifiers were added to the spider plot in transparent colors for comparison with the non-recovery predictor. **Abbreviations:** *Ant* Anterior, *Cbl* Cerebellum, *Cing* Cingulate, *Dorsolat* Dorsolateral, *Front* Frontal, *Gyr* Gyrus, *Hemi* Hemisphere, *Inf* Inferior, *Lat* Lateral, *Med* Medial, *Nucl* Nucleus, *Orb* Orbital, *Post* Posterior, *Sup* Superior, *Supramarg* Supramarginal, *Suppl* Supplementary, *Surr* Surrounding, *Temp* Temporal.

© 2022 Koutsouleris N et al. *JAMA Psychiatry*.

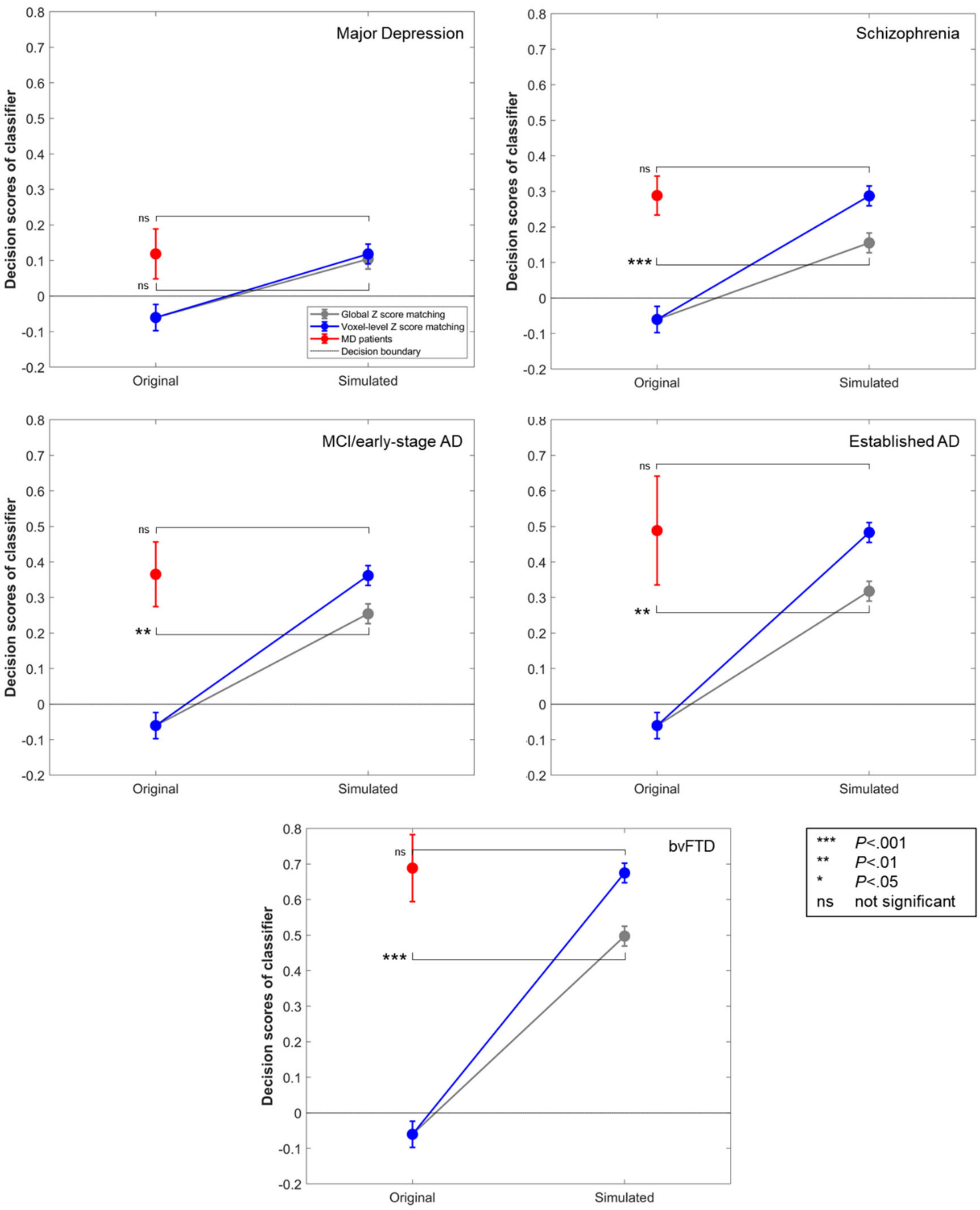

eFigure 26. Probing the spatial specificity of the prognostic non-recovery classifier by means of atrophy simulation. To test the prognostic non-recovery classifier for spatial specificity, the HC sample pooled across the FTLDc, Munich and OASIS-3 cohorts was used as described in the **Supplementary Methods** and **eFigure 11**. The null hypothesis of spatial non-specificity (grey lines) was created by calculating the mean difference between the HC group and the respective target patient group across all voxels in the inter-site reliability mask and subtracting this value from all voxels. For the alternative hypothesis (blue lines), the voxel-wise Z score difference image was computed between HC and target patient groups and subtracted from the HC sample. Two-sample t tests were conducted to compare simulated with observed group-level decision scores of the respective target patient group and corrected for multiple comparisons using FDR (q=0.05).

© 2022 Koutsouleris N et al. *JAMA Psychiatry*.

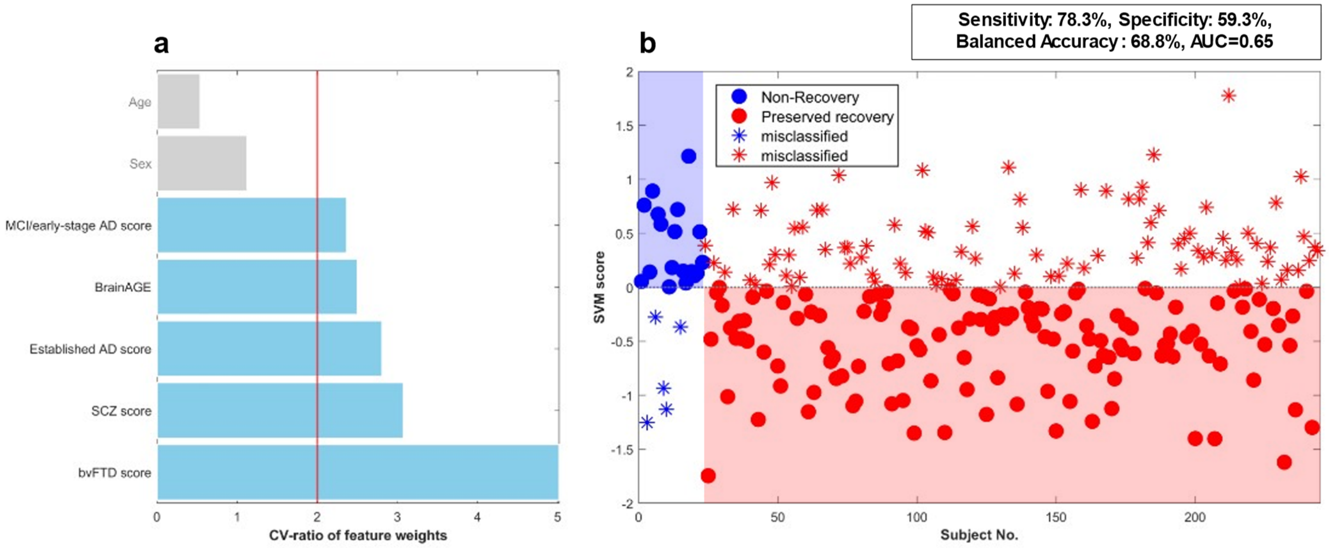

eFigure 27. Prognostic classification of CHR and ROD patients with functional non-recovery vs. preserved recovery using the diagnostic scores and BrainAGE estimates previously generated by the independent application of respective models to the PRONIA baseline data. (**a**) Feature reliability profile indicating that increased bvFTD pattern expression was the most relevant prognostic feature in identifying future non-recovery. (**b**) Classification plot showing correct (circles) and wrongly classified (stars) patients with functional non-recovery (blue) vs. recovery (red) as determined by nested 10-fold cross-validation.

© 2022 Koutsouleris N et al. *JAMA Psychiatry*.

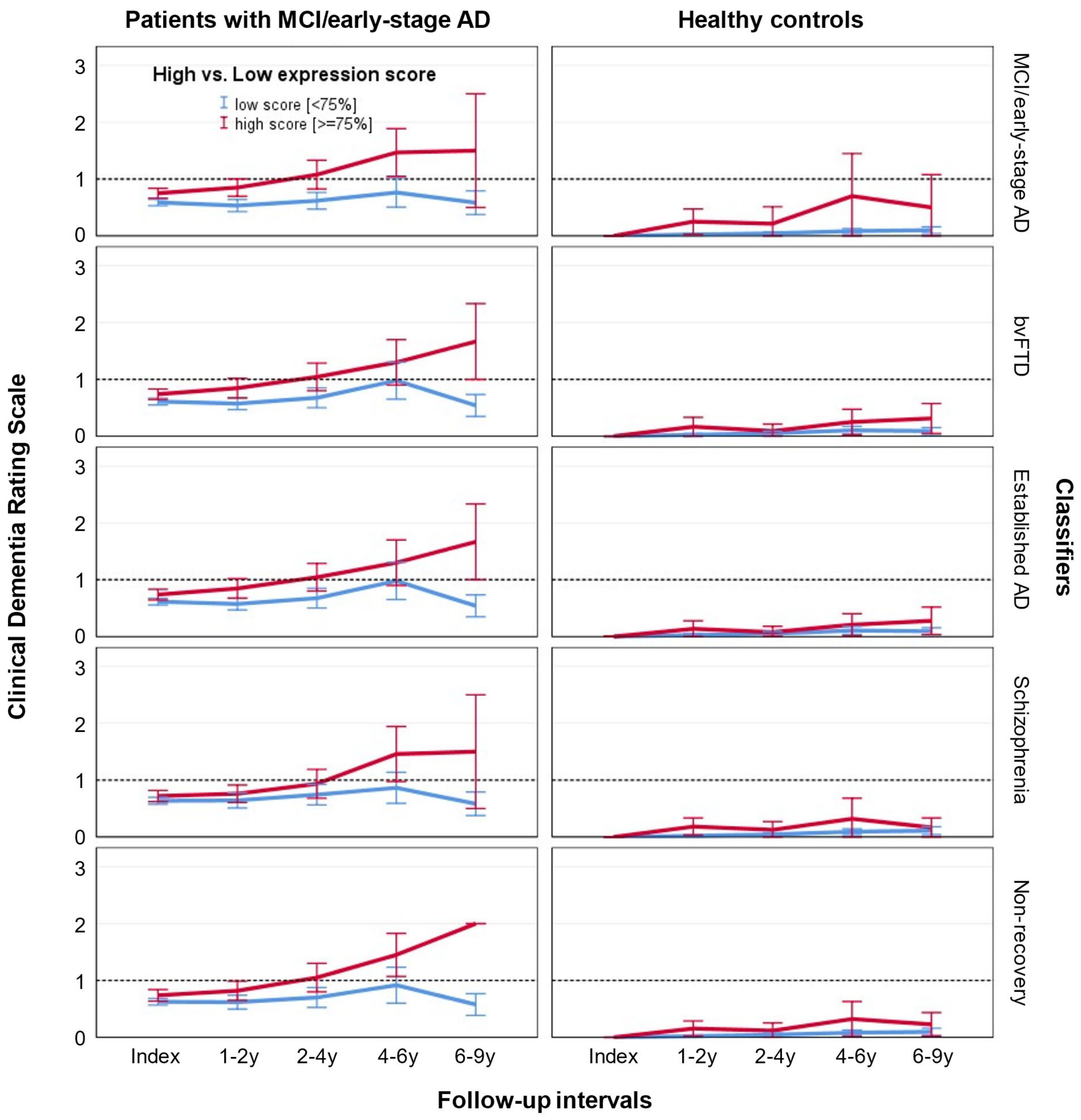

eFigure 28. Trajectory analysis covering a nine-years follow-up period of Clinical Dementia Rating scores in patients with MCI/early-stage AD (left) or healthy controls (right). Patients scored either high or low on the diagnostic case-control patterns or the prognostic non-recovery signature as defined by an upper quartile cutoff applied to the respective classifier's decision score distribution. See also **eTable 12** for a quantitative analysis using mixed-effects linear models.

© 2022 Koutsouleris N et al. *JAMA Psychiatry*.

eReferences.

1. Koutsouleris N, Meisenzahl EM, Borgwardt S, et al. Individualized differential diagnosis of schizophrenia and mood disorders using neuroanatomical biomarkers. *Brain*. 2015;138(Pt 7):2059-2073. doi:10.1093/brain/awv111

2. Otto M, Ludolph AC, Landwehrmeyer B, et al. [German consortium for frontotemporal lobar degeneration]. *Nervenarzt*. 2011;82(8):1002-1005. doi:10.1007/s00115-011-3261-3

3. Rascovsky K, Hodges JR, Knopman D, et al. Sensitivity of revised diagnostic criteria for the behavioural variant of frontotemporal dementia. *Brain J Neurol*. 2011;134(Pt 9):2456-2477. doi:10.1093/brain/awr179

4. American Psychiatric Association. *Diagnostic and Statistical Manual of Mental Disorders*. Fifth Edition. American Psychiatric Association; 2013. doi:10.1176/appi.books.9780890425596

5. Fillenbaum GG, van Belle G, Morris JC, et al. Consortium to Establish a Registry for Alzheimer's Disease (CERAD): the first twenty years. *Alzheimers Dement*. 2008;4(2):96-109.

6. Folstein MF, Folstein SE, McHugh PR. "Mini-mental state": a practical method for grading the cognitive state of patients for the clinician. *J Psychiatr Res*. 1975;12(3):189-198.

7. LaMontagne PJ, Benzinger TL, Morris JC, et al. OASIS-3: longitudinal neuroimaging, clinical, and cognitive dataset for normal aging and Alzheimer disease. *medRxiv*. Published online 2019.

8. Kay SR, Fiszbein A, Opler LA. The positive and negative syndrome scale (PANSS) for schizophrenia. *Schizophr Bull*. 1987;13(2):261-276.

9. HAMILTON M. A rating scale for depression. *J Neurol Neurosurg Psychiatry*. 1960;23:56-62.

10. Koutsouleris N, Dwyer DB, Degenhardt F, et al. Multimodal Machine Learning Workflows for Prediction of Psychosis in Patients With Clinical High-Risk Syndromes and Recent-Onset Depression. *JAMA Psychiatry*. Published online Dezember 2020. doi:10.1001/jamapsychiatry.2020.3604

11. Koutsouleris N, Kambeitz-Ilankovic L, Ruhrmann S, et al. Prediction Models of Functional Outcomes for Individuals in the Clinical High-Risk State for Psychosis or With Recent-Onset Depression: A Multimodal, Multisite Machine Learning Analysis. *JAMA Psychiatry*. 2018;75(11):1156-1172. doi:10.1001/jamapsychiatry.2018.2165

12. Pedersen G, Hagtvet KA, Karterud S. Generalizability studies of the Global Assessment of Functioning–Split version. *Compr Psychiatry*. 2007;48(1):88-94.

13. Llorca PM, Lançon C, Lancrenon S, et al. The "Functional Remission of General Schizophrenia"(FROGS) scale: development and validation of a new questionnaire. *Schizophr Res*. 2009;113(2):218-225.

14. Koutsouleris N, Dwyer DB, Degenhardt F, et al. Multimodal Machine Learning Workflows for Prediction of Psychosis in Patients With Clinical High-Risk Syndromes and Recent-Onset Depression. *JAMA Psychiatry*. 2021;78(2):195-209. doi:10.1001/jamapsychiatry.2020.3604

15. Brennan RL. Generalizability theory and classical test theory. *Appl Meas Educ*. 2010;24(1):1-21.

© 2022 Koutsouleris N et al. *JAMA Psychiatry*.

16. Kunkle BW, Grenier-Boley B, Sims R, et al. Genetic meta-analysis of diagnosed Alzheimer's disease identifies new risk loci and implicates Aβ, tau, immunity and lipid processing. *Nat Genet*. 2019;51(3):414-430. doi:10.1038/s41588-019-0358-2

17. Koutsouleris N, Kahn RS, Chekroud AM, et al. Multisite prediction of 4-week and 52-week treatment outcomes in patients with first-episode psychosis: a machine learning approach. *Lancet Psychiatry*. 2016;3(10):935-946. doi:10.1016/S2215-0366(16)30171-7

18. Filzmoser P, Liebmann B, Varmuza K. Repeated double cross validation. *J Chemom*. 2009;23:160-171.

19. Noble WS. What is a support vector machine? *Nat Biotechnol*. 2006;24(12):1565-1567. doi:10.1038/nbt1206-1565

20. Vapnik V. *The Nature of Statistical Learning Theory*. 2nd ed. Springer-Verlag; 2000.

21. Hansen LK, Larsen J, Nielsen FA, et al. Generalizable patterns in neuroimaging: how many principal components? *Neuroimage*. 1999;9(5):534-544. doi:10.1006/nimg.1998.0425

22. Chang CC, Lin CJ. *LIBSVM: A Library for Support Vector Machines*.; 2001.

23. Larner AJ. Number Needed to Diagnose, Predict, or Misdiagnose: Useful Metrics for Non-Canonical Signs of Cognitive Status? *Dement Geriatr Cogn Disord Extra*. 2018;8(3):321-327. doi:10.1159/000492783

24. Krishnan A, Williams LJ, McIntosh AR, Abdi H. Partial Least Squares (PLS) methods for neuroimaging: A tutorial and review. *Neuroimage*. Published online July 2010. doi:10.1016/j.neuroimage.2010.07.034

25. Gaonkar B, T Shinohara R, Davatzikos C, Initiative ADN. Interpreting support vector machine models for multivariate group wise analysis in neuroimaging. *Med Image Anal*. 2015;24(1):190-204. doi:10.1016/j.media.2015.06.008

26. Rolls ET, Huang CC, Lin CP, Feng J, Joliot M. Automated anatomical labelling atlas 3. *NeuroImage*. 2020;206:116189. doi:10.1016/j.neuroimage.2019.116189

27. Polikar R. Ensemble based systems in decision making. *IEEE Circuits Syst*. 2006;6(3):21-45. doi:10.1109/MCAS.2006.1688199

28. McNemar Q. Note on the sampling error of the difference between correlated proportions or percentages. *Psychometrika*. 1947;12(2):153-157. doi:10.1007/BF02295996

29. Sidak Z. Rectangular Confidence Regions for the Means of Multivariate Normal Distributions. *J Am Stat Assoc*. 1967;62(318):626. doi:10.1080/01621459.1967.10482935

30. Gaser C, Franke K, Klöppel S, Koutsouleris N, Sauer H, Alzheimer's Disease Neuroimaging Initiative. BrainAGE in Mild Cognitive Impaired Patients: Predicting the Conversion to Alzheimer's Disease. *PLoS One*. 2013;8(6):e67346.

© 2022 Koutsouleris N et al. *JAMA Psychiatry*.

31. Franke K, Ziegler G, Klöppel S, Gaser C, Alzheimer's Disease Neuroimaging Initiative. Estimating the age of healthy subjects from T1-weighted MRI scans using kernel methods: exploring the influence of various parameters. *Neuroimage*. 2010;50(3):883-892.

32. Kaufmann T, van der Meer D, Doan NT, et al. Common brain disorders are associated with heritable patterns of apparent aging of the brain. *Nat Neurosci*. 2019;22(10):1617-1623. doi:10.1038/s41593-019-0471-7

33. Koutsouleris N, Davatzikos C, Borgwardt S, et al. Accelerated brain aging in schizophrenia and beyond: a neuroanatomical marker of psychiatric disorders. *Schizophr Bull*. 2014;40(5):1140-1153.

34. Schnack HG, Van Haren NE, Nieuwenhuis M, Hulshoff Pol HE, Cahn W, Kahn RS. Accelerated brain aging in schizophrenia: a longitudinal pattern recognition study. *Am J Psychiatry*. 2016;173(6):607-616.

35. Schölkopf B, Smola A, Williamson RC, Bartlett PL. New support vector algorithms. *Neural Comput*. 2000;12:1207-1245.

36. Fortin JP, Cullen N, Sheline YI, et al. Harmonization of cortical thickness measurements across scanners and sites. *NeuroImage*. 2018;167:104-120. doi:10.1016/j.neuroimage.2017.11.024

37. Fortin JP, Parker D, Tunç B, et al. Harmonization of multi-site diffusion tensor imaging data. *NeuroImage*. 2017;161:149-170. doi:10.1016/j.neuroimage.2017.08.047

38. Beheshti I, Nugent S, Potvin O, Duchesne S. Bias-adjustment in neuroimaging-based brain age frameworks: A robust scheme. *NeuroImage Clin*. 2019;24:102063. doi:10.1016/j.nicl.2019.102063

39. Franke K, Gaser C. Ten Years of BrainAGE as a Neuroimaging Biomarker of Brain Aging: What Insights Have We Gained? *Front Neurol*. 2019;10:789. doi:10.3389/fneur.2019.00789

40. Devenney EM, Ahmed RM, Halliday G, Piguet O, Kiernan MC, Hodges JR. Psychiatric disorders in C9orf72 kindreds: Study of 1,414 family members. *Neurology*. 2018;91(16):e1498-e1507. doi:10.1212/WNL.0000000000006344

41. Watson A, Pribadi M, Chowdari K, et al. C9orf72 repeat expansions that cause frontotemporal dementia are detectable among patients with psychosis. *Psychiatry Res*. 2016;235:200-202. doi:10.1016/j.psychres.2015.12.007

42. Quade D. Using Weighted Rankings in the Analysis of Complete Blocks with Additive Block Effects. *J Am Stat Assoc*. 1979;74(367):680-683.

43. Heckert NA, Filliben JJ. *Dataplot Reference Manual, Volume 2: Let Subcommands and Library Functions.* National Institute of Standards and Technology; 2003.

44. Benjamini Y, Hochberg Y. Controlling the false discovery rate: a practical and powerful approach to multiple testing. *J R Stat Soc Ser B Methodol*. Published online 1995:289-300.

45. Golland P, Fischl B. Permutation tests for classification: towards statistical significance in image-based studies. *Inf Process Med Imaging*. 2003;18:330-341.

© 2022 Koutsouleris N et al. *JAMA Psychiatry*.
