# Supplementary material for: Exploring Links Between Psychosis and Frontotemporal Dementia Using Multimodal Machine Learning: Dementia Praecox Revisited
Source: JAMA Psychiatry. 2022 Aug 3;79(9):907–19. doi: 10.1001/jamapsychiatry.2022.2075 (PMC9350851; doi:10.1001/jamapsychiatry.2022.2075)
Supplement: Supplement 2. — International FTD-Genetics Consortium (IFGC), the German FTLD Consortium, and the PRONIA Consortium [file jamapsychiatry-e222075-s002.pdf]

Supplemental Online Content: Nonauthor Collaborators
*First name, last name, and suffix (if applicable) are required and will appear in PubMed.

*Group Name(s): The International FTD-Genetics Consortium (IFGC), the German Frontotemporal Lobar Degeneration (FTLD) Consortium, and the PRONIA Consortium							
***First Name and Middle Initial(s)**	***Last Name**	***Suffix (eg, Jr, III)**	Academic Degrees	Institution	Location (city, state/province, country)	Role or Contribution, eg, chair, principal investigator	Group (if more than 1 Group listed in the byline) and/or Subgroup (eg, Steering Committee)
Shalaila	Haas			Department of Psychiatry and Psychotherapy, Ludwig-Maximilian-University, Munich, Germany	Munich, Germany	Researcher	PRONIA
Alkomiet	Hasan			Department of Psychiatry and Psychotherapy, Ludwig-Maximilian-University, Munich, Germany	Munich, Germany	Researcher	PRONIA
Claudius	Hoff			Department of Psychiatry and Psychotherapy, Ludwig-Maximilian-University, Munich, Germany	Munich, Germany	Researcher	PRONIA
Ifrah	Khanyaree			Department of Psychiatry and Psychotherapy, Ludwig-Maximilian-University, Munich, Germany	Munich, Germany	Researcher	PRONIA
Aylin	Melo			Department of Psychiatry and Psychotherapy, Ludwig-Maximilian-University, Munich, Germany	Munich, Germany	Researcher	PRONIA
Susanna	Muckenhuber-Sternbauer			Department of Psychiatry and Psychotherapy, Ludwig-Maximilian-University, Munich, Germany	Munich, Germany	Researcher	PRONIA
Yanis	Köhler			Department of Psychiatry and Psychotherapy, Ludwig-Maximilian-University, Munich, Germany	Munich, Germany	Researcher	PRONIA
Ömer	Öztürk			Department of Psychiatry and Psychotherapy, Ludwig-Maximilian-University, Munich, Germany	Munich, Germany	Researcher	PRONIA
Nora	Penzel			Department of Psychiatry and Psychotherapy, Ludwig-Maximilian-University, Munich, Germany	Munich, Germany	Researcher	PRONIA

*First name, last name, and suffix (if applicable) are required and will appear in PubMed.

*First Name and Middle Initial(s)	*Last Name	*Suffix (eg, Jr, III)	Academic Degrees	Institution	Location (city, state/province, country)	Role or Contribution, eg, chair, principal investigator	Group (if more than 1 Group listed in the byline) and/or Subgroup (eg, Steering Committee)
Adrian	Rangnick			Department of Psychiatry and Psychotherapy, Ludwig-Maximilian-University, Munich, Germany	Munich, Germany	Researcher	PRONIA
Sebastian	von Saldern			Department of Psychiatry and Psychotherapy, Ludwig-Maximilian-University, Munich, Germany	Munich, Germany	Researcher	PRONIA
Moritz	Spangemacher			Department of Psychiatry and Psychotherapy, Ludwig-Maximilian-University, Munich, Germany	Munich, Germany	Researcher	PRONIA
Ana	Tupac			Department of Psychiatry and Psychotherapy, Ludwig-Maximilian-University, Munich, Germany	Munich, Germany	Researcher	PRONIA
Johanna	Weiske			Department of Psychiatry and Psychotherapy, Ludwig-Maximilian-University, Munich, Germany	Munich, Germany	Researcher	PRONIA
Antonia	Wosgien			Department of Psychiatry and Psychotherapy, Ludwig-Maximilian-University, Munich, Germany	Munich, Germany	Researcher	PRONIA
Camilla	Krämer			Department of Psychiatry and Psychotherapy, Ludwig-Maximilian-University, Munich, Germany	Munich, Germany	Researcher	PRONIA
Karsten	Blume			Department of Psychiatry and Psychotherapy, University of Cologne, Cologne, Germany	Cologne, Germany	Researcher	PRONIA
Dennis	Hedderich			Department of Psychiatry and Psychotherapy, University of Cologne, Cologne, Germany	Cologne, Germany	Researcher	PRONIA
Dominika	Julkowski			Department of Psychiatry and Psychotherapy, University of Cologne, Cologne, Germany	Cologne, Germany	Researcher	PRONIA

Supplemental Online Content: Nonauthor Collaborators
*First name, last name, and suffix (if applicable) are required and will appear in PubMed.

***First Name and Middle Initial(s)**	***Last Name**	***Suffix (eg, Jr, III)**	Academic Degrees	Institution	Location (city, state/province, country)	Role or Contribution, eg, chair, principal investigator	Group (if more than 1 Group listed in the byline) and/or Subgroup (eg, Steering Committee)
Nathalie	Kaiser			Department of Psychiatry and Psychotherapy, University of Cologne, Cologne, Germany	Cologne, Germany	Researcher	PRONIA
Thorsten	Lichtenstein			Department of Psychiatry and Psychotherapy, University of Cologne, Cologne, Germany	Cologne, Germany	Researcher	PRONIA
Ruth	Milz			Department of Psychiatry and Psychotherapy, University of Cologne, Cologne, Germany	Cologne, Germany	Researcher	PRONIA
Alexandra	Nikolaides			Department of Psychiatry and Psychotherapy, University of Cologne, Cologne, Germany	Cologne, Germany	Researcher	PRONIA
Tanja	Pilgram			Department of Psychiatry and Psychotherapy, University of Cologne, Cologne, Germany	Cologne, Germany	Researcher	PRONIA
Mauro	Seves			Department of Psychiatry and Psychotherapy, University of Cologne, Cologne, Germany	Cologne, Germany	Researcher	PRONIA
Martina	Wassen			Department of Psychiatry and Psychotherapy, University of Cologne, Cologne, Germany	Cologne, Germany	Researcher	PRONIA
Christina	Andreou			Department of Psychiatry (Psychiatric University Hospital, UPK), University of Basel, Switzerland	Basel, Switzerland	Researcher	PRONIA
Laura	Egloff			Department of Psychiatry (Psychiatric University Hospital, UPK), University of Basel, Switzerland	Basel, Switzerland	Researcher	PRONIA
Fabienne	Harrisberger			Department of Psychiatry (Psychiatric University Hospital, UPK), University of Basel, Switzerland	Basel, Switzerland	Researcher	PRONIA

*First name, last name, and suffix (if applicable) are required and will appear in PubMed.

***First Name and Middle Initial(s)**	***Last Name**	***Suffix (eg, Jr, III)**	Academic Degrees	Institution	Location (city, state/province, country)	Role or Contribution, eg, chair, principal investigator	Group (if more than 1 Group listed in the byline) and/or Subgroup (eg, Steering Committee)
Ulrike	Heitz			Department of Psychiatry (Psychiatric University Hospital, UPK), University of Basel, Switzerland	Basel, Switzerland	Researcher	PRONIA
Claudia	Lenz			Department of Psychiatry (Psychiatric University Hospital, UPK), University of Basel, Switzerland	Basel, Switzerland	Researcher	PRONIA
Letizia	Leanza			Department of Psychiatry (Psychiatric University Hospital, UPK), University of Basel, Switzerland	Basel, Switzerland	Researcher	PRONIA
Amatya	Mackintosh			Department of Psychiatry (Psychiatric University Hospital, UPK), University of Basel, Switzerland	Basel, Switzerland	Researcher	PRONIA
Renata	Smieskova			Department of Psychiatry (Psychiatric University Hospital, UPK), University of Basel, Switzerland	Basel, Switzerland	Researcher	PRONIA
Erich	Studerus			Department of Psychiatry (Psychiatric University Hospital, UPK), University of Basel, Switzerland	Basel, Switzerland	Researcher	PRONIA
Anna	Walter			Department of Psychiatry (Psychiatric University Hospital, UPK), University of Basel, Switzerland	Basel, Switzerland	Researcher	PRONIA
Sonja	Widmayer			Department of Psychiatry (Psychiatric University Hospital, UPK), University of Basel, Switzerland	Basel, Switzerland	Researcher	PRONIA
Chris	Day			Institute of Mental Health & School of Psychology, University of Birmingham, United Kingdom	Birmingham, United Kingdom	Researcher	PRONIA
Sian	Lowri Griffiths			Institute of Mental Health & School of Psychology, University of Birmingham, United Kingdom	Birmingham, United Kingdom	Researcher	PRONIA

*First name, last name, and suffix (if applicable) are required and will appear in PubMed.

***First Name and Middle Initial(s)**	***Last Name**	***Suffix (eg, Jr, III)**	Academic Degrees	Institution	Location (city, state/province, country)	Role or Contribution, eg, chair, principal investigator	Group (if more than 1 Group listed in the byline) and/or Subgroup (eg, Steering Committee)
Mariam	Iqbal			Institute of Mental Health & School of Psychology, University of Birmingham, United Kingdom	Birmingham, United Kingdom	Researcher	PRONIA
Mirabel	Pelton			Institute of Mental Health & School of Psychology, University of Birmingham, United Kingdom	Birmingham, United Kingdom	Researcher	PRONIA
Pavan	Mallikarjun			Institute of Mental Health & School of Psychology, University of Birmingham, United Kingdom	Birmingham, United Kingdom	Researcher	PRONIA
Alexandra	Stainton			Institute of Mental Health & School of Psychology, University of Birmingham, United Kingdom	Birmingham, United Kingdom	Researcher	PRONIA
Ashleigh	Lin			Institute of Mental Health & School of Psychology, University of Birmingham, United Kingdom	Birmingham, United Kingdom	Researcher	PRONIA
Paris	Lalousis			Institute of Mental Health & School of Psychology, University of Birmingham, United Kingdom	Birmingham, United Kingdom	Researcher	PRONIA
Alexander	Denissoff			Department of Psychiatry, University of Turku, Finland	Turku, Finland	Researcher	PRONIA
Anu	Ellilä			Department of Psychiatry, University of Turku, Finland	Turku, Finland	Researcher	PRONIA
Tiina	From			Department of Psychiatry, University of Turku, Finland	Turku, Finland	Researcher	PRONIA
Markus	Heinimaa			Department of Psychiatry, University of Turku, Finland	Turku, Finland	Researcher	PRONIA
Tuula	Ilonen			Department of Psychiatry, University of Turku, Finland	Turku, Finland	Researcher	PRONIA
Päivi	Jalo			Department of Psychiatry, University of Turku, Finland	Turku, Finland	Researcher	PRONIA

*First name, last name, and suffix (if applicable) are required and will appear in PubMed.

***First Name and Middle Initial(s)**	***Last Name**	***Suffix (eg, Jr, III)**	Academic Degrees	Institution	Location (city, state/province, country)	Role or Contribution, eg, chair, principal investigator	Group (if more than 1 Group listed in the byline) and/or Subgroup (eg, Steering Committee)
Heikki	Laurikainen			Department of Psychiatry, University of Turku, Finland	Turku, Finland	Researcher	PRONIA
Antti	Luutonen			Department of Psychiatry, University of Turku, Finland	Turku, Finland	Researcher	PRONIA
Akseli	Mäkela			Department of Psychiatry, University of Turku, Finland	Turku, Finland	Researcher	PRONIA
Janina	Paju			Department of Psychiatry, University of Turku, Finland	Turku, Finland	Researcher	PRONIA
Henri	Pesonen			Department of Psychiatry, University of Turku, Finland	Turku, Finland	Researcher	PRONIA
Reetta-Liina	Säilä			Department of Psychiatry, University of Turku, Finland	Turku, Finland	Researcher	PRONIA
Anna	Toivonen			Department of Psychiatry, University of Turku, Finland	Turku, Finland	Researcher	PRONIA
Otto	Turtonen			Department of Psychiatry, University of Turku, Finland	Turku, Finland	Researcher	PRONIA
Sonja	Botterweck			Department of Psychiatry (Psychiatric University Hospital LVR/HHU Düsseldorf), University of Düsseldorf, Germany	Düsseldorf, Germany	Researcher	PRONIA
Norman	Kluthausen			Department of Psychiatry (Psychiatric University Hospital LVR/HHU Düsseldorf), University of Düsseldorf, Germany	Düsseldorf, Germany	Researcher	PRONIA
Gerald	Antoch			Department of Psychiatry (Psychiatric University Hospital LVR/HHU Düsseldorf), University of Düsseldorf, Germany	Düsseldorf, Germany	Researcher	PRONIA

***First Name and Middle Initial(s)**	***Last Name**	***Suffix (eg, Jr, III)**	Academic Degrees	Institution	Location (city, state/province, country)	Role or Contribution, eg, chair, principal investigator	Group (if more than 1 Group listed in the byline) and/or Subgroup (eg, Steering Committee)
Julian	Caspers			Department of Psychiatry (Psychiatric University Hospital LVR/HHU Düsseldorf), University of Düsseldorf, Germany	Düsseldorf, Germany	Researcher	PRONIA
Hans-Jörg	Wittsack			Department of Psychiatry (Psychiatric University Hospital LVR/HHU Düsseldorf), University of Düsseldorf, Germany	Düsseldorf, Germany	Researcher	PRONIA
Giuseppe	Blasi			Department of Basic Medical Science, Neuroscience and Sense Organs - University of Bari Aldo Moro	Bari, Italy	Researcher	PRONIA
Giulio	Pergola			Department of Basic Medical Science, Neuroscience and Sense Organs - University of Bari Aldo Moro	Bari, Italy	Researcher	PRONIA
Grazia	Caforio			Department of Basic Medical Science, Neuroscience and Sense Organs - University of Bari Aldo Moro	Bari, Italy	Researcher	PRONIA
Leonardo	Fazio			Department of Basic Medical Science, Neuroscience and Sense Organs - University of Bari Aldo Moro	Bari, Italy	Researcher	PRONIA
Tiziana	Quarto			Department of Basic Medical Science, Neuroscience and Sense Organs - University of Bari Aldo Moro	Bari, Italy	Researcher	PRONIA
Barbara	Gelao			Department of Basic Medical Science, Neuroscience and Sense Organs - University of Bari Aldo Moro	Bari, Italy	Researcher	PRONIA
Raffaella	Romano			Department of Basic Medical Science, Neuroscience and Sense Organs - University of Bari Aldo Moro	Bari, Italy	Researcher	PRONIA

*First name, last name, and suffix (if applicable) are required and will appear in PubMed.

***First Name and Middle Initial(s)**	***Last Name**	***Suffix (eg, Jr, III)**	Academic Degrees	Institution	Location (city, state/province, country)	Role or Contribution, eg, chair, principal investigator	Group (if more than 1 Group listed in the byline) and/or Subgroup (eg, Steering Committee)
Ileana	Andriola			Department of Basic Medical Science, Neuroscience and Sense Organs - University of Bari Aldo Moro	Bari, Italy	Researcher	PRONIA
Andrea	Falsetti			Department of Basic Medical Science, Neuroscience and Sense Organs - University of Bari Aldo Moro	Bari, Italy	Researcher	PRONIA
Marina	Barone			Department of Basic Medical Science, Neuroscience and Sense Organs - University of Bari Aldo Moro	Bari, Italy	Researcher	PRONIA
Roberta	Passiatore			Department of Basic Medical Science, Neuroscience and Sense Organs - University of Bari Aldo Moro	Bari, Italy	Researcher	PRONIA
Marina	Sangiuliano			Department of Basic Medical Science, Neuroscience and Sense Organs - University of Bari Aldo Moro	Bari, Italy	Researcher	PRONIA
Marian	Surmann			Department of Psychiatry and Psychotherapy of the University of Münster, Germany	Münster, Germany	Researcher	PRONIA
Olga	Bienek			Department of Psychiatry and Psychotherapy of the University of Münster, Germany	Münster, Germany	Researcher	PRONIA
Udo	Dannlowski			Department of Psychiatry and Psychotherapy of the University of Münster, Germany	Münster, Germany	Researcher	PRONIA
Ana Beatriz	Solana			General Electric Global Research Inc., USA	Munich, Germany	Researcher	PRONIA
Manuela	Abraham			General Electric Global Research Inc., USA	Munich, Germany	Researcher	PRONIA
Timo	Schirmer			General Electric Global Research Inc., USA	Munich, Germany	Researcher	PRONIA

*First Name and Middle Initial(s)	*Last Name	*Suffix (eg, Jr, III)	Academic Degrees	Institution	Location (city, state/province, country)	Role or Contribution, eg, chair, principal investigator	Group (if more than 1 Group listed in the byline) and/or Subgroup (eg, Steering Committee)
Adele	Ferro			University of Milan, Italy	Milan, Italy	Researcher	PRONIA
Marta	Re			University of Milan, Italy	Milan, Italy	Researcher	PRONIA
Maurizio	Sberna			University of Milan, Italy	Milan, Italy	Researcher	PRONIA
Armando	D’Agostino			University of Milan, Italy	Milan, Italy	Researcher	PRONIA
Lorenzo	Del Fabro			University of Milan, Italy	Milan, Italy	Researcher	PRONIA
Giampaolo	Perna			University of Milan, Italy	Milan, Italy	Researcher	PRONIA
Maria	Nobile			University of Milan, Italy	Milan, Italy	Researcher	PRONIA
Matteo	Balestrieri			University of Udine, Italy	Udine, Italy	Researcher	PRONIA
Carolina	Bonivento			University of Udine, Italy	Udine, Italy	Researcher	PRONIA
Giuseppe	Cabras			University of Udine, Italy	Udine, Italy	Researcher	PRONIA
Franco	Fabbro			University of Udine, Italy	Udine, Italy	Researcher	PRONIA
Giuseppe	Delvecchio			IRCCS Policlinico of Milan, Italy	Milan, Italy	Researcher	PRONIA
Eleonora	Maggioni			IRCCS Policlinico of Milan, Italy	Milan, Italy	Researcher	PRONIA
Letizia	Squarcina			University of Milan, Italy	Milan, Italy	Researcher	PRONIA
Davide	Gritti			IRCCS Policlinico of Milan, Italy	Milan, Italy	Researcher	PRONIA
Maria Gloria	Rossetti			IRCCS Policlinico of Milan	Milan, Italy	Researcher	PRONIA
Raffaele	Ferrari			University College London, Department of Molecular Neuroscience	London, UK	Researcher	IFGC
Dena	Hernandez			Laboratory of Neurogenetics, National Institute on Aging, National Institutes of Health	Bethesda, USA	Researcher	IFGC
Michael	Nalls			Laboratory of Neurogenetics, National Institute on Aging, National Institutes of Health	Bethesda, USA	Researcher	IFGC
Jonathan	Rohrer			Reta Lila Weston Research Laboratories, Department of Molecular Neuroscience, UCL Institute of Neurology	London, UK	Researcher	IFGC
Adaikalavan	Ramasamy			Reta Lila Weston Research Laboratories, Department of Molecular Neuroscience, UCL Institute of Neurology	London, UK	Researcher	IFGC

Supplemental Online Content: Nonauthor Collaborators
*First name, last name, and suffix (if applicable) are required and will appear in PubMed.

***First Name and Middle Initial(s)**	***Last Name**	***Suffix (eg, Jr, III)**	Academic Degrees	Institution	Location (city, state/province, country)	Role or Contribution, eg, chair, principal investigator	Group (if more than 1 Group listed in the byline) and/or Subgroup (eg, Steering Committee)
John	Kwok			Neuroscience Research Australia	Sydney, Australia	Researcher	IFGC
Carol	Dobson-Stone			Neuroscience Research Australia	Sydney, Australia	Researcher	IFGC
William	Brooks			Neuroscience Research Australia	Sydney, Australia	Researcher	IFGC
Peter	Schofield			Neuroscience Research Australia	Sydney, Australia	Researcher	IFGC
Glenda	Halliday			Neuroscience Research Australia	Sydney, Australia	Researcher	IFGC
John	Hodges			Neuroscience Research Australia	Sydney, Australia	Researcher	IFGC
Olivier	Piguet			Neuroscience Research Australia	Sydney, Australia	Researcher	IFGC
Lauren	Bartley			Neuroscience Research Australia	Sydney, Australia	Researcher	IFGC
Elizabeth	Thompson			South Australian Clinical Genetics Service, SA Pathology	North Adelaide, Australia	Researcher	IFGC
Isabel	Hernández			Research Center and Memory Clinic of Fundació ACE, Institut Català de Neurociències Aplicades	Barcelona, Spain	Researcher	IFGC
Agustín	Ruiz			Research Center and Memory Clinic of Fundació ACE, Institut Català de Neurociències Aplicades	Barcelona, Spain	Researcher	IFGC
Mercè	Boada			Research Center and Memory Clinic of Fundació ACE, Institut Català de Neurociències Aplicades	Barcelona, Spain	Researcher	IFGC
Barbara	Borroni			Neurology Clinic, University of Brescia	Brescia, Italy	Researcher	IFGC
Alessandro	Padovani			Neurology Clinic, University of Brescia	Brescia, Italy	Researcher	IFGC
Carlos	Cruchaga			Department of Psychiatry, Washington University	St. Louis, MO, USA	Researcher	IFGC
Nigel	Cairns			Hope Center, Washington University School of Medicine	St. Louis, MO, USA	Researcher	IFGC
Luisa	Benussi			Molecular Markers Laboratory, IRCCS Istituto Centro San Giovanni di Dio Fatebenefratelli	Brescia, Italy	Researcher	IFGC

*First name, last name, and suffix (if applicable) are required and will appear in PubMed.

***First Name and Middle Initial(s)**	***Last Name**	***Suffix (eg, Jr, III)**	Academic Degrees	Institution	Location (city, state/province, country)	Role or Contribution, eg, chair, principal investigator	Group (if more than 1 Group listed in the byline) and/or Subgroup (eg, Steering Committee)
Giuliano	Binetti			MAC Memory Clinic, IRCCS Istituto Centro San Giovanni di Dio Fatebenefratelli	Brescia, Italy	Researcher	IFGC
Roberta	Ghidoni			Molecular Markers Laboratory, IRCCS Istituto Centro San Giovanni di Dio Fatebenefratelli	Brescia, Italy	Researcher	IFGC
Gianluigi	Forloni			Biology of Neurodegenerative Disorders, IRCCS Istituto di Ricerche Farmacologiche, "Mario Negri"	Milan, Italy	Researcher	IFGC
Diego	Albani			Biology of Neurodegenerative Disorders, IRCCS Istituto di Ricerche Farmacologiche, "Mario Negri"	Milan, Italy	Researcher	IFGC
Daniela	Galimberti			Fondazione Cà Granda, IRCCS Ospedale Maggiore Policlinico; University of Milan	Milan, Italy	Researcher	IFGC
Chiara	Fenoglio			Fondazione Cà Granda, IRCCS Ospedale Maggiore Policlinico; University of Milan	Milan, Italy	Researcher	IFGC
Maria	Serpente			Fondazione Cà Granda, IRCCS Ospedale Maggiore Policlinico; University of Milan	Milan, Italy	Researcher	IFGC
Elio	Scarpini			Fondazione Cà Granda, IRCCS Ospedale Maggiore Policlinico; University of Milan	Milan, Italy	Researcher	IFGC
Jordi	Clarimón			Memory Unit, Neurology Department and Sant Pau Biomedical Research Institute, Hospital de la Santa Creu i Sant Pau, Universitat Autònoma de Barcelona	Barcelona, Spain	Researcher	IFGC
Alberto	Lleó			Memory Unit, Neurology Department and Sant Pau Biomedical Research Institute, Hospital de la Santa Creu i Sant Pau, Universitat Autònoma de Barcelona	Barcelona, Spain	Researcher	IFGC

*First name, last name, and suffix (if applicable) are required and will appear in PubMed.

***First Name and Middle Initial(s)**	***Last Name**	***Suffix (eg, Jr, III)**	Academic Degrees	Institution	Location (city, state/province, country)	Role or Contribution, eg, chair, principal investigator	Group (if more than 1 Group listed in the byline) and/or Subgroup (eg, Steering Committee)
Rafael	Blesa			Memory Unit, Neurology Department and Sant Pau Biomedical Research Institute, Hospital de la Santa Creu i Sant Pau, Universitat Autònoma de Barcelona	Barcelona, Spain	Researcher	IFGC
Maria	Landqvist Waldö			Unit of Geriatric Psychiatry, Department of Clinical Sciences, Lund University	Lund, Sweden	Researcher	IFGC
Karin	Nilsson			Unit of Geriatric Psychiatry, Department of Clinical Sciences, Lund University	Lund, Sweden	Researcher	IFGC
Christer	Nilsson			Clinical Memory Research Unit, Department of Clinical Sciences, Lund University	Lund, Sweden	Researcher	IFGC
Ian	Mackenzie			Department of Pathology and Laboratory Medicine, University of British Columbia	Vancouver, Canada	Researcher	IFGC
Ging-Yuek	Hsiung			Division of Neurology, University of British Columbia	Vancouver, Canada	Researcher	IFGC
David	Mann			Institute of Brain, Behaviour and Mental Health, University of Manchester, Salford Royal Hospital	Salford, UK	Researcher	IFGC
Jordan	Grafman			Rehabilitation Institute of Chicago, Departments of Physical Medicine and Rehabilitation, Psychiatry, and Cognitive Neurology & Alzheimer's Disease Center; Feinberg School of Medicine, Northwestern University	Chicago, USA	Researcher	IFGC
Christopher	Morris			Newcastle Brain Tissue Resource, Institute for Ageing, Newcastle University	Newcastle upon Tyne, UK	Researcher	IFGC

Supplemental Online Content: Nonauthor Collaborators
*First name, last name, and suffix (if applicable) are required and will appear in PubMed.

*First Name and Middle Initial(s)	*Last Name	*Suffix (eg, Jr, III)	Academic Degrees	Institution	Location (city, state/province, country)	Role or Contribution, eg, chair, principal investigator	Group (if more than 1 Group listed in the byline) and/or Subgroup (eg, Steering Committee)
Johannes	Attems			Newcastle Brain Tissue Resource, Institute for Ageing, Newcastle University	Newcastle upon Tyne, UK	Researcher	IFGC
Ian	McKeith			Newcastle University, Institute of Neuroscience and Institute for Ageing, Campus for Ageing and Vitality, Newcastle University	Newcastle upon Tyne, UK	Researcher	IFGC
Alan	Thomas			Newcastle University, Institute of Neuroscience and Institute for Ageing, Campus for Ageing and Vitality, Newcastle University	Newcastle upon Tyne, UK	Researcher	IFGC
Pietro	Pietrini			IMT School for Advanced Studies	Lucca, Italy	Researcher	IFGC
Edward	Huey			Taub Institute, Departments of Psychiatry and Neurology, Columbia University	New York, USA	Researcher	IFGC
Eric	Wassermann			Behavioral Neurology Unit, National Inisitute of Neurological Disorders and Stroke, National Insititutes of Health	Bethesda, USA	Researcher	IFGC
Atik	Baborie			Department of Laboratory Medicine & Pathology, Walter Mackenzie Health Sciences Centre, University of Alberta Edmonton	Alberta, Canada	Researcher	IFGC
Evelyn	Jaros			Newcastle University, Institute for Ageing and Health, Campus for Ageing and Vitality	Newcastle upon Tyne, UK	Researcher	IFGC
Michael	Tierney			Behavioral Neurology Unit, National Inisitute of Neurological Disorders and Stroke, National Insititutes of Health	Bethesda, USA	Researcher	IFGC

*First name, last name, and suffix (if applicable) are required and will appear in PubMed.

***First Name and Middle Initial(s)**	***Last Name**	***Suffix (eg, Jr, III)**	Academic Degrees	Institution	Location (city, state/province, country)	Role or Contribution, eg, chair, principal investigator	Group (if more than 1 Group listed in the byline) and/or Subgroup (eg, Steering Committee)
Pau	Pastor			Center for Networker Biomedical Research in Neurodegenerative Diseases (CIBERNED)	Madrid, Spain	Researcher	IFGC
Cristina	Razquin			Neurogenetics Laboratory, Division of Neurosciences, Center for Applied Medical Research, Universidad de Navarra	Pamplona, Spain	Researcher	IFGC
Sara	Ortega-Cubero			Center for Networker Biomedical Research in Neurodegenerative Diseases (CIBERNED)	Madrid, Spain	Researcher	IFGC
Elena	Alonso			Neurogenetics Laboratory, Division of Neurosciences, Center for Applied Medical Research, Universidad de Navarra	Pamplona, Spain	Researcher	IFGC
Robert	Perneczky			Neuroepidemiology and Ageing Research Unit, School of Public Health, Faculty of Medicine, The Imperial College of Science, Technology and Medicine	London, UK	Researcher	IFGC
Panagiotis	Alexopoulos			Department of Psychiatry and Psychotherapy, Technische Universität München	Munich, Germany	Researcher	IFGC
Alexander	Kurz			Department of Psychiatry and Psychotherapy, Technische Universität München	Munich, Germany	Researcher	IFGC
Innocenzo	Rainero			Neurology I, Department of Neuroscience, University of Torino, Italy, A.O. Città della Salute e della Scienza di Torino	Torino, Italy	Researcher	IFGC

Supplemental Online Content: Nonauthor Collaborators
*First name, last name, and suffix (if applicable) are required and will appear in PubMed.

***First Name and Middle Initial(s)**	***Last Name**	***Suffix (eg, Jr, III)**	Academic Degrees	Institution	Location (city, state/province, country)	Role or Contribution, eg, chair, principal investigator	Group (if more than 1 Group listed in the byline) and/or Subgroup (eg, Steering Committee)
Elisa	Rubino			Neurology I, Department of Neuroscience, University of Torino, Italy, A.O. Città della Salute e della Scienza di Torino	Torino, Italy	Researcher	IFGC
Lorenzo	Pinessi			Neurology I, Department of Neuroscience, University of Torino, Italy, A.O. Città della Salute e della Scienza di Torino	Torino, Italy	Researcher	IFGC
Ekaterina	Rogaeva			Tanz Centre for Research in Neurodegenerative Diseases, University of Toronto	Toronto, Ontario, Canada	Researcher	IFGC
Peter	George-Hyslop			Tanz Centre for Research in Neurodegenerative Diseases, University of Toronto	Toronto, Ontario, Canada	Researcher	IFGC
Giacomina	Rossi			Division of Neurology V and Neuropathology, Fondazione IRCCS Istituto Neurologico Carlo Besta	Milan, Italy	Researcher	IFGC
Fabrizio	Tagliavini			Division of Neurology V and Neuropathology, Fondazione IRCCS Istituto Neurologico Carlo Besta	Milan, Italy	Researcher	IFGC
Giorgio	Giaccone			Division of Neurology V and Neuropathology, Fondazione IRCCS Istituto Neurologico Carlo Besta	Milan, Italy	Researcher	IFGC
James	Rowe			Cambridge University Department of Clinical Neurosciences	Cambridge, UK	Researcher	IFGC
Johannes	Schlachetzki			University of California San Diego, Department of Cellular & Molecular Medicine	La Jolla, CA, USA	Researcher	IFGC

Supplemental Online Content: Nonauthor Collaborators
*First name, last name, and suffix (if applicable) are required and will appear in PubMed.

***First Name and Middle Initial(s)**	***Last Name**	***Suffix (eg, Jr, III)**	Academic Degrees	Institution	Location (city, state/province, country)	Role or Contribution, eg, chair, principal investigator	Group (if more than 1 Group listed in the byline) and/or Subgroup (eg, Steering Committee)
James	Uphill			MRC Prion Unit, Department of Neurodegenerative Disease, UCL Institute of Neurology	London, UK	Researcher	IFGC
John	Collinge			MRC Prion Unit, Department of Neurodegenerative Disease, UCL Institute of Neurology	London, UK	Researcher	IFGC
Simon	Mead			MRC Prion Unit, Department of Neurodegenerative Disease, UCL Institute of Neurology	London, UK	Researcher	IFGC
Vivianna	Van Deerlin			University of Pennsylvania Perelman School of Medicine, Department of Pathology and Laboratory Medicine	Philadelphia, PA, USA	Researcher	IFGC
Anke	Marschhauser		MSc	Clinic for Cognitive Neurology, University Hospital Leipzig	Leipzig, Germany	Patholinguist	FTLD-C
Frank	Regenbrecht		MSc	Clinic for Cognitive Neurology, University Hospital Leipzig	Leipzig, Germany	Patholinguist	FTLD-C
Angelika	Thoene-Otto		PhD	Clinic for Cognitive Neurology, University Hospital Leipzig	Leipzig, Germany	Neuropsychologist	FTLD-C
Jannis	Gordulla		MSc	Clinic for Cognitive Neurology, University Hospital Leipzig	Leipzig, Germany	MD student	FTLD-C
Tommaso	Ballarini		PhD	Max Planck Institute for Human Cognitive and Brain Sciences	Leipzig, Germany	Researcher	FTLD-C
Annerose	Engel		PhD	Clinic for Cognitive Neurology, University Hospital Leipzig	Leipzig, Germany	Neuropsychologist	FTLD-C
Daniele	Pino		PhD	Clinic for Cognitive Neurology, University Hospital Leipzig	Leipzig, Germany	Patholinguist	FTLD-C
Dominique	Leuthold		BSc	Clinic for Cognitive Neurology, University Hospital Leipzig	Leipzig, Germany	Orthoptist	FTLD-C
Heike	Naumann		BSc	Clinic for Cognitive Neurology, University Hospital Leipzig	Leipzig, Germany	Orthoptist	FTLD-C

Supplemental Online Content: Nonauthor Collaborators
*First name, last name, and suffix (if applicable) are required and will appear in PubMed.

***First Name and Middle Initial(s)**	***Last Name**	***Suffix (eg, Jr, III)**	Academic Degrees	Institution	Location (city, state/province, country)	Role or Contribution, eg, chair, principal investigator	Group (if more than 1 Group listed in the byline) and/or Subgroup (eg, Steering Committee)
Murray	Grossman		MD	University of Pennsylvania Perelman School of Medicine, Department of Neurology and Penn Frontotemporal Degeneration Center	Philadelphia, PA, USA	Professor in Neurology	IFGC
John Q	Trojanowski		PhD	University of Pennsylvania Perelman School of Medicine, Department of Pathology and Laboratory Medicine	Philadelphia, PA, USA	Researcher	IFGC
Julie	van der Zee		PhD	Neurodegenerative Brain Diseases group, VIB-UAntwerp Center of Molecular Neurology	Antwerp, Belgium	Researcher	IFGC
Christine	Van Broeckhofen		PhD	Neurodegenerative Brain Diseases group, VIB-UAntwerp Center of Molecular Neurology	Antwerp, Belgium	Researcher	IFGC
Stefano F	Cappa		MD	Neurorehabilitation Unit, Dept. Of Clinical Neuroscience, Vita-Salute University and San Raffaele Scientific Institute,	Milan, Italy	Professor in Neurology	IFGC
Isabelle	Le Ber		MD	Hôpital de la Salpêtrière, Département de neurologie-centre de références des démences rares, Paris, France	Paris, France	Neurologist	IFGC
Didier	Hannequin		MD	Service de Neurologie, Inserm U1079, CNR-MAJ, Rouen University Hospital	Rouen, France	Neurologist	IFGC
Véronique	Golfier		MD	Service de neurologie, CH Saint Brieuc	Saint Brieuc, France	Neurologist	IFGC
Martine	Vercelletto		MD	Service de neurologie	CHU Nantes, France	Neurologist	IFGC
Alexis	Brice		MD	Hôpital de la Salpêtrière, Département de neurologie-centre de références des démences rares, Paris, France	Paris, France	Professor in Medical Genetics	IFGC

Supplemental Online Content: Nonauthor Collaborators
*First name, last name, and suffix (if applicable) are required and will appear in PubMed.

***First Name and Middle Initial(s)**	***Last Name**	***Suffix (eg, Jr, III)**	Academic Degrees	Institution	Location (city, state/province, country)	Role or Contribution, eg, chair, principal investigator	Group (if more than 1 Group listed in the byline) and/or Subgroup (eg, Steering Committee)
Benedetta	Nacmias		PhD	Department of Neurosciences, Psychology, Drug Research and Child Health (NEUROFARBA) University of Florence	Florence, Italy	Neuogenetics scientist	IFGC
Sandro	Sorbi		MD	Department of Neurosciences, Psychology, Drug Research and Child Health (NEUROFARBA) University of Florence and IRCCS "Don Carlo Gnocchi"	Florence, Italy	Professor of Neurology	IFGC
Silvia	Bagnoli			Department of Neurosciences, Psychology, Drug Research and Child Health (NEUROFARBA) University of Florence	Florence, Italy	Researcher	IFGC
Irene	Piaceri		PhD	Department of Neurosciences, Psychology, Drug Research and Child Health (NEUROFARBA University of Florence	Florence, Italy	Researcher	IFGC
Jørgen E	Nielsen		PhD	Danish Dementia Research Centre, Neurogenetics Clinic, Department of Neurology, Rigshospitalet, Copenhagen University Hospital	Copenhagen, Denmark	Neurologist	IFGC
Lena E	Hjermind		MD, PhD	Danish Dementia Research Centre, Neurogenetics Clinic, Department of Neurology, Rigshospitalet, Copenhagen University Hospital	Copenhagen, Denmark	Neurologist	IFGC
Matthias	Riemenschneider		MD	Saarland University Hospital, Department for Psychiatry & Psychotherapy	Homburg/Saar, Germany	Professor of Neurology	IFGC
Manuel	Mayhaus		PhD	Saarland University, Laboratory for Neurogenetics	Homburg/Saar, Germany	Researcher	IFGC

Supplemental Online Content: Nonauthor Collaborators
*First name, last name, and suffix (if applicable) are required and will appear in PubMed.

***First Name and Middle Initial(s)**	***Last Name**	***Suffix (eg, Jr, III)**	Academic Degrees	Institution	Location (city, state/province, country)	Role or Contribution, eg, chair, principal investigator	Group (if more than 1 Group listed in the byline) and/or Subgroup (eg, Steering Committee)
Bernd	Ibach		MD	University Regensburg, Department of Psychiatry, Psychotherapy and Psychosomatics	Regensburg, Germany	Professor of Psychiatry	IFGC
Gilles	Gasparoni		PhD	Saarland University, Laboratory for Neurogenetics	Homburg/Saar, Germany	Researcher	IFGC
Sabrina	Pichler			Saarland University, Laboratory for Neurogenetics	Homburg/Saar, Germany	Researcher	IFGC
Wei	Gu		PhD	Saarland University, Laboratory for Neurogenetics	Homburg/Saar, Germany	Researcher	IFGC
Martin N	Rossor		MD	Dementia Research Centre, Department of Neurodegenerative Disease, UCL Institute of Neurology	London, UK	Professor of Neurology	IFGC
Nick C	Fox		MD	Dementia Research Centre, Department of Neurodegenerative Disease, UCL Institute of Neurology	London, UK	Professor of Neurology	IFGC
Jason D	Warren		PhD	Dementia Research Centre, Department of Neurodegenerative Disease, UCL Institute of Neurology	London, UK	Professor of Neurology	IFGC
Maria Grazia	Spillantini		PhD	University of Cambridge, Department of Clinical Neurosciences, John Van Geest Brain Repair Centre	Cambridge, UK	Professor of Molecular Neurology	IFGC
Huw R	Morris		PhD	UCL, Department of Molecular Neuroscience, Russell Square House	London, UK	Professor of clinical neuroscience	IFGC
Patrizia	Rizzu		PhD	German Center for Neurodegenerative Diseases-Tübingen	Tuebingen, Germany	Researcher	IFGC
Peter	Heutink		PhD	German Center for Neurodegenerative Diseases-Tübingen	Tuebingen, Germany	Researcher	IFGC

*First name, last name, and suffix (if applicable) are required and will appear in PubMed.

***First Name and Middle Initial(s)**	***Last Name**	***Suffix (eg, Jr, III)**	Academic Degrees	Institution	Location (city, state/province, country)	Role or Contribution, eg, chair, principal investigator	Group (if more than 1 Group listed in the byline) and/or Subgroup (eg, Steering Committee)
Julie S	Snowden		PhD	Institute of Brain, Behaviour and Mental Health, Faculty of Medical and Human Sciences, University of Manchester	Manchester, UK	Professor Neuropsychology	IFGC
Sara	Rollinson		PhD	Institute of Brain, Behaviour and Mental Health, Faculty of Medical and Human Sciences, University of Manchester	Manchester, UK	Researcher	IFGC
Anna	Richardson		MD	Salford Royal Foundation Trust, Faculty of Medical and Human Sciences, University of Manchester	Manchester, UK	Psychiatrist	IFGC
Alexander	Gerhard		MD	Institute of Brain, Behaviour and Mental Health, The University of Manchester	Manchester, UK	Researcher	IFGC
Amalia C	Bruni		MD	Regional Neurogenetic Centre, ASPCZ	Lamezia Terme, Italy	Neorologist	IFGC
Raffaele	Maletta		MD	Regional Neurogenetic Centre, ASPCZ	Lamezia Terme, Italy	Pathologist	IFGC
Francesca	Frangipane		MD	Regional Neurogenetic Centre, ASPCZ	Lamezia Terme, Italy	Neurologist	IFGC
Chiara	Cupidi		MD	Regional Neurogenetic Centre, ASPCZ	Lamezia Terme, Italy	Neurologist	IFGC
Livia	Bernardi		PhD	Regional Neurogenetic Centre, ASPCZ	Lamezia Terme, Italy	Researcher	IFGC
Maria	Anfossi		PhD	Regional Neurogenetic Centre, ASPCZ	Lamezia Terme, Italy	Researcher	IFGC
Maura	Gallo		PhD	Regional Neurogenetic Centre, ASPCZ	Lamezia Terme, Italy	Researcher	IFGC
Maria Elena	Conidi		PhD	Regional Neurogenetic Centre, ASPCZ	Lamezia Terme, Italy	Researcher	IFGC
Nicoletta	Smirne		BSc	Regional Neurogenetic Centre, ASPCZ	Lamezia Terme, Italy	Researcher	IFGC
Rosa	Rademakers		PhD	Department of Neuroscience, Mayo Clinic Jacksonville	Jacksonville, FL, USA	Neurogeneticist	IFGC
Matt	Baker		PhD	Department of Neuroscience, Mayo Clinic Jacksonville	Jacksonville, FL, USA	Researcher	IFGC
Dennis W	Dickson		MD	Department of Neuroscience, Mayo Clinic Jacksonville	Jacksonville, FL, USA	Researcher	IFGC
Neill R	Graff-Radford		MD	Department of Neurology, Mayo Clinic Jacksonville	Jacksonville, FL, USA	Researcher	IFGC
Ronald C	Petersen		MD	Department of Neurology, Mayo Clinic Rochester	Rochester, MN, USA	Professor of Neurology	IFGC

Supplemental Online Content: Nonauthor Collaborators
*First name, last name, and suffix (if applicable) are required and will appear in PubMed.

***First Name and Middle Initial(s)**	***Last Name**	***Suffix (eg, Jr, III)**	Academic Degrees	Institution	Location (city, state/province, country)	Role or Contribution, eg, chair, principal investigator	Group (if more than 1 Group listed in the byline) and/or Subgroup (eg, Steering Committee)
David	Knopman		MD	Department of Neurology, Mayo Clinic Rochester	Rochester, MN, USA	Neurologist	IFGC
Keith A	Josephs		MD	Department of Neurology, Mayo Clinic Rochester	Rochester, MN, USA	Neurologist	IFGC
Bradley F	Boeve		MD	Department of Neurology, Mayo Clinic Rochester	Rochester, MN, USA	Neurologist	IFGC
Joseph E	Parisi		MD	Department of Neurology, Mayo Clinic Rochester	Rochester, MN, USA	Pathologst	IFGC
William W	Seeley		MD	Department of Neurology, University of California	San Francisco, CA, USA	Professor of Neurology	IFGC
Bruce L	Miller		MD	Memory and Aging Center, Department of Neurology, University of California	San Francisco, CA, USA	Professor of Neurology	IFGC
Anna M	Karydas			Memory and Aging Center, Department of Neurology, University of California	San Francisco, CA, USA	Researcher	IFGC
Howard	Rosen		MD	Memory and Aging Center, Department of Neurology, University of California	San Francisco, CA, USA	Professor of Neurology	IFGC
John C	van Swieten		MD, PhD	Department of Neurology, Erasmus Medical Centre	Rotterdam, The Netherlands	Professor of genetics	IFGC
Elise GP	Dopper		PhD	Department of Neurology, Erasmus Medical Centre	Rotterdam, The Netherlands	Researcher	IFGC
Harro	Seelaar		PhD	Department of Neurology, Erasmus Medical Centre	Rotterdam, The Netherlands	Neurologist	IFGC
Yolande AL	Pijnenburg		MD, PhD	Alzheimer Centre and department of neurology, VU University medical centre	Amsterdam, The Netherlands	Professor of Neurology	IFGC
Philip	Scheltens		MD, PhD	Alzheimer Centre and department of neurology, VU University medical centre	Amsterdam, The Netherlands	Professor of Neurology	IFGC
Giancarlo	Logroscino		MD	Department of Basic Medical Sciences, Neurosciences and Sense Organs of the "Aldo Moro" University of Bari	Bari, Italy	Professor of Neurology	IFGC

Supplemental Online Content: Nonauthor Collaborators
*First name, last name, and suffix (if applicable) are required and will appear in PubMed.

***First Name and Middle Initial(s)**	***Last Name**	***Suffix (eg, Jr, III)**	Academic Degrees	Institution	Location (city, state/province, country)	Role or Contribution, eg, chair, principal investigator	Group (if more than 1 Group listed in the byline) and/or Subgroup (eg, Steering Committee)
Rosa	Capozzo		MD	Department of Basic Medical Sciences, Neurosciences and Sense Organs of the "Aldo Moro" University of Bari	Bari, Italy	Neurologist	IFGC
Valeria	Novelli		MD	Medical Genetics Unit, Fondazione Policlinico Universitario A. Gemelli	Rome, Italy	Researcher	IFGC
Annibale A	Puca		MD	Cardiovascular Research Unit, IRCCS Multimedica, Milan, Italy; Department of Medicine and Surgery, University of Salerno	Baronissi, SA, Italy	Professor of Medical Genetics	IFGC
Massimo	Franceschi		MD	Neurology Dept, IRCCS Multimedica	Milan, Italy	Neurologist	IFGC
Alfredo	Postiglione		MD	Department of Clinical Medicine and Surgery, University of Naples Federico II	Naples, Italy	Researcher	IFGC
Graziella	Milan		MD	Geriatric Center Frullone- ASL Napoli 1 Centro	Naples, Italy	Neurologist	IFGC
Paolo	Sorrentino		MD	Geriatric Center Frullone- ASL Napoli 1 Centro	Naples, Italy	Researcher	IFGC
Mark	Kristiansen		PhD	UCL Genomics, Institute of Child Health (ICH) UCL	London, UK	Researcher	IFGC
Huei-Hsin	Chiang		PhD	Karolinska Institutet, Dept NVS, Alzheimer Research Center	Stockholm, Sweden	Researcher	IFGC
Caroline	Graff		PhD	Karolinska Institutet, Dept NVS, Alzheimer Research Center	Stockholm, Sweden	Professor of genetic dementia	IFGC
Florence	Pasquier		MD	University of Lille	Lille, France	Professor	IFGC
Adeline	Rollin		MD	University of Lille	Lille, France	Neurologist	IFGC
Vincent	Deramecourt		MD	University of Lille	Lille, France	Neurologist	IFGC
Thibaud	Lebouvier		MD	University of Lille	Lille, France	Professor of Neurology	IFGC
Dimitrios	Kapogiannis		MD	National Institute on Aging (NIA/NIH)	Baltimore, MD, USA	Neurologist	IFGC
Luigi	Ferrucci		MD	Clinical Research Branch, National Institute on Aging	Baltimore, MD, USA	Scientific director NIA	IFGC

Supplemental Online Content: Nonauthor Collaborators
*First name, last name, and suffix (if applicable) are required and will appear in PubMed.

***First Name and Middle Initial(s)**	***Last Name**	***Suffix (eg, Jr, III)**	Academic Degrees	Institution	Location (city, state/province, country)	Role or Contribution, eg, chair, principal investigator	Group (if more than 1 Group listed in the byline) and/or Subgroup (eg, Steering Committee)
Stuart	Pickering-Brown		PhD	Institute of Brain, Behaviour and Mental Health, Faculty of Medical and Human Sciences, University of Manchester	Manchester, UK	Professor of Neurogenetics	IFGC
Andrew B	Singleton		PhD	Laboratory of Neurogenetics, National Institute on Aging, National Institutes of Health	Bethesda, MD, USA	Researcher	IFGC
John	Hardy		PhD	Department of Molecular Neuroscience, UCL	London, UK	Professor of mol.biology	IFGC
Parastoo	Momeni		PhD	Laboratory of Neurogenetics, Department of Internal Medicine, Texas Tech University Health Science Center	Lubbock, Texas, USA	Researcher	IFGC
Henryk	Barthel		MD	Nuclear Medicine Department, University Hospital Leipzig	Leipzig, Germany	Professor	FTLD-C
Semler	Elisa		PhD	Department of Neurology, University clinic Ulm	Ulm, Germany	researcher	FTLD-C
Lombardi	Jolina		PhD	Department of Neurology, University clinic Ulm	ulm, Germany	researcher	FTLD-C
von Arnim	Christine		MD	Department of Geriatrics, University clinic Goettingen	Goettingen, Germany	Professor	FTLD-C
Oberahauser	Felix		MD	Department of Psychiatry, University clinic Erlangen	Erlangen, Germany	researcher	FTLD-C
Schumacher	Kai		MD	Department of Psychiatry, University clinic Erlangen	Erlangen, Germany	Researcher	FTLD-C
Lehmbeck	Jan		MD	Department of Psychiatry, University clinic Erlangen	erlangen, Germany	researcher	FTLD-C
Maler	Juan-Manuel		MD	Department of Psychiatry, University clinic Erlangen	Erlangen, Germany	Professor	FTLD-C
Richter-Schmidinger	Tanja		PhD	Department of Psychiatry, University clinic Erlangen	erlangen, Germany	Researcher	FTLD-C

Supplemental Online Content: Nonauthor Collaborators
*First name, last name, and suffix (if applicable) are required and will appear in PubMed.

***First Name and Middle Initial(s)**	***Last Name**	***Suffix (eg, Jr, III)**	Academic Degrees	Institution	Location (city, state/province, country)	Role or Contribution, eg, chair, principal investigator	Group (if more than 1 Group listed in the byline) and/or Subgroup (eg, Steering Committee)
Hammer-Kasperei	Anke		PhD	Department of Psychiatry, University clinic Erlangen	Erlangen, Germany	Researcher	FTLD-C
Oberstein	Timo		MD	Department of Psychiatry, University clinic Erlangen	Erlangen, Germany	Researcher	FTLD-C
Müller-Sarnowski	Felix		MD	Department of Psychiatry, TU Munich	Munich, Germany	Researcher	FTLD-C
Roßmeier	Carola		MD	Department of Psychiatry, TU Munich	Munich, Germany	Researcher	FTLD-C
Ludolph	Albert		MD	Department of Neurology, University clinic Ulm	Ulm, Germany	Professor	FTLD-C
Kassubek	Jan		MD	Department of Neurology, University clinic Ulm	Ulm, Germany	Professor	FTLD-C
Schneider	Anja		MD	Department of Psychiatry, University clinic Bonn	Bonn, Germany	Professor	FTLD-C
Levin	Johannes		MD	Department of Neurology, University clinic LMU Munich	Munich, Germany	Professor	FTLD-C
